# Proton-Coupled Electron Transfer Ring Opening of Cycloalkanols Followed by a Giese Radical Addition Enabled by an Electron Donor–Acceptor Complex

**DOI:** 10.1021/acs.orglett.4c01443

**Published:** 2024-05-22

**Authors:** Benedetta Carli, Noelia Salaverri, Lara Martinez-Fernandez, Marta Goicuría, José Alemán, Leyre Marzo

**Affiliations:** Organic Chemistry Department (Módulo 1), Universidad Autónoma de Madrid, Calle Francisco Tomás y Valiente 7, 28049 Madrid, Spain

## Abstract

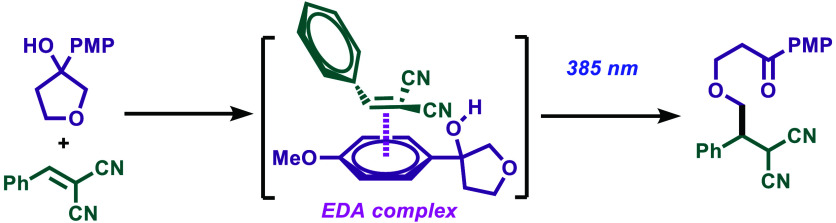

Herein, we describe the formation of an electron donor–acceptor (EDA) complex between electron-rich cycloalkanols and electron-deficient alkenes that triggers the proton-coupled electron transfer ring opening of strained and unstrained cycloalkanols without the need for an external photocatalyst. This activation generates a remote alkyl radical that undergoes a Giese reaction with the Michael acceptor in an efficient manner. Mechanistic investigations corroborate both the formation of the EDA complex and the consecutive Giese reaction.

Visible light photocatalysis appeared in the past decade as an extraordinary tool for the formation of new C–C bonds under very mild and sustainable conditions.^1^ Among the different activation modes that this research area has to offer, the formation of visible-light-active electron donor–acceptor (EDA) complexes has recently been recognized as a powerful photochemical approach for synthesis. This strategy exploits the interaction of an electron donor and an electron acceptor molecule that in the ground state form a molecular aggregation, which absorbs at longer wavelengths than the isolated molecules (Figure 1a).^2^ This complex becomes excited upon light irradiation, triggering a single-electron transfer (SET) event from the donor to the acceptor. This yields a radical anion and a radical cation that react to form the final product. In the past decade, this strategy has been broadly employed in organic synthesis to reduce and functionalize alkyl or aryl halides, N–O bond activation, or α-functionalization of aldehydes, among many other transformations.^3^

**Figure 1:**
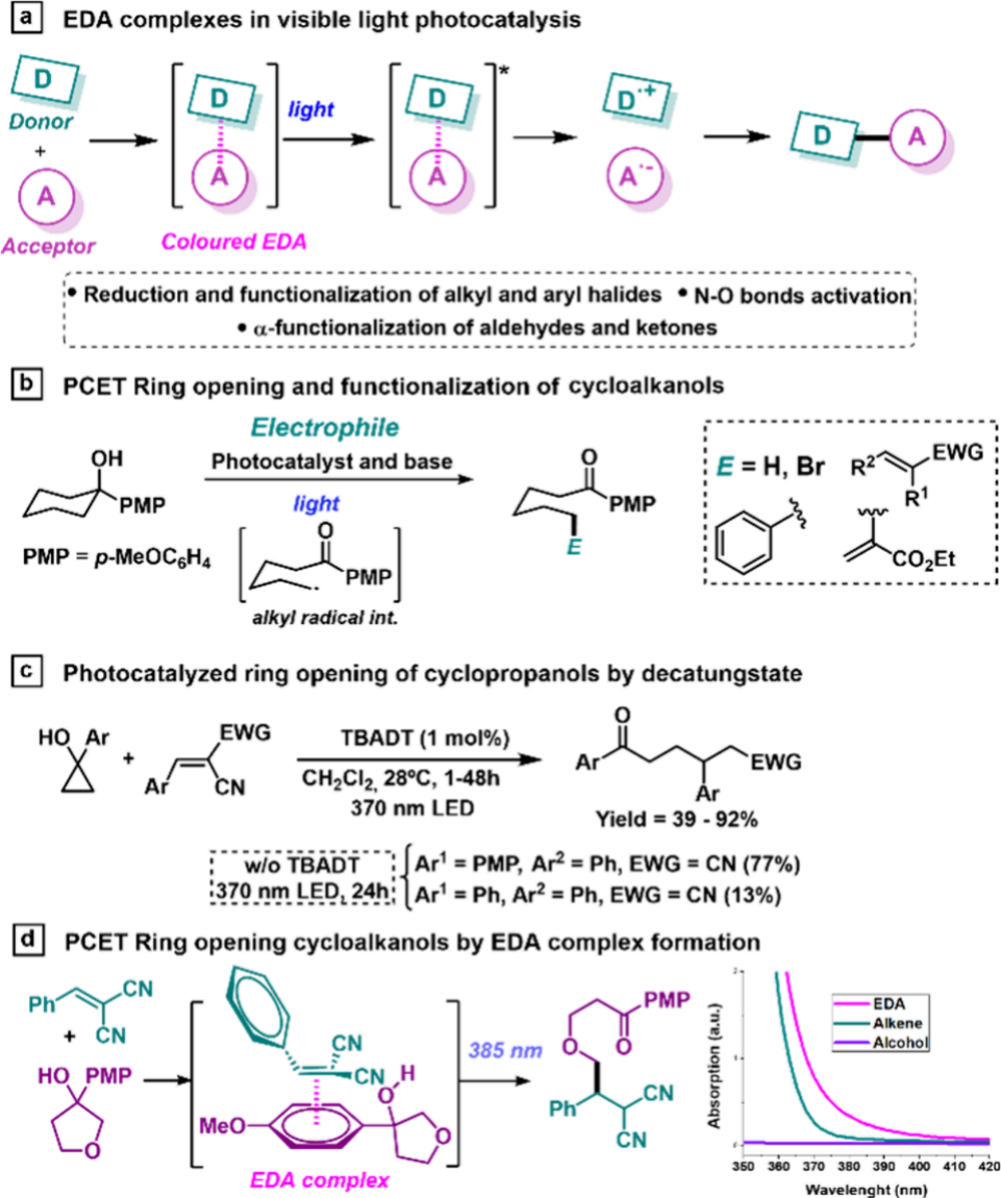
(a) EDA complexes in visible light photocatalysis, (b) PCET ring opening and functionalization of cycloalkanols, (c) photocatalyzed ring opening of cyclopropanols by decatungstate, and (d) PCET ring opening of cycloalkanols by EDA complex formation.

Another activation mode that is gaining more relevance in the field of visible light photocatalysis is the proton-coupled electron transfer (PCET), because it allows for the activation of functional groups with very high redox potentials.^4^ The PCET strategy is based on the concerted transfer of a proton and an electron in the same step, which compensates for the unfavorable energetics of those steps taking place in a sequential manner. Although this strategy is traditionally employed in biological redox reactions, it was not implemented in organic synthesis until the past decade.^5^ An example of the potential that this activation mode has to offer is the isomerization of unstrained cycloalkanols to linear ketones described by Knowles and co-workers in 2016 (Figure 1b).^6^ Alcohols present a very high oxidation potential not achievable by most of the photocatalytic systems currently described in the literature.^7^ In this process, the photocatalyst oxidizes the *para*-methoxyphenyl (PMP) residue, and then, the PCET event takes place through a concerted deprotonation by the base and SET from the alcohol to the PMP radical cation. The resulting alkoxide radical evolves through β-C–C bond scission to yield the transient alkyl radical intermediate (Figure 1b). This radical intermediate has undergone different reactions, such as halogenation,^8^ arylation,^9^ allylation,^10^ cyclization,^11^ and Giese additions.^12^ All of these transformations have in common the use of an external photocatalyst and a base to trigger the PCET activation of cycloalkanol. Nevertheless, we overviewed that given the electron-rich nature of cycloalkanols and the electron-deficient nature of the Michael acceptors, the formation of an EDA complex must be highly favored. Indeed, when we studied the absorption spectrum of cycloalkanol, the electron-deficient alkene, and both components together, we observed a bathochromic shift that confirmed the formation of the EDA complex (right spectrum in Figure 1d). In the process of our investigations, Kananovich and Ošeka described the ring opening of cyclopropanols using tetrabutylammonium decatungstate (TBADT) as a photocatalyst (Figure 1c).^7b^ In this work, they also observed the formation of an EDA complex between PMP-substituted cyclopropyl alcohol and benzylidene malononitrile. They also describe the formation of the EDA complex with phenylcyclopropyl alcohol and dimethyl fumarate. Herein, we describe the PCET activation of strained and unstrained cycloalkanols through the formation of a visible-light-active EDA complex between cycloalkanols and electron-deficient alkenes, avoiding the use of an external photocatalyst, followed by a Giese radical addition (Figure 1c).

With this preliminary observation, we began to study the reaction between compounds **1a** and **2a** (Table 1). First, we examined the reaction under different irradiation wavelengths. According to the initial absorption spectrum (Figure 1c), the EDA complex absorbs at 400, 385, and 365 nm. However, at 365 nm, the Michael acceptor also absorb and the [2 + 2] photocycloaddition reaction of compound **2a** with itself could occur. The reaction was carried out with a 420 or 365 nm light-emitting diode (LED), and the formation of product **3a** was not found (entries 2 and 4 in Table 1). In addition, under 365 nm irradiation, the formation of cyclobutane was observed, resulting from the [2 + 2] photocycloaddition of compound **2a**. Delightfully, under 400 nm LED, product **3a** was formed in a 17% yield that could be increased until a 80% yield under 385 nm LED irradiation (entries 1 and 3 in Table 1). Moreover, this EDA complex was only formed using CH_3_CN as the solvent, whereas with CH_2_Cl_2_, tetrahydrofuran (THF), ethanol, toluene, *N*,*N*-dimethylformamide (DMF), or dimethyl sulfoxide (DMSO), the reaction did not take place (entry 5 in Table 1). Decreasing or increasing the concentration and changes in the compounds **1a**/**2a** ratio afforded lower conversions (entries 6–10 in Table 1). In the absence of light, the reaction does not take place, and no increase in the yield was observed in the presence of 25 mol % lutidine (entries 11 and 12 in Table 1).

**Table 1:**
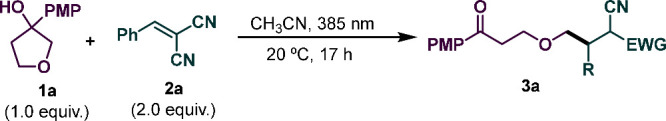
Optimization of the Giese Reaction under EDA Complex Formation^a^

entry	variations over the standard reaction conditions	yield (%)^b^
1	none	80^c^
2	420 nm LED	0
3	400 nm LED	17
4	365 nm LED	0^d^
5	CH_2_Cl_2_, THF, EtOH, toluene, DMF, or DMSO instead of CH_3_CN as the solvent	0
6	0.5 mL of CH_3_CN (0.2 M)	25
7	2 mL of CH_3_CN (0.05 M)	18
8	1:1 ratio of compounds **1a**/**2a**	30
9	1:1 ratio of compounds **1a**/**2a**^e^	0
10	1:3 ratio of compounds **1a**/**2a**	0
11	2:1 ratio of compounds **1a**/**2a**	10
12	1:2 ratio of compounds **1a**/**2a**^e^	42
13	no light	0

a Reaction conditions: compound **1a** (0.1 mmol), compound **2a** (0.2 mmol), CH_3_CN (1 mL, 0.1 M), 385 nm, 20 °C, and 17 h.

b Yield was determined by ^1^H NMR with trimethoxybenzene.

c Isolated yield.

d In this reaction, we observed traces of the [2 + 2] photocycloaddition of compound **2a**.

e In the presence of 25 mol % 2.6-lutidine.

Once the conditions were optimized, we proceeded to study the scope of the reaction with different alcohols **1** and electron-deficient alkenes **2** (Scheme 1). In some cases, to achieve better yields, it was necessary to use a catalytic amount of base to promote the PCET activation (see products **3c** and **3e**; Scheme 1), and/or increase the irradiation time, as indicated in Scheme 1.

**Scheme 1:**
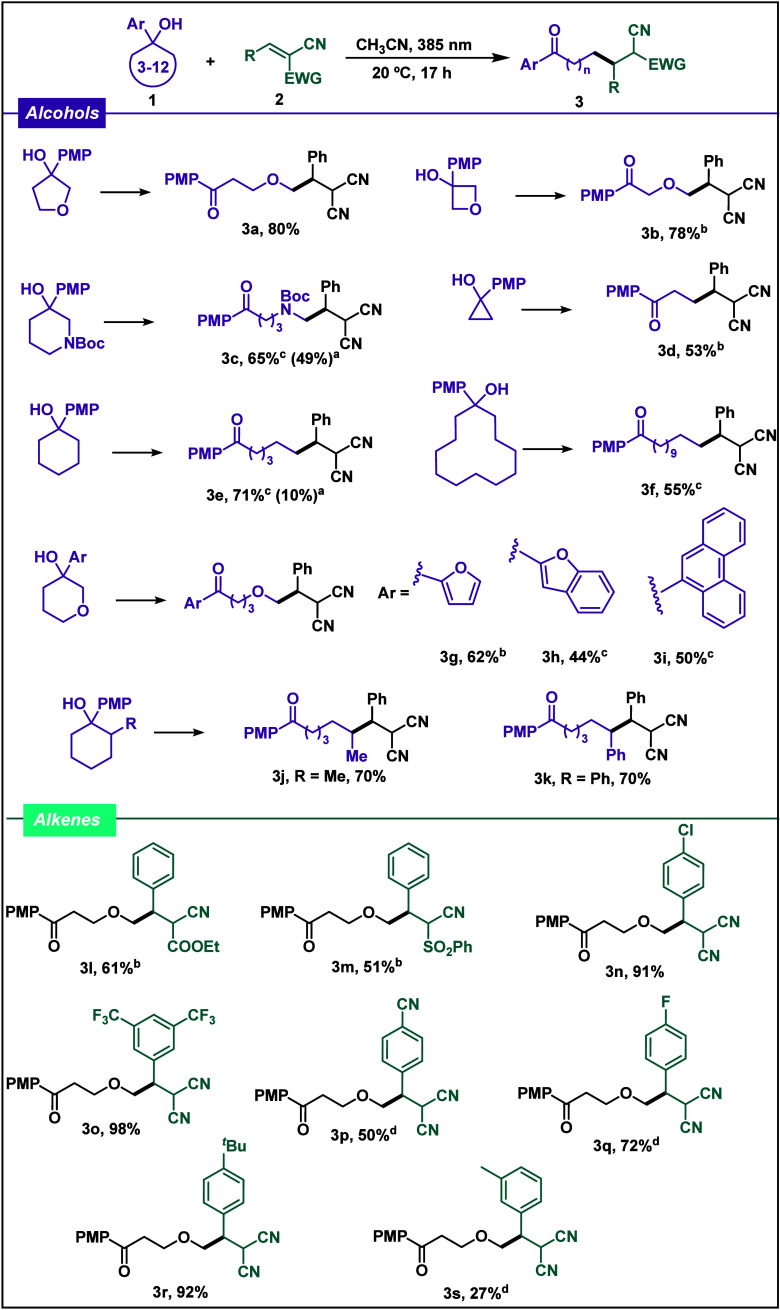
Study of the Scope of the Giese Reaction under EDA Complex Formation Reaction performed using 0.1 mmol of compound **1**, 0.2 mmol of compound **2**, and 1 mL of CH_3_CN (0.1 M), under 385 nm LED irradiation, at 20 °C, for 17 h. This reaction is carried out in the presence of 25 mol % 2,6-lutidine and 24 h of irradiation. This reaction is carried out in the presence of 25 mol % 2,6-lutidine. After 24 h of irradiation.

Initially, a variety of structurally diverse cycloalkanols with different ring sizes with or without a stabilizing β-heteroatom were studied. Cycloalkanols from 4- to 6-membered rings containing either oxygen or a β-NBoc group afforded the final product in a good to very good yield (**3a**–**3c**; Scheme 1). Other cycloalkanols from 3- to 12-membered rings without a β-heteroatom were also studied, affording the final products in moderate to excellent yields (**3d**–**3f**). The presence of a methyl or phenyl substituent in the α position, able to stabilize the resulting alkyl radical intermediate, also afforded good yields, obtaining products **3j** and **3k** as a mixture of diasteroisomers. Finally, the reaction can also be performed with other oxidizable heteroarenes instead of the PMP group, such as a furane, benzofurane, or phenanthrene, that afforded products **3g**–**3i** in good yields. Then, we proceeded to explore the scope of the reaction with different electron-deficient alkenes (alkenes in Scheme 1). The presence of two electron-withdrawing groups (EWGs) was always required; one must be a nitrile, while the second can be an ester or a sulfone. Thus, products **3l** and **3m** were prepared in good yields. Afterward, we studied the presence of different substituents in the phenyl ring of the Michael acceptor. Delightfully, the presence of electron-withdrawing or soft electron-donating substituents in the *ortho*, *meta*, or *para* position of phenyl did not affect the reactivity, giving rise to the final products in good to very good yields (**3n**–**3s**). Other cycloalkanols and alkenes were studied with unsuccessful results (see section 4 of the Supporting Information). Further ultraviolet–visible (UV–vis) absorption studies revealed that no EDA formation is taking place for those compounds, which explains the lack of reactivity (see section 5 of the Supporting Information).

As it is known, the scalability of a photochemical reaction under batch conditions is not always possible as a result of the intrinsic limitations of the setups employed and the poor light penetration, and so is the reaction studied herein.^13^ Flow chemistry is gaining interest among photochemists because it allows us to carry out photochemical transformations at a large scale in a highly efficient manner. Thus, we decided to optimize this transformation under flow conditions (Scheme 2). Using 390 nm Kessil lamps at both sides of the photoreactor, it was possible to perform the photochemical transformation at a scale 20 times higher than that for the batch reactions (2 mmol) with a 66% yield.

**Scheme 2:**
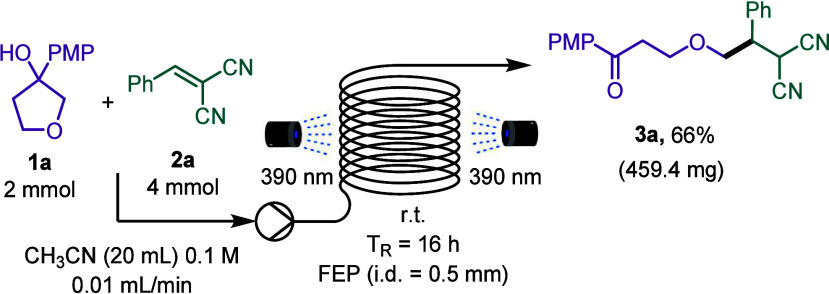
Flow Reaction Conditions for the Synthesis of Product **3a**

Finally, mechanistic investigations were carried out, paying particular attention to the EDA complex characterization (Scheme 3). The formation of the EDA complex was examined by UV–vis absorption spectrometry (Scheme 3B). While alcohol **1a** or alkene **2a** do not present a significant absorption at 385 nm, the mixture presents a bathochromic shift that corresponds to the formation of an EDA complex. In fact, it was possible to obtain the absorption band of the EDA complex through subtraction of the absorption spectrum of compounds **1a** and **2a** from the mixture, which presents a maximum at 360 nm with a significant absorption at 385 nm (Scheme 3B). In agreement, TD-M062X calculations predict lower absorption energies for the S_1_ state, once the EDA complex has been formed in comparison to the alcohol or alkene species (see the Supporting Information). The stoichiometry of the EDA complex was investigated by a Job plot analysis^14^ of UV–vis that afforded a molar fraction of compound **2a** of 58% (Scheme 3C). This result suggests either a 1:1 or 1:2 ratio of compounds **1a**/**2a** in the EDA complex. To further elucidate the stoichiometry of the EDA complex, theoretical calculations were performed, and from the computed free energies obtained, it can be inferred that the 1:1 stoichiometry of both compounds is the most likely. In addition, the association constant was calculated to be 0.1 M^–1^ in CH_3_CN with the Benesi–Hildebrand method.^15^ Moreover, nuclear magnetic resonance (NMR) titration experiments revealed an interaction between the Michael acceptor and the PMP group in the ground state (Scheme 3D). The ^1^H NMR spectrum shows a downfield shifting of the signals corresponding to C–H of alkene (8.1 ppm), aromatic H in the *ortho* position to the methoxy group (6.9 ppm), and the signal that corresponds to OH (3.3 ppm) (Scheme 3D).

**Scheme 3:**
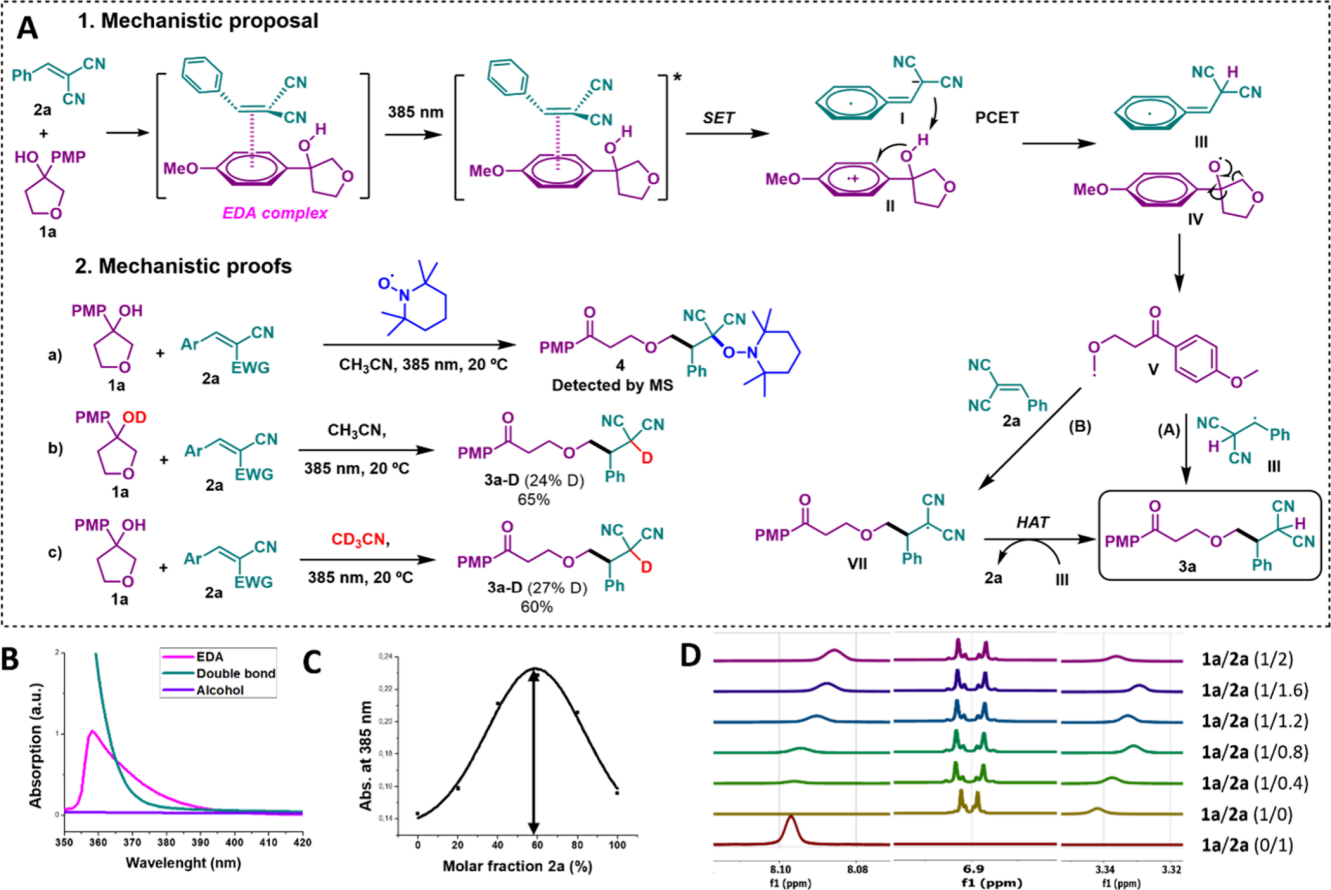
Mechanistic Studies of the EDA-Promoted PCET Ring Opening Followed by a Giese Addition A, mechanistic proposal and mechanistic proofs; B, absorption spectrum of EDA; C, Job plot; and D, ^1^H NMR titration experiments.

With all of this evidence in hand, our mechanistic proposal starts with the formation of the EDA complex in which the double bond from the Michael acceptor interacts with the PMP ring (Scheme 3A). This EDA is photoactive at 385 nm and, upon irradiation, absorbs one photon, reaching the excited state. Then, SET from the PMP substituent to the Michael acceptor affords the radical cation in PMP **II** and the radical anion **I**. The radical cation undergoes a PCET in which the radical anion of malononitrile **I** acts as the base deprotonating the alcohol and one electron is transferred from the alcohol to the PMP radical cation, yielding intermediates **III** and **IV**. Alkoxide radical **IV** evolves through β-C–C bond scission to afford alkyl radical **V** that can follow two possible reaction mechanisms. On the one hand, this alkyl radical **V** can undergo radical–radical recombination with intermediate **III** to directly afford product **3a** (pathway A in Scheme 3). On the other hand, alkyl radical **V** can be added to a second molecule of alkene **2a**, to afford intermediate **VII**. This intermediate undergoes hydrogen atom transfer (HAT) with intermediate **III** or CH_3_CN to afford final product **3a** (pathway B in Scheme 3). To obtain further insight into the reaction mechanism, the reaction was carried out in the presence of (2,2,6,6-tetramethylpiperidin-1-yl)oxyl (TEMPO) (Mechanistic proofs in Scheme 3A.2a). Gratifyingly, it was possible to detect by mass spectrometry the formation of compound **4**, that is, the TEMPO adduct of the radical intermediate **VII**. In addition, the reaction was performed starting from deuterated alcohol or using CD_3_CN as the solvent of the reaction (Mechanistic proofs in Scheme 3A.2b and c). In both cases, partial deuteration of product **3a** was observed suggesting pathway B as the most likely pathway, because deuterium can be taken in the HAT event from either deuterated intermediate **III** (formed upon deprotonation from deuterated alcohol) or the deuterated solvent. This result together with the TEMPO experiment suggests that pathway B is the most plausible pathway.

In conclusion, the implementation of PCET in photocatalysis has become a very powerful tool for the expansion of transformations achievable because it allows for the activation of functional groups with very high redox potentials. In this letter, we study the formation of an EDA complex between strained and unstrained cycloalkanols and Michael acceptors to trigger the PCET activation of the former using the power of 385 nm LEDs, without the need for an external photocatalyst. The scope of the reaction tolerates a large variety of cycloalkanols and electron-deficient alkenes bearing a nitrile and a second EWG. The implementation of the reaction under flow conditions allowed for the scaleup of the process with a good yield. Finally, mechanistic investigations, which also include theoretical calculations, revealed a 1:1 stoichiometry of the two components in the EDA complex followed by a Giese addition for the formation of the C–C bond.

## Data Availability

The data underlying this study are available in the published article and its Supporting Information.

## Supporting Information Available

Experimental procedures and spectral data (PDF)

## Dedication

This work is dedicated to Prof. Carmen Maestro on the occasion of her retirement.
