# Supplementary material for: Proton-Coupled Electron Transfer Ring Opening of Cycloalkanols Followed by a Giese Radical Addition Enabled by an Electron Donor–Acceptor Complex
Source: Org Lett. 2024 May 22;26(21):4542–7. doi: 10.1021/acs.orglett.4c01443 (PMC11148845; doi:10.1021/acs.orglett.4c01443)
Supplement: Supplementary file 1 — ol4c01443_si_001.pdf [file ol4c01443_si_001.pdf]

Supplementary Information

PCET Ring Opening of Cycloalkanols followed by a Giese Radical Addition Enabled by an EDA Complex.

Benedetta Carli,[a] Noelia Salaverri, [a] Lara Martinez-Fernandez, [c] Marta Goicuría,[a] José Alemán,* [a, b] Leyre Marzo *[a, b]

[a] B. Carli, N. Salaverri, L. Marzo, J. Alemán
Organic Chemistry Department (Módulo 1)
Universidad Autónoma de Madrid,
Calle Francisco Tomás y Valiente 7, 28049 Madrid (Spain).
E-mail:; jose.aleman@uam.es; leyre.marzo@uam.es

[b] L. Marzo, J. Alemán
Institute for Advanced Research in Chemical Sciences (IAdChem)
Universidad Autónoma de Madrid,
Calle Francisco Tomás y Valiente 7, 28049 Madrid (Spain).

[c] L. Martinez-Fernandez
Instituto de Química Física Blas Cabrera
Consejo Superior de Investigaciones Científicas
Calle Serrano 119, 28006, Madrid, Spain.

Table of contents

1. General methods and materials S3
2. General procedure for the synthesis and characterization of alcohols **1** S6
    2.1 General procedure A for the synthesis of alcohols (**1a-1j**)……………….S6
    2.2 Procedure and characterization of the synthesis of 1-methoxyphenyl)cyclopropan-1-ol (**1k**)……………………………………….....S10
3. General procedure for the synthesis and characterization of Michael acceptors **2**………………………………………………………………….S11
    3.1 Synthesis and characterization of α,β-unsaturated alkenes **2a-2g**……S11
    3.2 Synthesis and characterization of α,β-unsaturated alkenes **2h** and **2i**..S14
4. Limitations of the reaction……………………………………………………S15
5. UV-Vis absorption spectra..……….............………..…………………….S17
6. Mechanistic studies on the photocatalytic reaction………………………......S21
    6.1 Stoichiometry of the EDA complex (Job plot)………………….........….S21
    6.2 Determination of the association constant…………….……….….……S21
    6.3 ^{1}H-NMR titration experiment……………………………………….....….S19
    6.4 Deuterium labelling experiments………………………………………..S24
    6.5 Radical trapping experiment with TEMPO…………………………..…...S28
    6.6 Computational details. DFT calculations………..……………………..S24
7. Experimental procedures and characterizations of products **3**…………….…S32
8. General procedure for the photo-flow synthesis of product **3a**……………..S44
9. NMR Spectra ………………………..……………………………………..S45
10. References……………………………..……………………………….S76

1. General methods and materials

NMR spectra were acquired on a BRUKER AVANCE 300 or NEO 500 MHz spectrometer running at 300 or 500 MHz for ^{1}H, 75 or 125 MHz for ^{13}C, 282 or 471 MHz for ^{19}F, and are internally referenced to residual solvent signals ($CDCl_3$ referenced at δ 7.26 ppm for ^{1}H NMR and δ 77.2 ppm for ^{13}C NMR). Data for ^{1}H NMR are reported as follows: chemical shift (δ ppm), multiplicity (s = singlet, d = doublet, t = triplet, quint = quintuplet, m = multiplet), coupling constant (Hz) and integration. Data for ^{13}C and ^{19}F are reported in terms of chemical shift.

High-Resolution Mass Spectra (HRMS) were obtained on an Agilent Technologies 6120 Quadrupole LC/MS coupled with an SFC Agilent Technologies 1260 Infinity Series instrument for the MS (ESI) (Electrospray Ionization). MassWorks software version 4.0.0.0 (Cerno Bioscience) was used for the formula identification. MassWorks is an MS calibration software which calibrates isotope profiles to achieve high mass accuracy and enables elemental composition determination on conventional mass spectrometers of unit mass resolution allowing highly accurate comparisons between calibrated and theoretical spectra.

All continuous-flow experiments were carried out using a commercially available Syringe pump NE-1000 purchase from syringepump.com and a coil reactor (10 mL), made of FEP capillary tube Vapourtec 50-1581 (1/16”OD; 0.5 mm ID).

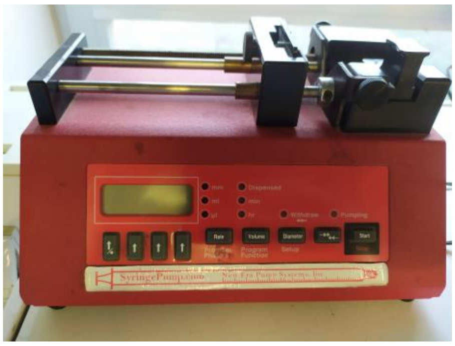

Figure S1. Syringe pump NE-1000.

A Kessil® PhotoReaction Lighting PR160L 390 nm and a cooling fan was used as the

photoflow setup. **Figures S1** to **S3** illustrate relevant photophysical properties of the lamps.

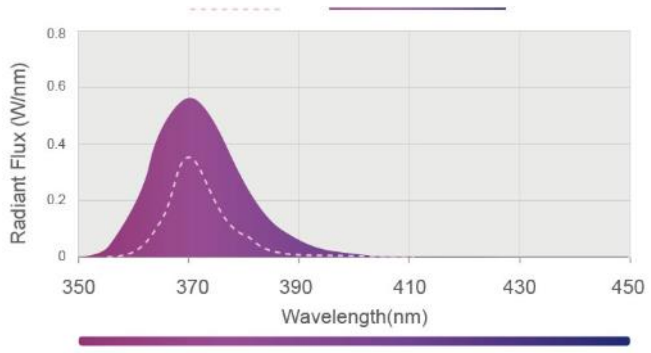

Figure S2. Emission spectrum of the Kessil® Lamps.

Power Consumption (AC)	370nm Gen 2 (max 44W), 370nm (max 43W), 390nm (max 52W), 427nm & 440nm (max 45W), 456nm (max 50W), 467nm (max 44W), 525nm (max 44W)
Input Voltage	100-240 VAC
Operating Temperature	0 - 40°C / 32 - 104°F
Beam Angle	56°
Wavelength Options	370nm, 390nm, 427nm, 440nm, 456nm, 467nm, 525nm
Average Intensity of PR160 series	399mW/cm2 (measured from 1 cm distance)
Dimensions (H x D)	4.49" x 2.48" / 11.4cm x 6.3cm

Figure S3. Technical specifications of the Kessil® Lamps.

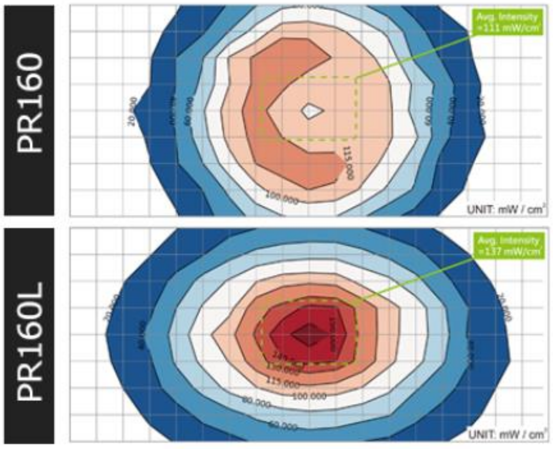

Figure S4. Cross section of illumination area @6cm distance.

Commercial grade reagents and solvents were purchased from Acros Organics, Alfa Aesar, Fluorochem, Sigma-Aldrich and TCI Chemicals, and used as received without further purification. THF and toluene were purified by passing through a Pure Solv^{TM} column drying system from Innovative Technology, Inc.

2a is commercially available reagent and it was used without further purification.

Analytical TLC was performed using pre-coated aluminum-backed plates (Merck TLC Silicagel 60 F_{254}) and visualized by ultraviolet irradiation. Chromatographic purification of products was accomplished by flash chromatography using silica gel (Merck Geduran® Si 60) or aluminum oxide (activated, basic, Brockmann I).

A custom-made photoreactor setup was used for the photocatalytic reactions (See Figure S5). For photocatalytic reactions, crimp vial Headspace 10 mL were employed (Fisher scientific Cat. No. 10681033). The vial is placed inside the fitted well in which irradiation takes place at the desired wavelengths (365, 385, 420, 450 or 540 nm) using 380 mW single LEDs. The distance between the LED and the vial is 3 mm. Reaction temperature is kept at 20-25 ºC using a recirculating chiller.

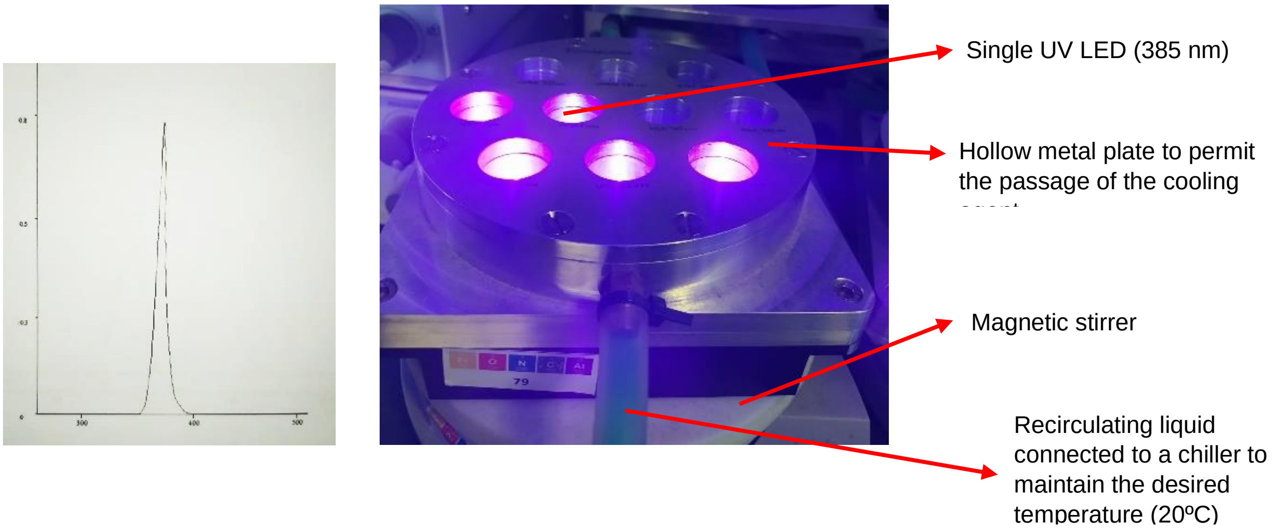

Figure S5. Emission spectrum of the LED used and experimental setup employed during photocatalytic reactions.

UV-Vis measurements were carried out on an Agilent 8453 UV-Visible Spectroscopy System controlled by UV-Visible ChemStation Software. Emission intensities were recorded using a JASCO Spectrofluorometer FP-8600 equipped with a TC-815 Peltier thermostated single cell holder (water-cooled) controlled by Spectra Manager Version 2.10.01. Time resolved emission spectra were recorded using an Edimburg Instruments FS5 Spectrofluorometer, and a 450 nm EPL laser.

2. Synthesis and characterization of alcohols 1

2.1 General procedure A for the synthesis of alcohols (1a-1j)

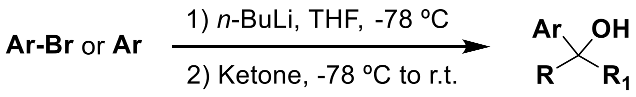

(1a - 1k)

Following procedure of Knowles *et al*[1] to a flame-dried round bottom flask provided with a magnetic stirrer under nitrogen atmosphere, the corresponding aryl bromide or arene (1.0 equiv.) was added and dissolved in anhydrous THF (0.5 M). The solution was cooled to -78 ºC, then *n*-BuLi was added (2.5 M in hexanes, 1.1 equiv.) and the reaction was stirred at this temperature for 30 min. Then the ketone (1.0 equiv.) was slowly added into the solution. The reaction was allowed to warm up to room temperature and the consumption of ketone was monitored by TLC (usually 16h). Then, NH_4Cl sat. solution was added and the organic layers were extracted three times with EtOAc, dried over $MgSO_4$, filtered and concentrated under reduce pressure. The crude product was purified by flash column chromatography on silica gel (Cy /EtOAc) to provide the corresponding pure alcohols (**1a-1k**).

3-(4-Methoxyphenyl)tetrahydrofuran-3-ol (1a)

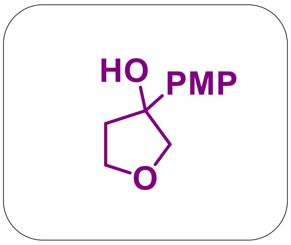

Following the General Procedure A with dihydrofuran-3(2*H*)-one and 4-bromoanisole on 5.0 mmol scale, product **1a** was obtained as a white solid in 51% Yield (492.7 mg) after purification by flash column chromatography (silica, gradient from 20% to 60% of EtOAc in cyclohexane).

R_f = 0.25 (3:1 cyclohexane:EtOAc).

^{1}H NMR (300 MHz, $CDCl_3$): δ 7.41 (d, *J* = 9.0 Hz, 2H), 6.91 (d, *J* = 8.9 Hz, 2H), 4.26 – 4.07 (m, 2H), 3.98 (dd, *J* = 9.3, 1.4 Hz, 1H), 3.87 (d, *J* = 9.3 Hz, 1H), 3.82 (s, 3H), 2.46 – 2.35 (m, 1H), 2.31 – 2.16 (m, 1H).

These data are consistent with those previously reported in the literature for this compound.[2]

3-(4-methoxyphenyl) oxetan-3-ol (1b)

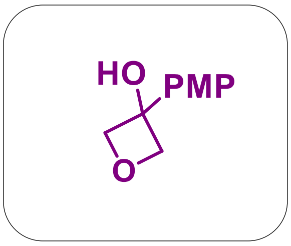

Following the General Procedure A with oxetan-3-one and 4-bromoanisole on 5.0 mmol scale, product **1b** was obtained as a white solid in 82% Yield (740.1 mg) after purification by flash column chromatography (silica, gradient from 20% to 60% of EtOAc in cyclohexane).

R*f* = 0.25 (3:1 cyclohexane:EtOAc).

^{1}H NMR (300 MHz, $CDCl_3$) δ 7.49 (d, *J* = 8.9 Hz, 2H), 6.95 (d, *J* = 8.9 Hz, 2H), 4.93 -4.90 (m, 4H), 3.83 (s, 3H).

These data are consistent with those previously reported in the literature for this compound.[2]

tert-Butyl 3-hydroxy-3-(4-methoxyphenyl)piperidine-1-carboxylate (1c)

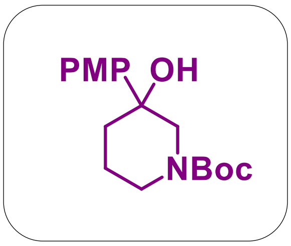

Following the General Procedure A with tert-butyl 3-oxopiperidine-1-carboxylate and 4-bromoanisole on 5.0 mmol scale, product **1c** was obtained as a colourless oil in 17% Yield (255.0 mg) after purification by flash column chromatography (silica, gradient from 20% to 60% of EtOAc in cyclohexane).

R*f* = 0.17 (5:1 cyclohexane:EtOAc).

^{1}H NMR (300 MHz, C_6D_6) δ 7.35 (d, *J* = 9.0 Hz, 2H), 6.81 (d, *J* = 9.0 Hz, 2H), 4.29 – 3.99 (m, 2H), 3.34 (s, 3H), 2.99 (d, *J* = 13.7 Hz, 1H), 2.64 – 2.47 (m, 1H), 1.88 – 1.62 (m, 1H), 1.59 (d, *J* = 4.2 Hz, 1H), 1.46 (s, 9H), 1.19 – 1.11 (m, 2H).

These data are consistent with those previously reported in the literature for this compound.[1]

1-(4-methoxyphenyl)cyclohexan-1-ol (1d)

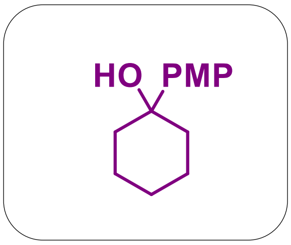

Following the general procedure A with cyclohexanone and 4-bromoanisole on 5.0 mmol scale, product **1d** was obtained as a colourless oil in 75% Yield (769.0 mg) after purification by flash column chromatography (silica, gradient from 5% to 20% of EtOAc in cyclohexane).

R_f = 0.18 (4:1 cyclohexane:EtOAc).

^{1}H NMR (300 MHz, C_6D_6) δ 7.34 (d, *J* = 9.0 Hz, 2H), 6.84 (d, *J* = 8.9 Hz, 2H), 3.35 (s, 3H), 1.81 – 1.56 (m, 7H), 1.51 – 1.41 (m, 2H), 1.19 – 1.04 (m, 1H).

These data are consistent with those previously reported in the literature for this compound.[3]

1-(4-methoxyphenyl)cyclopentadecan-1-ol (1e)

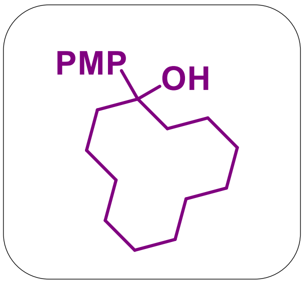

Following general procedure A with cyclopentadecanone and 4-bromoanisole on a 5.0 mmol scale **1e** was obtained as a white solid in 53% Yield (884.0 mg) after purification by column chromatography (silica, gradient from 5% to 15% of EtOAc in cyclohexane) in 53% yield (884 mg).

R_f = 0.53 (4:1 cyclohexane:EtOAc).

^{1}H NMR (300 MHz, C_6D_6) δ 7.43 – 7.34 (m, 2H), 6.91 – 6.81 (m, 2H), 3.38 (s, 3H), 1.90 – 1.63 (m, 4H), 1.26 (d, *J* = 31.1 Hz, 18H), 1.08 (bs, 1H).

These data are consistent with those previously reported in the literature for this compound.[6]

3-(Furan-2-yl)tetrahydro-2H-pyran-3-ol (1f)

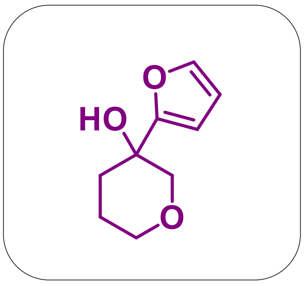

Following the general procedure A with dihydro-2*H*-pyran-3(4*H*)-one and furan on 2.0 mmol scale, **1f** was obtained as a yellowish oil in 21% Yield (70.0 mg) after purification by flash column chromatography (silica, gradient from 10% to 40% of EtOAc in cyclohexane).

R_f = 0.20 (2:1 cyclohexane:EtOAc).

^{1}H NMR (300 MHz, CD_2Cl_2) δ 7.39 (dd, *J* = 1.8, 0.9 Hz, 1H), 6.36 (dd, *J* = 3.3, 1.8 Hz, 1H), 6.30 (dd, *J* = 3.3, 0.9 Hz, 1H), 3.85 – 3.74 (m, 1H), 3.73 – 3.68 (m, 2H), 3.54 – 3.45 (m, 1H), 2.55 (brs, 1H), 2.19 – 2.02 (m, 1H), 2.02 – 1.84 (m, 2H), 1.64 – 1.45 (m, 1H).

These data are consistent with those previously reported in the literature for this compound.[3]

3-(Benzofuran-2-yl)tetrahydro-2H-pyran-3-ol (1g)

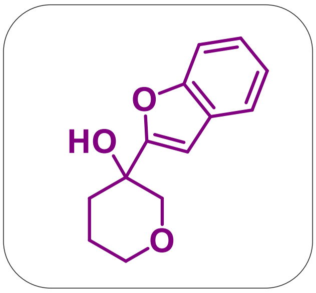

Following the general procedure A with dihydro-2*H*-pyran-3(4*H*)-one and benzofuran on 2.0 mmol scale, **1g** was obtained as a yellowish oil in 58% Yield (252 mg) after purification by flash column chromatography (silica, gradient from 20% to 60% of EtOAc in cyclohexane).

R*f* = 0.23 (2:1 cyclohexane:EtOAc).

^{1}H NMR (300 MHz, $CDCl_3$) δ 7.55 (d, J = 7.3 Hz, 1H), 7.46 (d, J = 7.3 Hz, 1H), 7.35 – 7.10 (m, 2H), 6.73 (d, J = 1.1 Hz, 1H), 3.96 – 3.86 (m, 3H), 3.69 – 3.50 (m, 1H), 2.34 – 2.18 (m, 1H), 2.14 – 1.98 (m, 2H), 1.74 – 1.56 (m, 1H).

These data are consistent with those previously reported in the literature for this compound.[3]

3-(Phenanthren-9-yl)tetrahydro-2H-pyran-3-ol (1h)

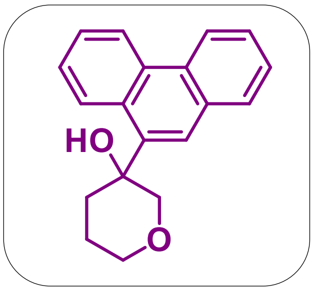

Following the general procedure A with dihydro-2*H*-pyran-3(4*H*)-one and 9-bromophenanthrene on 5.0 mmol scale, **1h** was obtained as a white solid in 30% Yield (416.0 mg) after purification by flash column chromatography (silica, gradient from 5% to 15% of EtOAc in cyclohexane).

R*f* = 0.25 (2:1 cyclohexane:EtOAc).

^{1}H NMR (300 MHz, $CDCl_3$): δ 8.98 (d, J = 7.8 Hz, 1H), 8.76 (d, J = 8.4 Hz, 1H), 8.66 (d, J = 8.2 Hz, 1H), 7.88 (d, J = 7.7 Hz, 1H), 7.80 (s, 1H), 7.74 – 7.53 (m, 4H), 4.20 (d, J = 11.4 Hz, 1H), 4.07 (d, J = 11.4 Hz, 1H), 4.04 – 3.93 (m, 1H), 3.66 (td, J = 11.1, 10.5, 3.2 Hz, 1H), 3.02 (s, 1H), 2.52 – 2.21 (m, 1H), 2.22 – 2.00 (m, 1H), 1.73 – 1.51 (m, 1H).

These data are consistent with those previously reported in the literature for this compound.[3]

1-(4-Methoxyphenyl)-2-phenylcyclohexan-1-ol (1i)

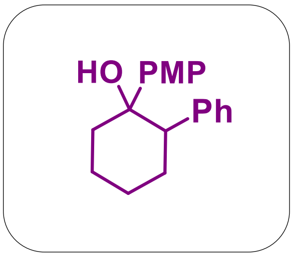

Following general procedure A on with with 2-phenylcyclohexan-1-one and 4-bromoanisole on a 5.0 mmol scale, **1i** was obtained as a white solid in 40%

Yield (564.0 mg) after purification by column chromatography (silica, gradient from 7% to 20% of EtOAc in cyclohexane).

R*f* = 0.62 (4:1 cyclohexane:EtOAc).

^{1}H NMR (300 MHz, CDCl$_3$): δ 7.17 – 7.06 (m, 5H), 7.00 – 6.89 (m, 2H), 6.74 (d, *J* = 8.8 Hz, 2H), 3.75 (s, 3H), 3.02 (dd, *J* = 12.8, 3.6 Hz, 1H), 2.25 (qd, *J* = 13.1, 3.4 Hz, 1H), 2.08 – 1.73 (m, 7H), 1.54 (dtt, *J* = 16.0, 7.3, 3.8 Hz, 1H).

These data are consistent with those previously reported in the literature for this compound.[6]

1-(4-methoxyphenyl)-2-methylcyclohexan-1-ol (1j)

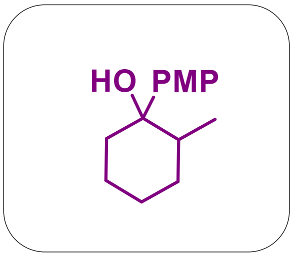

Following general procedure A with 2-methylcyclohexan-1-one and 4-bromoanisole on a 5.0 mmol scale, **1j** was obtained as a white solid in 57% Yield (623.0 mg) after purification by column chromatography (silica, gradient from 5% to 10% of EtOAc in cyclohexane).

R*f* = 0.59 (4:1 cyclohexane:EtOAc).

^{1}H NMR (300 MHz, CD$_2$Cl$_2$): δ 7.36 (d, *J* = 8.8 Hz, 2H), 6.87 (d, *J* = 8.8 Hz, 2H), 3.81 (s,3H), 1.98 – 1.84 (m, 1H), 1.83 – 1.74 (m, 1H), 1.71 - 1.69 (m, 2H), 1.66 – 1.52 (m, 4H), 1.52 – 1.35 (m, 2H), 0.63 (d, *J* = 6.8 Hz, 3H).

These data are consistent with those previously reported in the literature for this compound.[4]

2.2 Procedure for the synthesis of 1-(4-methoxyphenyl)cyclopropan-1-ol (1k)

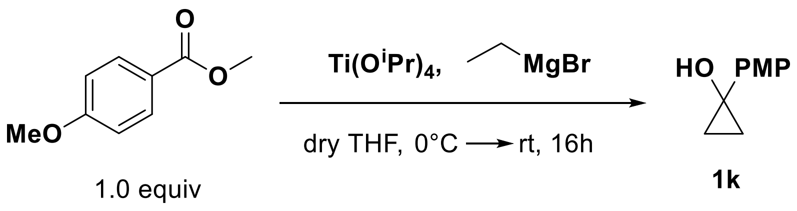

Following a procedure of Morril *et al*[5] to a flame-dried round bottom flask provided with a magnetic stirrer under nitrogen atmosphere, methyl 4-methoxybenzoate

(0.83 g, 5.0 mmol, 1.0 equiv.) and $Ti(OiPr)_4$ (2.13 mL, 7.0 mmol, 1.4 equiv.), were added and dissolved in dry THF (0.16 M). The mixture was cooled to 0 °C and a solution of ethylmagnesium bromide (4.65 mL, 3.0 M, 14.0 mmol, 2.8 equiv.) was added dropwise. Then, the reaction was allowed to warm to 25 °C and stirred overnight. After completion water was added and the resulting precipitate was filtered under vacuum through celite and washed with EtOAc. The layers of the filtrate were separated, and the aqueous layer was further extracted with EtOAc. The combined organics were dried (MgSO4), filtered, and concentrated under reduced pressure. The crude product was isolated as a colourless oil after purification by flash column chromatography (silica, gradient from 13% to 33% of EtOAc in cyclohexane) in 26% Yield (215.2 mg).
R_f = 0.41 (3:1 cyclohexane:EtOAc).

^{1}H NMR (300 MHz, $CDCl_3$): δ 7.35 – 7.17 (m, 2H), 6.94 – 6.78 (m, 2H), 3.81 (s, 3H), 1.25 – 1.15 (m, 1H), 1.00 – 0.83 (m, 2H).
These data are consistent with those previously reported in the literature for this compound.[5]

3. Synthesis and characterization of Michael acceptors 2

3.1 Synthesis and characterization of α,β-unsaturated alkenes 2a-2g

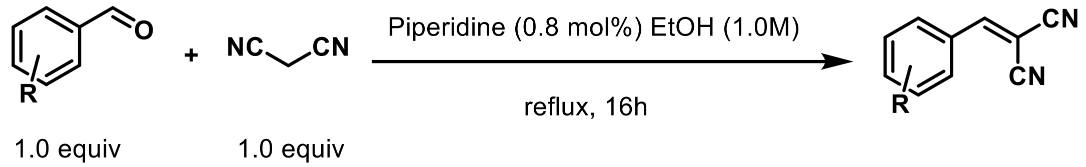

General procedure B: Following the reported procedure[6] to a solution of malononitrile (330 mg, 5 mmol, 1.0 equiv.) and substituted benzaldehyde (4.81 mmol, 1 equiv.) in EtOH (5 mL), piperidine (2 drops) was added. The reaction mixture was stirred at refluxed for 16 h. Then, the reaction was allowed to warm to 25 °C, diluted with a saturated solution of NH_4Cl (5 mL) and extracted with CH_2Cl_2 three times, dried over MgSO4, filtered and concentrated under reduce

pressure. The crude product was purified by flash column chromatography on silica gel (Cy /EtOAc) to provide the corresponding Michael acceptor (**2b-2g**).

2-(4-Cyanobenzylidene)malononitrile (2b)

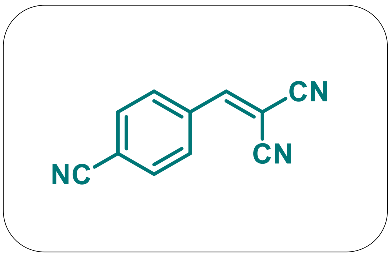

Following general procedure B, with 4-formylbenzonitrile, **2b** was obtained as a white solid after purification by column chromatography (silica, gradient from 10% to 65% of EtOAc in cyclohexane) in a 84% Yield (1.024 g)

R*f* = 0.50 (4:1 cyclohexane:EtOAc).

^{1}H NMR (300 MHz, CDCl$_3$): δ 8.03 – 7.97 (m, 2H), 7.86 – 7.82 (m, 2H), 7.81 (s, 1H).

These data are consistent with those previously reported in the literature for this compound.[6]

2-(3,5-bis(Trifluoromethyl)benzylidene)malononitrile (2c)

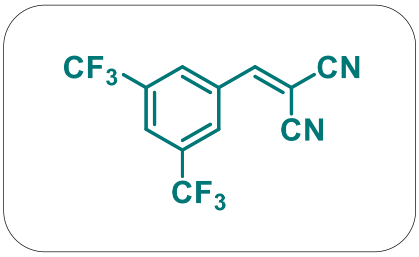

Following general procedure B, with 3, 5-bis(trifluoromethyl)benzaldehyde, **2c** was obtained as a yellow solid after purification by column chromatography (silica, gradient from 7% to 35% of EtOAc in cyclohexane) in 17% yield (250 mg).

R*f* = 0.59 (4:1 cyclohexane:EtOAc).

^{1}H NMR (300 MHz, CDCl$_3$): δ 8.36 – 8.29 (m, 2H), 8.15 – 8.07 (m, 1H), 7.89 (s, 1H).

These data are consistent with those previously reported in the literature for this compound.[6]

2-(4-Chlorobenzylidene)malononitrile (2d)

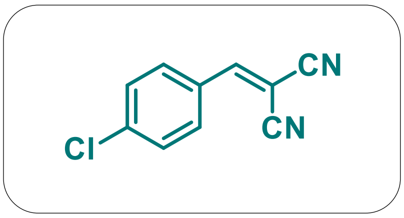

Following general procedure B, with 4-chlorobenzaldehyde, **2d** was obtained as a white solid after purification by column chromatography (silica, gradient from 9% to

40% of EtOAc in cyclohexane) in 12% yield (112 mg).

R*f* = 0.49 (4:1 cyclohexane:EtOAc).

^{1}H NMR (300 MHz, $CDCl_3$): δ 7.86 (d, *J* = 8.4 Hz, 1H), 7.73 (s, 1H), 7.52 (d, *J* = 8.6 Hz, 1H).

These data are consistent with those previously reported in the literature for this compound.[7]

2-(4-Fluorobenzylidene)malononitrile (2e)

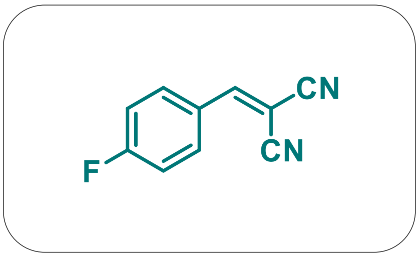

Following general procedure B, 4-fluorobenzaldehyde **2e** was obtained as a yellow solid after purification by column chromatography (silica, gradient from 2% to 20% of EtOAc in cyclohexane) in 38% yield (325 mg).

R*f* = 0.66 (2:1 cyclohexane:EtOAc).

^{1}H NMR (300 MHz, $CDCl_3$): δ 7.99 – 7.93 (m, 2H), 7.74 (s, 1H), 7.27 – 7.20 (m, 2H).

These data are consistent with those previously reported in the literature for this compound.[8]

2-(4-(*tert*-Butyl)benzylidene)malononitrile (2f)

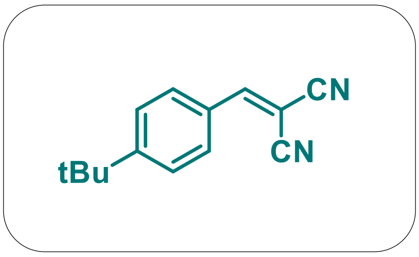

Following general procedure B, with 4-(tert-butyl) benzaldehyde, **2f** was obtained as a white solid after purification by column chromatography (silica, gradient from 5% to 15% of EtOAc in cyclohexane) in a 65% yield (688 mg).

R*f* = 0.67 (4:1 cyclohexane:EtOAc).

^{1}H NMR (300 MHz, $CDCl_3$): δ 7.91 – 7.83 (m, 2H), 7.74 (s, 1H), 7.56 (dd, *J* = 8.8, 2.1 Hz, 2H), 1.35 (s, 9H).

These data are consistent with those previously reported in the literature for this compound.[9]

2-(3-Methylbenzylidene)malononitrile (2g)

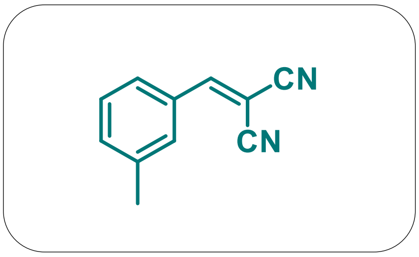

Following general procedure E, with 3-methylbenzaldehyde, **2g** was obtained as a white solid after purification by column chromatography (silica, gradient from 10% to 70% of EtOAc in cyclohexane) in 28% yield (235 mg).

R*f* = 0.45 (4:1 cyclohexane:EtOAc).

^{1}H NMR (300 MHz, $CDCl_3$): δ 7.76 – 7.71 (m, 2H), 7.69 (s, 1H), 7.46 – 7.40 (m, 2H), 2.43 (s, 3H).

These data are consistent with those previously reported in the literature for this compound.[6]

3.2 Synthesis and characterization of α,β-unsaturated alkenes 2h and 2i

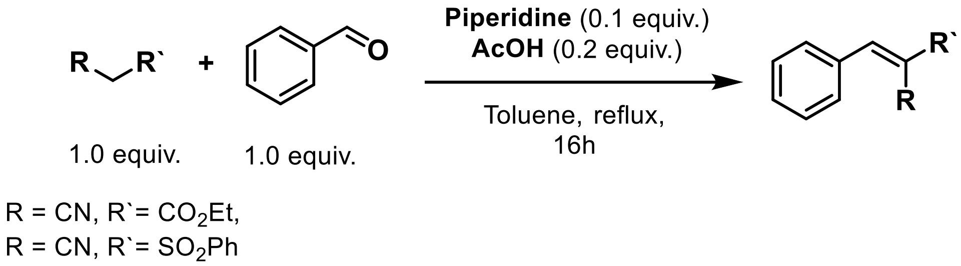

General procedure C: Following a slightly modified procedure of Budynina *et al.*[10] to a solution of benzaldehyde (4.7 mmol, 1.0 equiv.) in toluene (1.3 M) the corresponding nucleophile (malonate) (4.7 mmol, 1.0 equiv.), piperidine (0.1 equiv.) and acetic acid (0.2 equiv.) were added. The reaction was heated under reflux with 10 mL Dean-Stark trap overnight. Reaction mixture was cooled to ambient temperature, diluted with a saturated solution of NH_4Cl (5 mL) and extracted with Et_2O three times. The combined organic layers were dried over Na_2SO_4, filtered, concentrated, and purified by column chromatography to give the corresponding α,β-unsaturated alkenes **2h** and **2i.**

Ethyl (*Z*)-2-cyano-3-phenylacrylate (2h)

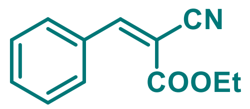

Following general procedure C, with ethyl 2-cyanoacetate, on a 4.7 mmol scale **2h** was isolated as a yellowish oil after purification by column chromatography (silica, gradient from 10% to 30% of EtOAc in cyclohexane) in a 78% yield (740 mg).

R_f = 0.53 (4:1 cyclohexane:EtOAc).

^{1}H NMR (300 MHz, $CDCl_3$): δ 8.25 (s, 1H), 7.99 (ddd, *J* = 8.4, 1.6, 0.6 Hz, 2H), 7.59 – 7.46 (m, 3H), 4.39 (q, *J* = 7.1 Hz, 2H), 1.40 (t, *J* = 7.1 Hz, 3H).

These data are consistent with those previously reported in the literature for this compound.[6]

3-Phenyl-2-(phenylsulfonyl)acrylonitrile (2i)

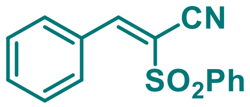

Following general procedure C, with 2-(phenylsulfonyl)acetonitrile, **2i** was obtained as a red solid after purification by column chromatography (silica, gradient from 5% to 30% of EtOAc in cyclohexane) in 47% yield (313 mg).

R_f =0.38 (4:1 cyclohexane:EtOAc).

^{1}H NMR (300 MHz, $CDCl_3$): δ 8.24 (s, 1H), 8.06 – 7.98 (m, 2H), 7.95 – 7.87 (m, 2H), 7.74 – 7.66 (m, 1H), 7.65 – 7.54 (m, 3H), 7.48 (tt, *J* = 6.6, 1.7 Hz, 2H).

These data are consistent with those previously reported in the literature for this compound.[6]

4. Limitations of the reaction

The reaction presents several scope limitations (Figure S6). In the cycloalkanol side an electron rich (hetero)arene easily oxidizable is required for the reaction to take place. Therefore, with a phenyl substituent no EDA formation is observed (see Figure S8). However, with the m-Br C_6H_4, although a bathochromic shift on

the absorption band is observed indicating an interaction between the two molecules in the ground state, the electron deficient character of the arene does not allow the SET to take place and no reaction is observed (Figure S9).

On the alkene side one CN group is always required. As shown in Figure S6, alkenes bearing either different EWG or electron rich arenes in the benzocyclidine moiety were studied with unsuccessful results. UV-vis absorption analysis revealed that no EDA is formed in the ground state, what explains the lack of reactivity observed before (see Figures S10 and S11).

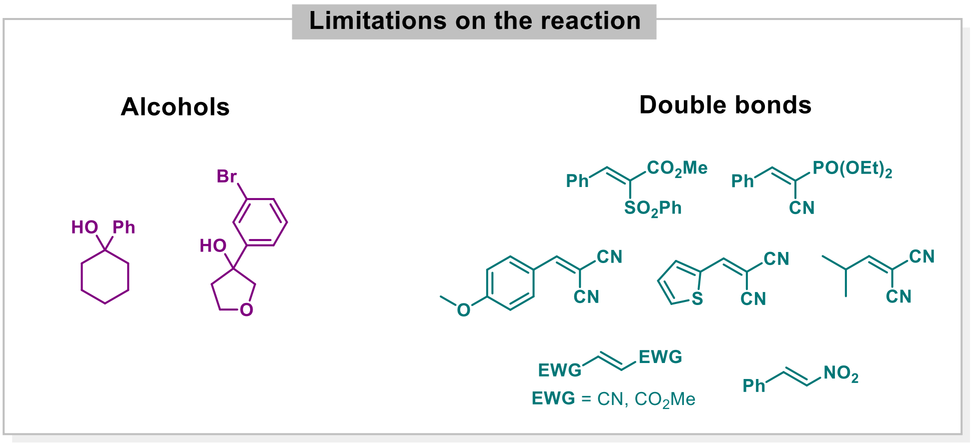

Figure S6. Limitations on scope of the reaction

5. UV-Vis absorption spectra

a) Study of the EDA complex formation:

Measurements were carried out using CH_3CN as solvent in 0.1M concentration.

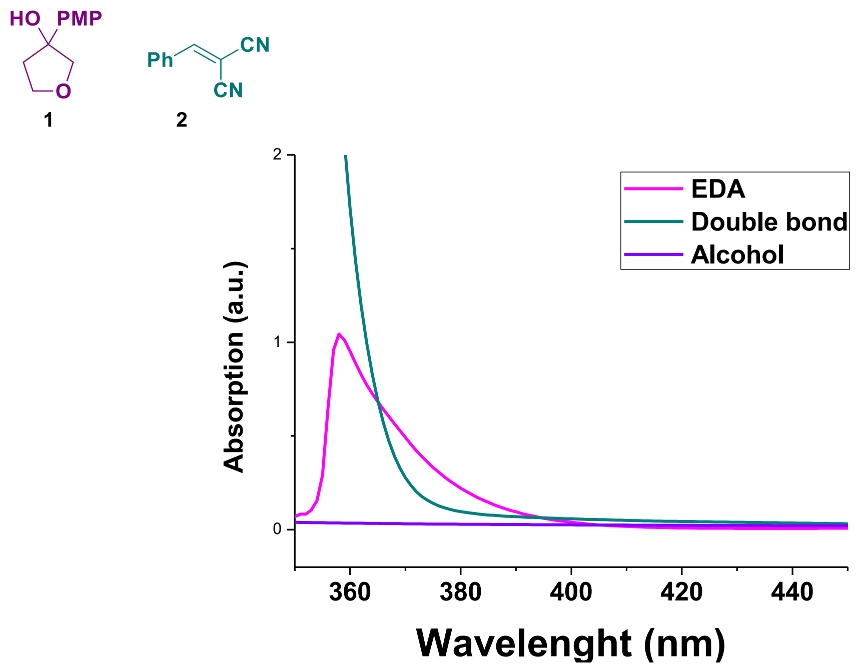

Figure S7: UV-Vis spectra of compounds **1** (purple line), **2** (green line) and EDA complex minus the absorption spectrum of **1** minus the absorption spectrum of **2** (pink line).

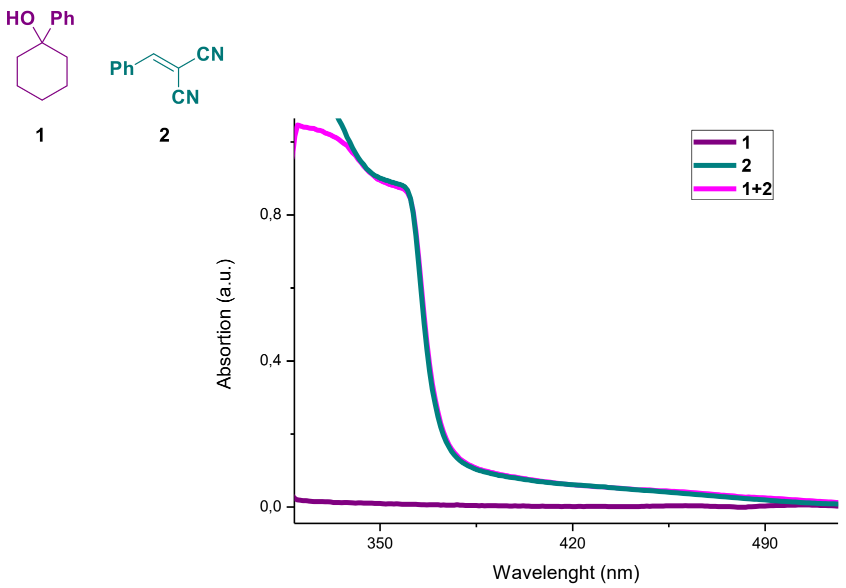

Figure S8: UV-Vis spectra of cycloalkanol **1** (purple line), alkene **2** (green line) and **1** + **2** (pink line)

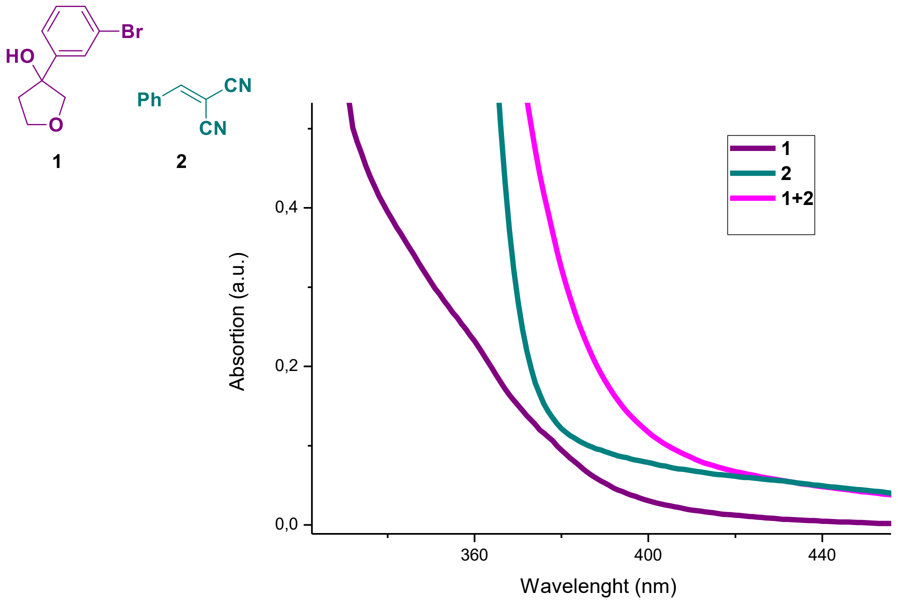

Figure S9: UV-Vis spectra of cycloalkanol **1** (purple line), alkene **2** (green line) and **1** + **2** (pink line).

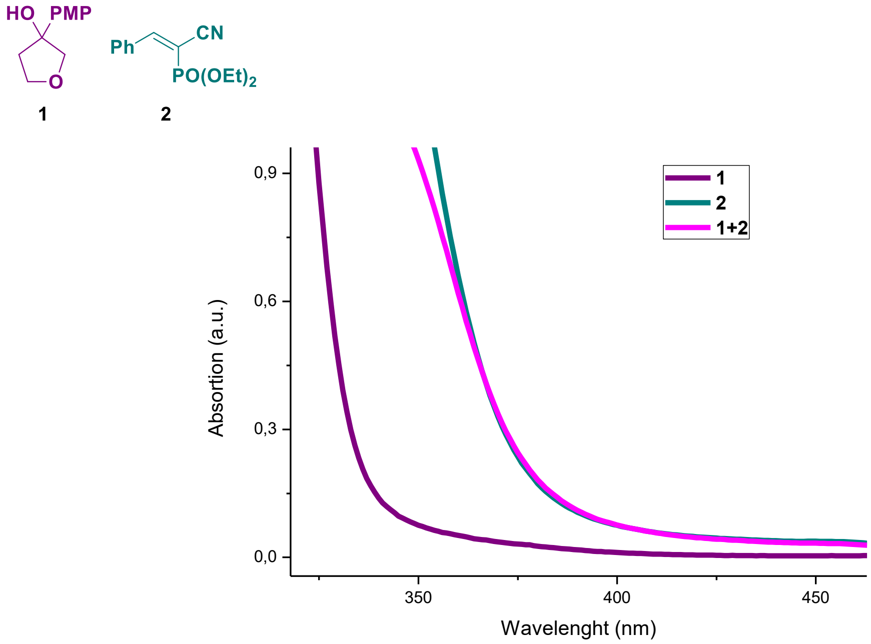

Figure S10: UV-Vis spectra of cycloalkanol **1** (purple line), alkene **2** (green line) and **1** + **2** (pink line).

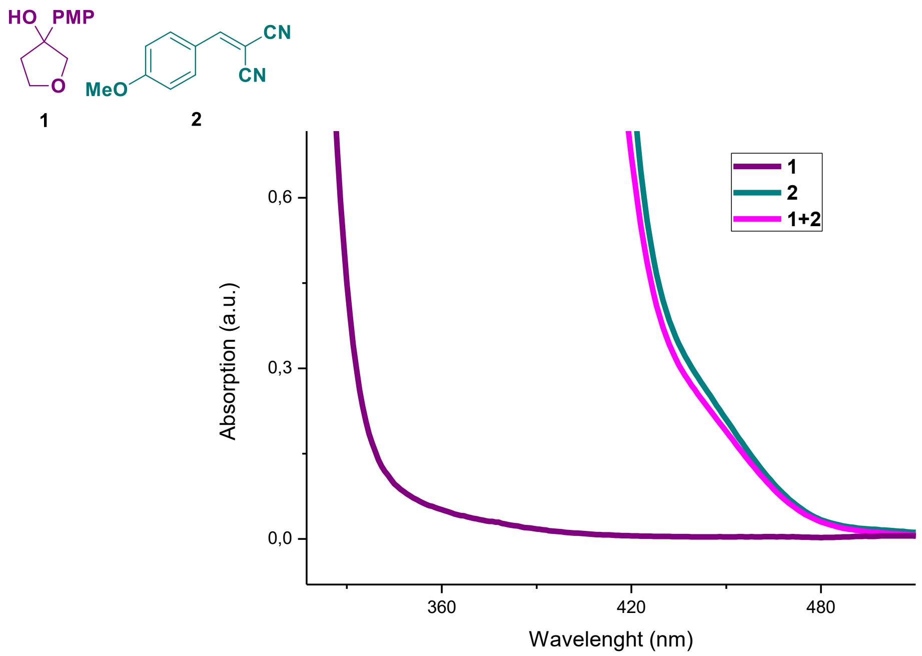

Figure S11: UV-Vis spectra of cycloalkanol **1** (purple line), alkene **2** (green line) and **1** + **2** (pink line)

b) Study of the effect of 2,6-lutidine in the EDA complex absorption spectrum.

No changes were observed in the absorption band of the different EDA complexes studied in the presence of 25 mol% of lutidine.

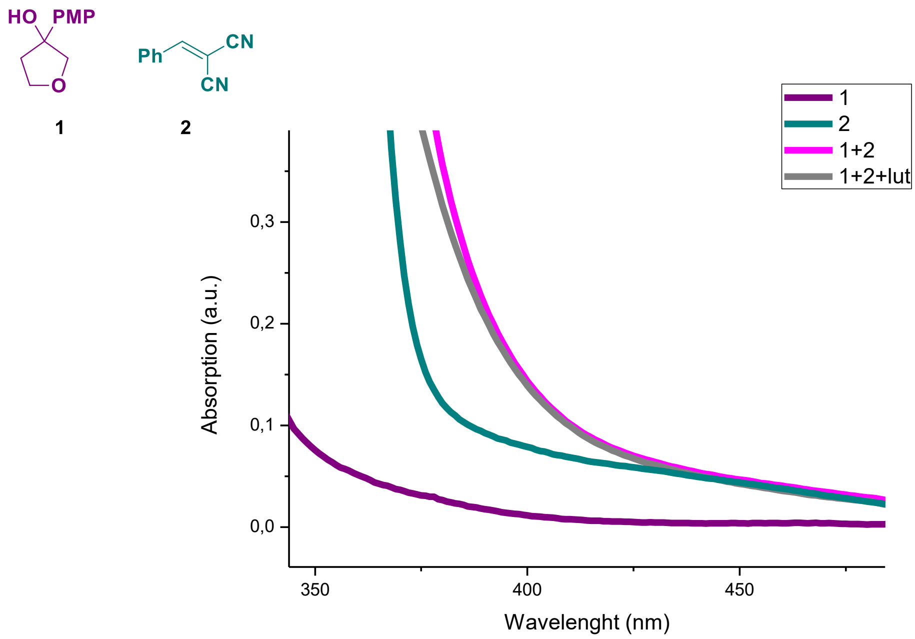

Figure S12: UV-Vis spectra of cycloalkanol **1** (purple line), alkene **2** (green line), EDA complex **1** + **2** (pink line) and **1** + **2** with 25 mol% 2,6-lutidine (grey line)

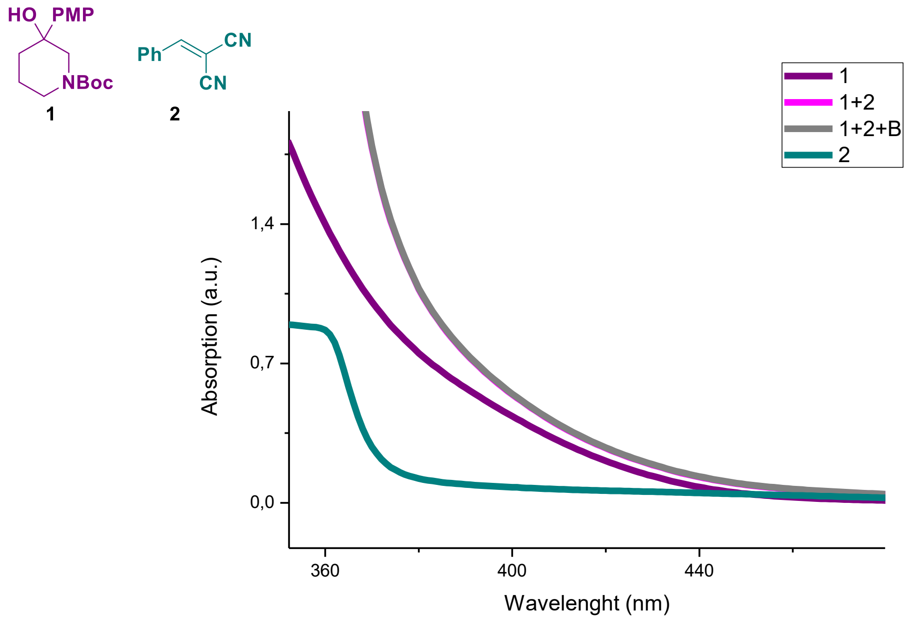

Figure S13: UV-Vis spectra of cycloalkanol **1** (purpel line), alkene **2** (green line), EDA complex **1** + **2** (pink line) and **1** + **2** with 25 mol% 2,6-lutidine (grey line)

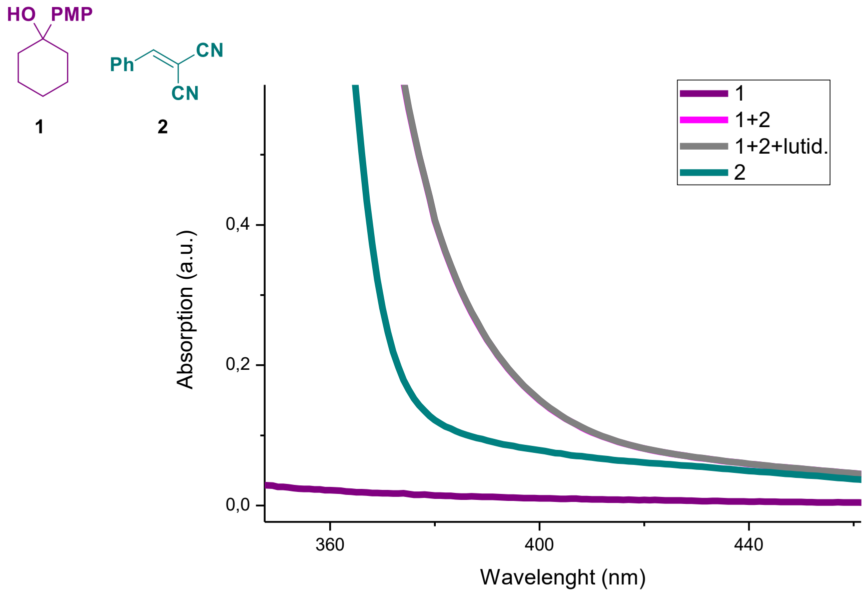

Figure S14: UV-Vis spectra of cycloalkanol **1** (purpel line), alkene **2** (green line), EDA complex **1** + **2** (pink line) and **1** + **2** with 25 mol% 2,6-lutidine (grey line)

6 Mechanistic studies on the photocatalytic reaction

6.1 Stoichiometry of the EDA complex (Job plot).

The stoichiometry of the EDA complexes was calculated using the Job plot method (Figure S15). The Job plot of the EDA complex between **1a** and **2a** was calculated measuring the absorption of CH_3CN solutions at 385 nm with different donor/acceptor ratios with constant concentration (0.1 M) of the two components. The absorbance values were plotted against the molar fraction (%) of **2**. The Job plot analysis of the EDA complex **1a/2a** showed a maximal absorbance at 58% molar fraction of **2a** indicated the 1:1 stoichiometry of the EDA complex in solution.

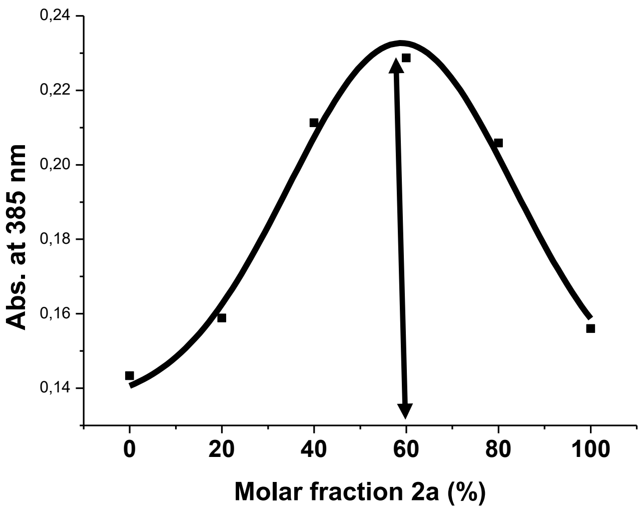

Figure S15: Job plot between the molar fraction of **2a** and the absorbance.

6.2 Determination of the association constant.

The association constant of the EDA complex formed between **1a** and **2a** was determined spectrophotometrically in CH_3CN, employing the Benesi-Hildebrand method[11] (Figure S16). We measured the absorption of solutions with constant concentration of **1a** (0.1 M) adding an excess of **2a** to increase the donor/acceptor ratio. According to the method, a straight line was obtained when the reciprocal

of the absorbance (A) was plotted against the reciprocal of the concentration of the partner in excess.

Data obtained were displayed in Table S1 and Figure S16. The association constant (K_{EDA}) was calculated by dividing the intercept by the slope: 0.1 M-1.

[2a]	1/[2a]	Abs_{EDA}	1/(Abs_{EDA} – A_0)
0.1	10	0,13009	9,08259
0.15	6,66667	0,18899	5,91682
0.2	5	0,24083	4,52797
0.3	3,33333	0,37376	2,82666
0.4	2,5	0,45438	2,30206

Table S1: Table of obtained data from UV-vis absorption spectra for EDA in CH_3CN with **1a** and **2a**.

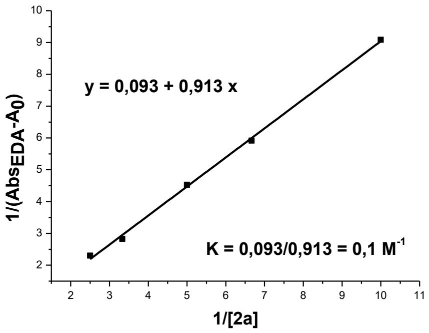

Figure S16: Hildebrand-Benesi plot for the EDA complex **1a**/**2a**.

6.3 ^{1}H-NMR titration experiment.

Six solutions containing the same amount of **1a** (donor) were prepared, with increasing the amount of **2a** (acceptor) and keeping the molarity constant (0.1M in CD_3CN). These samples were measured by ^{1}H-NMR (Figure S17) and the shift of the marked signals were analysed in order to prove the interaction between the two partners.

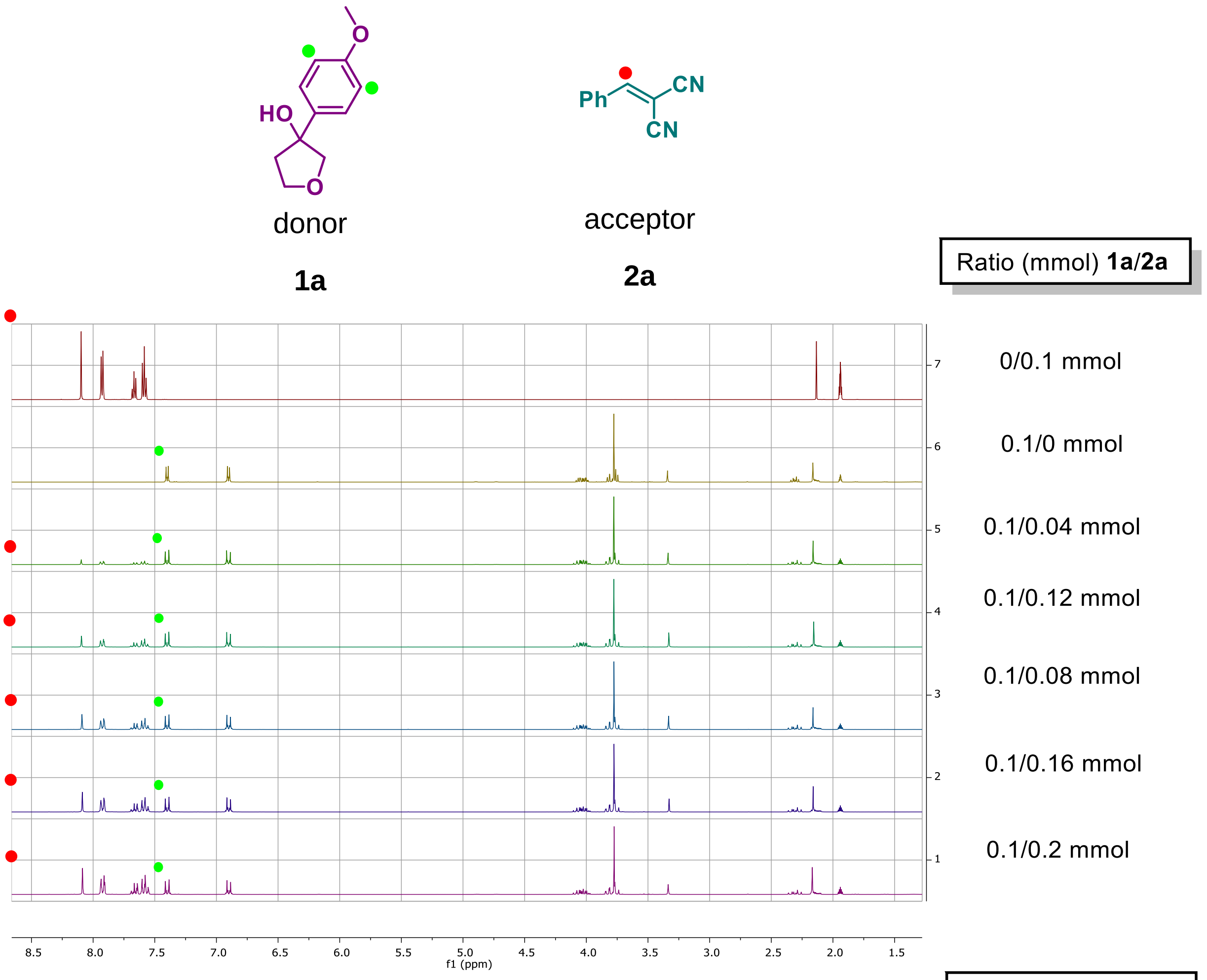

Figure S17: ^{1}H-NMR in CD_3CN in different donor/acceptor ratio (0.1 M) stacked.

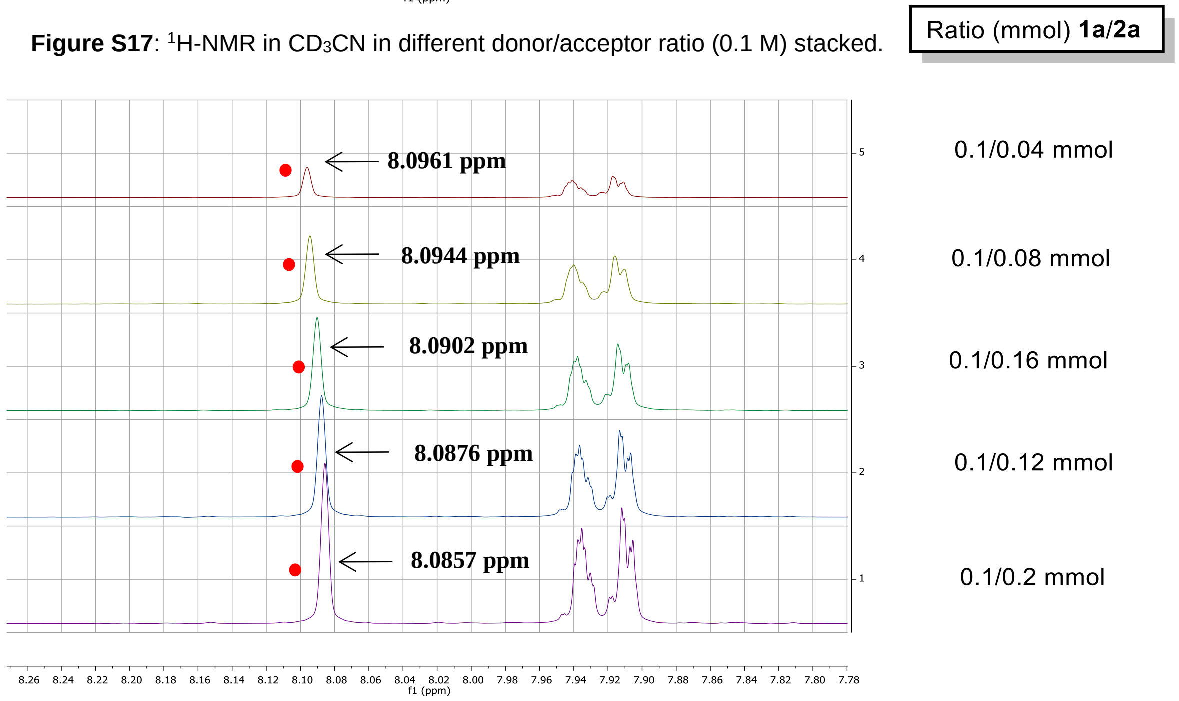

Figure S18: ^{1}H-NMR in CD_3CN in different donor/acceptor ratio (0.1 M) stacked acceptor marked.

The reported stacking of ^{1}H-NMR spectra shows that increasing the amount of **2a** in a constant concentration (0.1 M), a marked upfield shift of the double bond singlet at 8.0857 ppm (red dots) is observed (Figure S18).

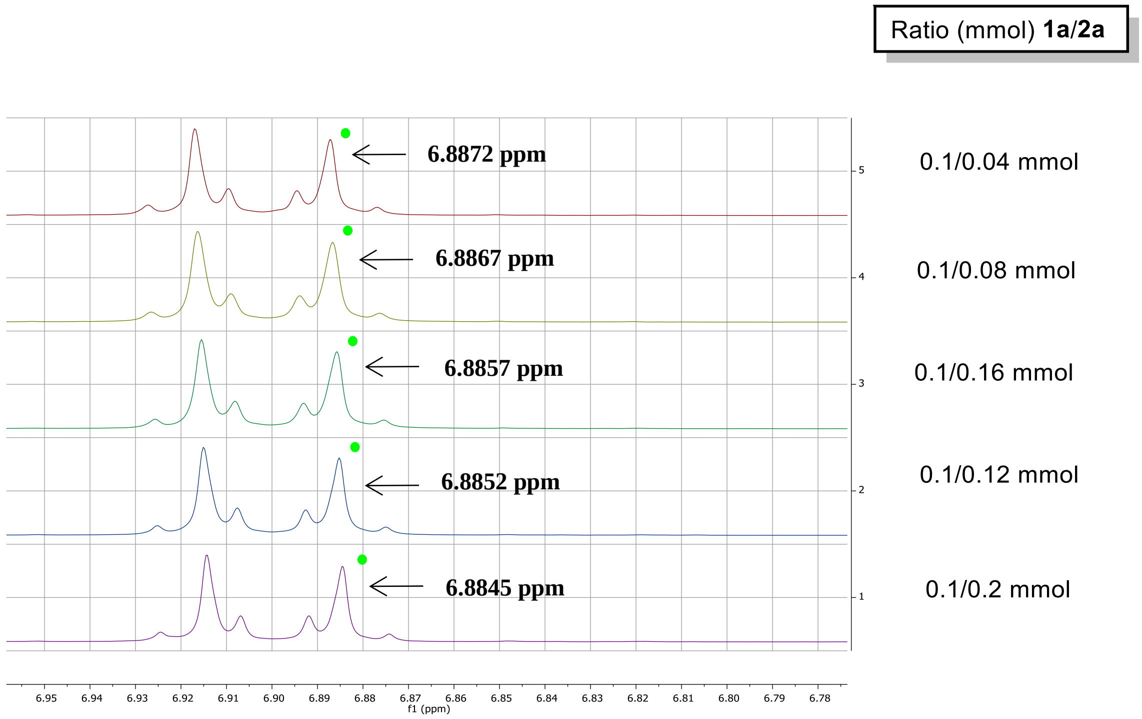

Figure S19: ^{1}H-NMR in CD_3CN in different donor/acceptor ratio (0.1 M) stacked donor marked.

In the same conditions, a downfield shift of the doublet belonging to a *para*-methoxy group at 6.8845 ppm (green dots) of **1a** is detected. This result represent an evidence of an interaction between the alcohol and the Michael acceptor in the EDA complex (Figure S19).

6.4 Deuterium labelling experiments

a) Investigation of the possible D atom abstraction from the solvent

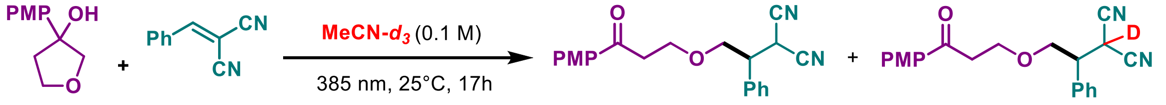

1a 0.1 mmol | **2a** 2.0 equiv | **3a** 60% NMR yield | **3a-*d*** 20% NMR Yield (from **3a**)

A dry vial equipped with a magnetic stir bar was charged with **1a** (0.1 mmol, 1.0 equiv.), **2a** (0.2 mmol, 2.0 equiv.), and 1.0 mL of CH_3CN -d_3 (0.1 M).

Degasification of the reaction mixture was performed via freezepump-thaw cycling (3 x 10 min under vacuum). Then, the reaction mixture was irradiated and stirred in the photoreactor setup under 385 nm LED for 17h. The solvent was evaporated, and 1,3,5-trimethoxybenzene was added as internal standard (IS), and the crude analysed by ^{1}H NMR to determine yield.

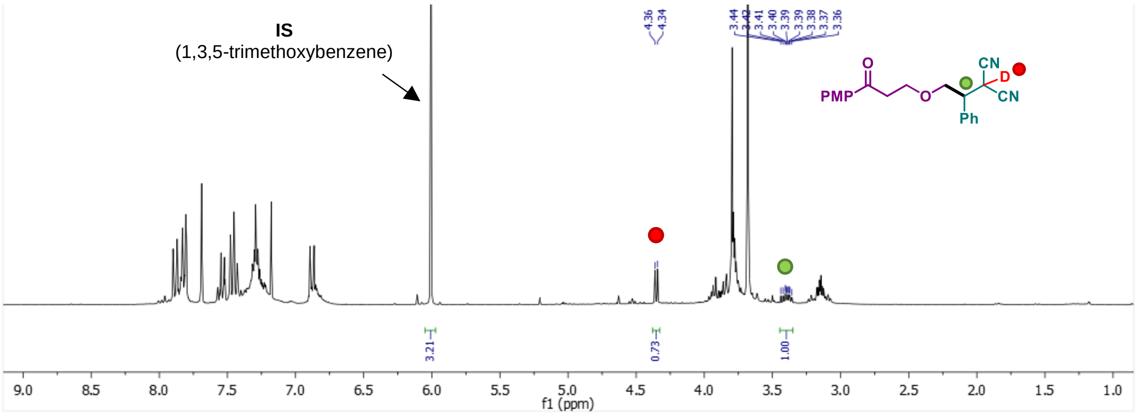

Figure S20: ^{1}H NMR (400 MHz, $CDCl_3$) of crude mixture of **3a** and **3a-*d*** for the reaction run in CH_3CN -*d*$_3$.

In these conditions **3a** was formed in 60% NMR Yield, calculated by integration of a characteristic multiplet reported with a green spot in Figure S20. The deuterium content was determined by comparing the integration of two marked peaks in the spectrum. The red marked doublet at 4.35 ppm shows reduced integral intensity corresponding to 27% D incorporation.

b) <u>Reaction of **2a** with deuterium-labelled alcohol **1a-*d***</u>

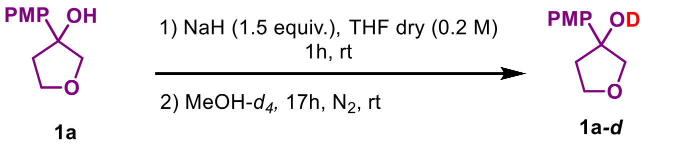

1a 0.3 mmol — **1a-*d*** full integration of deuterium

Alcohol **1a-*d*** was prepared starting from **1a** (0.3 mmol) by dissolving in anhydrous THF (0.2 M) and let stirring for 1h with NaH (1.5 equiv). After that, the solvent was removed under vacuum and the mixture was dissolved in MeOH-*d*$_4$ (3.0 mL) and let stirring overnight under inert atmosphere. The base was removed by filtration and **1a-*d*** was obtained in full conversion as a

colourless solid, and storage under argon. The content of deuterium was determined by the integration of the hydroxyl proton peak in DMSO-d_6. (Figure S21).

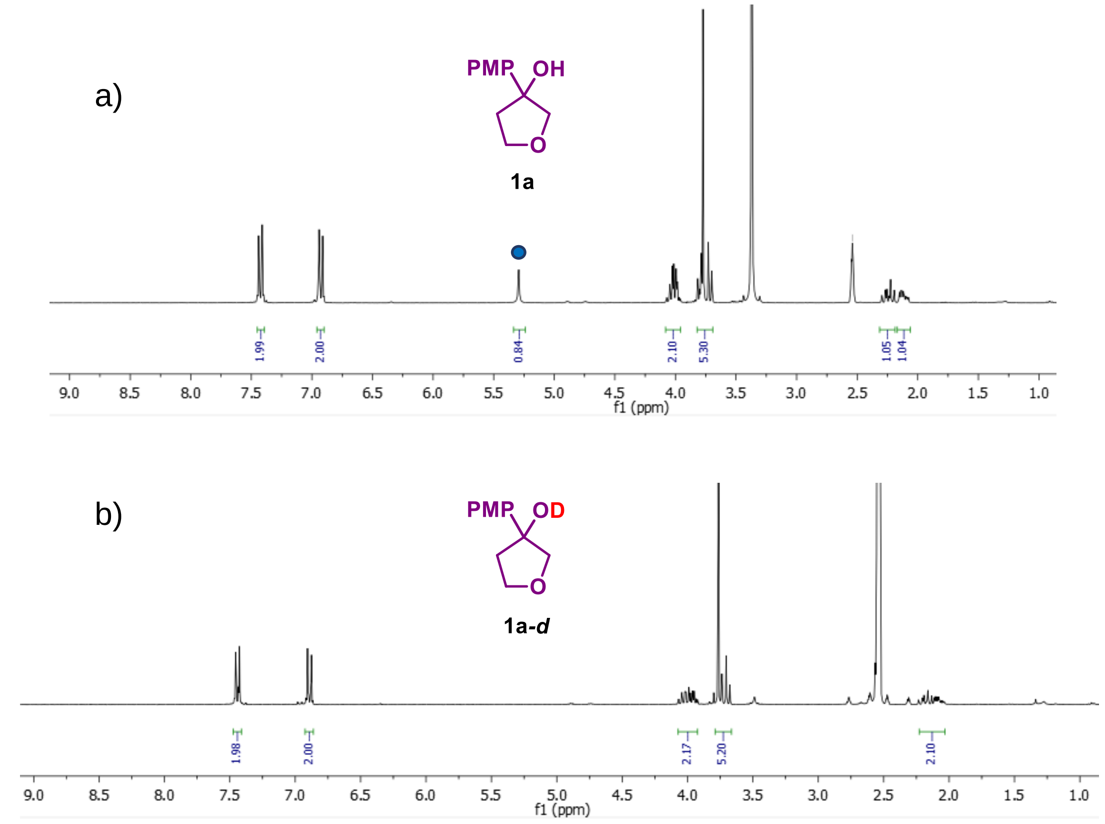

Figure S21. a) ^{1}H NMR (300 MHz, DMSO-d_6) of **1a**; b) ^{1}H NMR (300 MHz, DMSO-d_6) of **1a-*d*.**

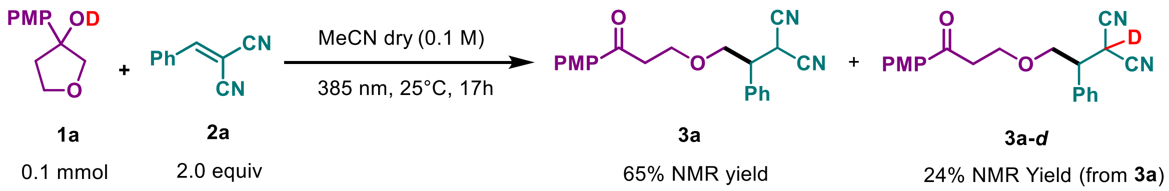

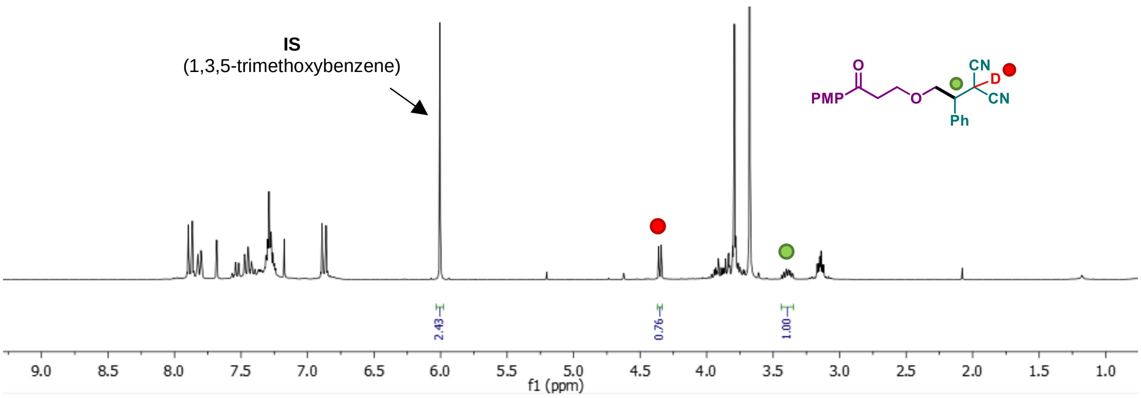

Figure S22: ^{1}H NMR (400 MHz, $CDCl_3$) of crude mixture of **3a** and **3a-*d*** for the reaction run with **1a-*d*.**

The product **3a** was formed in 65% NMR Yield. The deuterium content was determined by comparing the integration of two marked peaks in the spectrum. The red marked doublet at 4.35 ppm shows reduced integral intensity corresponding to 24% D incorporation (Figure S22).

6.5 Radical trapping experiment with TEMPO.

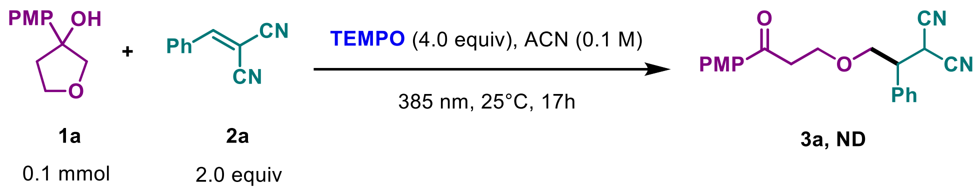

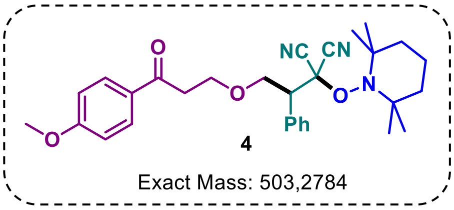

Compound detected by MS

A dry vial equipped with a magnetic stir bar was charged with **1a** (0.1 mmol, 1.0 equiv.), **2a** (0.2 mmol, 2.0 equiv.), **TEMPO** (0.4 mmol, 4.0 equiv.) and 1.0 mL of CH_3CN (0.1 M). Degasification of the reaction mixture was performed via freezepump-thaw cycling (3 x 10 min under vacuum). Then, the reaction mixture was irradiated and stirred in the photoreactor setup under 385 nm LED for 17h. Compound **4** was detected by MS (ESI) as reported in the Figure S23.

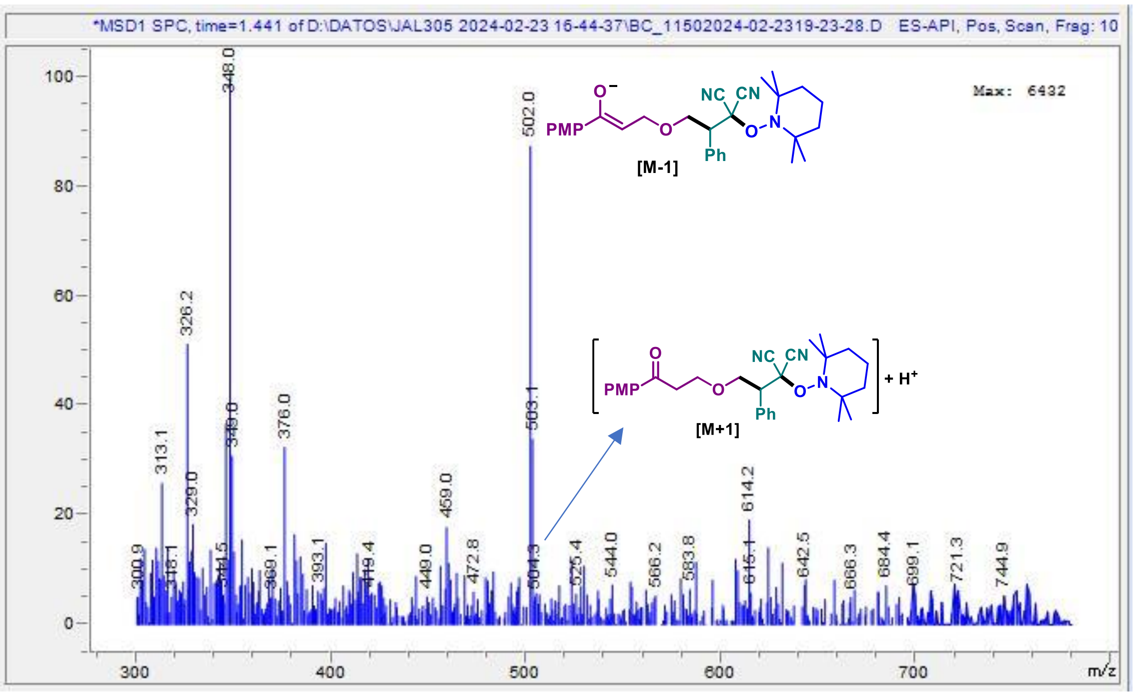

Figure S23. Radical trapping experiment with TEMPO.

6.6 Computational details. DFT calculations.

Computational details. Density functional theory and its time dependent version have been used, with the M062X functional combined with the 6-31G(d,p) basis set.[12] An implicit solvent model (Polarizable Continuum Model, PCM)[13] has been used to include CH_3CN in the calculations. To validate the DFT energies in the ground state and in gas phase for the different EDA complexes we resort to accurate CBS-QB3 methods.[14] All the calculations were done with Gaussian16 program.[15]

Stoichiometry

Different conformers for both EDA complexes with 1:1 and 1:2 (Figure S24) stoichiometry were optimized in the ground state in gas phase and in CH_3CN. Then, the difference between the sum of the free energies of the components (alcohol+double bond) was compared with the one of the EDA complexes. DFT energies (**Table S2**) are slightly favored (less positive energies) for the 1:1 stoichiometry, and in particular for the trans, compared to 1:2. Accurate CBS-

QB3 calculations (**Table S1**), supports the validity of the DFT energies although it seems to underestimate the stability of the EDA1-“cis” conformer. We then, move to CH_3CN solution (**Table S3**) where the trends observed in gas phase are maintained, 1:1 energies being half the 1:2 counterparts.

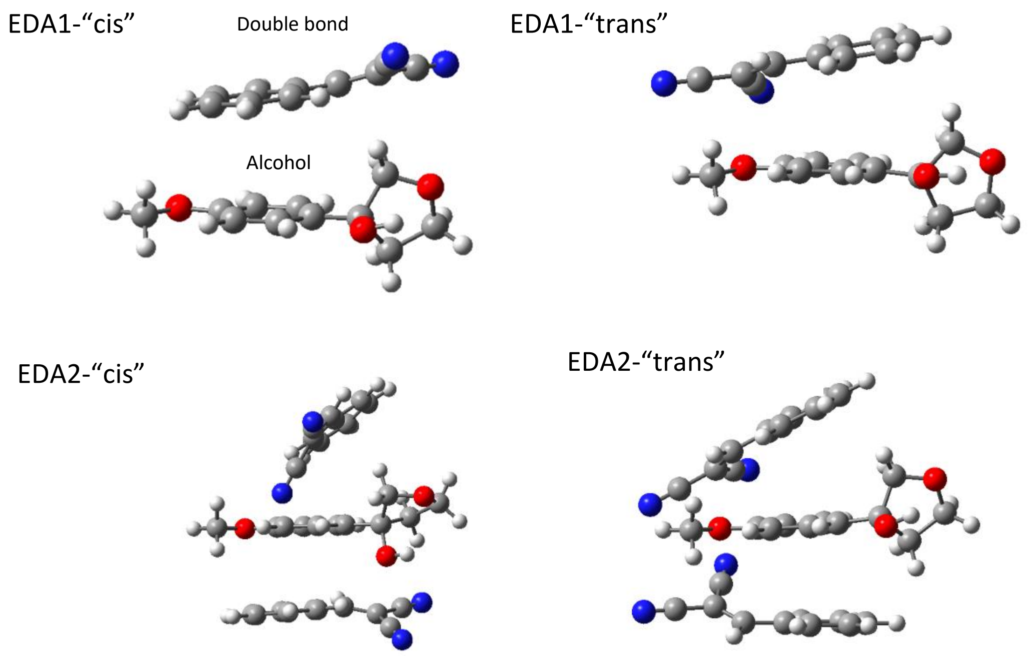

Figure S24. Ground state minima optimized for the 1:1 and 1:2 EDA complexes.

	Alcohol	Double bond			□G(kcal/mol)
	-652.193294	-493.315106	-1145.5084		
EDA1-“cis”				-1145.502669	**3.60**
EDA1-“trans”				-1145.502359	**3.79**

Table S2. Free energies computed at the accurate CBS-QB3 level of theory in the gas phase.

.

	Alcohol	Double bond			□G(kcal/mol)
	-652.806504	-493.849814	-1146.6563		

EDA1-“cis”				-1146.64762	**5.45**
EDA1-“trans”				-1146.65098	**3.35**
			-1640.5061		
EDA2-“trans”				-1640.493613	**7.86**
EDA2-“cis”				-1640.495073	**6.94**
EDA2-“cis/trans”				-1640.492045	**8.84**

Table S3. Free energies computed at the M062X/6-31+g(d,p) level of theory in the gas phase

	Alcohol	Double bond			□E(kcal/mol)
	-652.816318	-493.86178	-1146.6781		
EDA1-“cis”				-1146.668666	**5.92**
EDA1-“trans”				-1146.669713	**5.26**
			-1640.5399		
EDA2-“trans”				-1640.520446	**12.19**
EDA2-“cis”				-1640.522937	**10.63**

Table S4. Free energies computed at the M062X/6-31+g(d,p) level of theory in the CH_3CN.

Absorption Spectra

The absorption spectra were then simulated for the alcohol, double bond and EDA1 complexes by computing their energies and oscillator strengths at their optimized ground state structure using TD-DFT (**Table S4**).

	□E eV	□E nm	f

Alcohol			
S_1	5.21	237	0.040
S_2	5.90	210	0.265
Double Bond			
S_1	4.19	295	0.778
S_2	4.52	274	0.039
EDA1-“cis”			
S_1	3.76	330	0.070
S_2	4.14	300	0.533
EDA1-“trans”			
S_1	3.60	344	0.083
S_2	4.22	293	0.482

Table S5. Excited State absorption energies and oscillator strengths (f) computed at the TD-M062X/6-31+g(d,p) level of theory in the CH_3CN.

7 Experimental procedures and characterizations of products 3

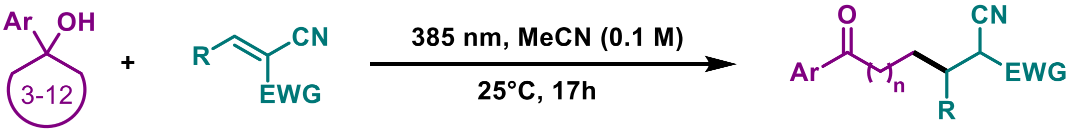

1 0.1 mmol

2 2.0 equiv

3

General procedure D for the Giese radical addition.

A dry vial equipped with a magnetic stir bar was charged with the corresponding alcohol (0.1 mmol, 1.0 equiv.), corresponding Michael acceptor (0.2 mmol, 2.0 equiv.), 2,6-lutidine (3 µL, 0.025 mmol, 0.25 equiv.) when is required and 1.0 mL of CH_3CN (0.1 M). Degasification of the reaction mixture was performed via freezepump-thaw cycling (3 x 10 min under vacuum). Then, the reaction mixture was irradiated and stirred in the photoreactor setup under 385 nm LED for 17 or 24 h. The reaction mixture was concentrated under reduced pressure and purified by flash column chromatography (silica gel) to provide the product **3.**

2-(2-(3-(4-methoxyphenyl)-3-oxopropoxy)-1-phenylethyl)malononitrile (3a)

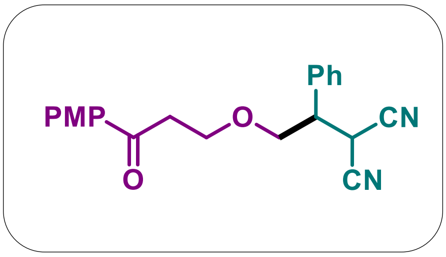

Following the general procedure D, from **1a** (0.1 mmol, 1.0 equiv.) and **2a** (0.2 mmol, 2.0 equiv.) after 17 h irradiation, **3a** was obtained as a yellowish oil after purification by column chromatography (silica, gradient from 12% to 37% of EtOAc in cyclohexane) in 80% yield (27.8 mg).

R$_f$ = 0.34 (2:1 cyclohexane:EtOAc).

^{1}H NMR (300 MHz, $CDCl_3$) δ 8.31 – 7.75 (m, 2H), 7.41 (dq, *J* = 6.1, 3.8 Hz, 5H), 7.06 – 6.94 (m, 2H), 4.48 (d, *J* = 5.7 Hz, 1H), 4.08 – 3.86 (m, 7H), 3.51 (dt, *J* = 8.9, 5.1 Hz, 1H), 3.27 (td, *J* = 6.0, 3.1 Hz, 2H).

^{13}C NMR (75 MHz, $CDCl_3$): δ 196.4, 163.8, 134.4, 130.5 (2C), 130.0, 129.2 (2C), 128.2 (2C), 113.9, 112.3, 111.8, 70.1, 66.8, 55.6, 46.2, 38.0, 26.2.

HRMS (ESI): calcd for $C_{21}H_{21}N_2O_3$ $[M+H]^+$: 349.1552, found: 349.1559

2-(2-(2-(4-methoxyphenyl)-2-oxoethoxy)-1-phenylethyl)malononitrile (3b)

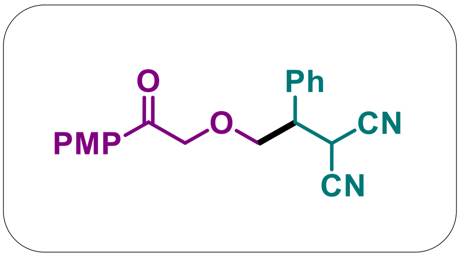

Following the general procedure D, from **1b** (0.1 mmol, 1.0 equiv.), **2a** (0.2 mmol, 2.0 equiv.) and 2,6 lutidine (3 μL, 0.025 mmol, 0.25 equiv.) as additive, after 24 h irradiation **3b** was obtained as a yellowish oil after purification by column chromatography (silica, gradient from 12% to 37% of EtOAc in cyclohexane) in 78% yield (26.0 mg).

R*f* = 0.18 (4:1 cyclohexane:EtOAc)

^{1}H NMR (300 MHz, $CDCl_3$): 7.89 (d, *J* = 8.9 Hz, 2H), 7.45 – 7.35 (m, 5H), 6.96 (d, *J* = 8.9 Hz, 2H), 4.84 (d, *J* = 6.4 Hz, 2H), 4.81 (d, *J* = 5.4 Hz, 1H), 4.02 (d, *J* = 3.6 Hz, 1H), 4.00 (s, 1H), 3.88 (s, 3H), 3.59 (dt, *J* = 8.3, 5.3 Hz, 1H).

^{13}C NMR (75 MHz, $CDCl_3$): 193.8, 164.2, 134.1, 130.1 (2C), 129.3, 129.3 (2C), 128.3 (2C), 127.4, 114.1 (2C), 112.4, 111.8, 73.4, 70.8, 55.6, 46.8, 26.5.

HRMS (ESI): calcd for $C_{20}H_{19}N_2O_3$ $[M+H]^+$: 335.1396, found: 335.1401

tert-butyl(3,3-dicyano-2-phenylpropyl)(4-(4-methoxyphenyl)-4oxobutyl)carbamate (3c)

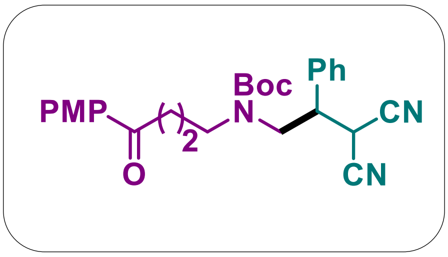

Following the general procedure D, from **1c** (0.1 mmol, 1.0 equiv.), **2a** (0.2 mmol, 2.0 equiv.) and 2,6 lutidine (3 μL, 0.025 mmol, 0.25 equiv.) as additive, after 17 h irradiation**, 3c** was obtained as a yellowish oil after purification by column chromatography (silica, gradient from 10% to 35% of EtOAc in cyclohexane) in 65% yield (23.4 mg).

R*f* = 0.09 (4:1 cyclohexane:EtOAc).

^{1}H NMR (300 MHz, $CDCl_3$): δ 7.91 (d, *J* = 8.9 Hz, 2H), 7.48 – 7.33 (m, 5H), 6.93 (d, *J* = 8.9 Hz, 2H), 4.22 (s, 1H), 3.97 (dd, *J* = 14.2, 8.6 Hz, 1H), 3.87 (s, 3H), 3.66 (s, 1H), 3.53 (dd, *J* = 14.2, 5.8 Hz, 1H), 3.26 (dt, *J* = 14.7, 7.4 Hz, 1H), 3.19 – 3.04 (m, 1H), 2.88 (t, *J* = 6.8 Hz, 2H), 1.98 – 1.79 (m, 2H), 1.44 (s, 9H).

These data are consistent with those previously reported in the literature for this compound.[6]

2-(4-(4-methoxyphenyl)-4-oxo-1-phenylbutyl)malononitrile (3d)

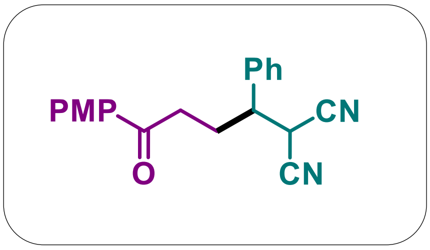

Following the general procedure D, from **1k** (0.1 mmol, 1.0 equiv.), **2a** (0.2 mmol, 2.0 equiv.) and 2,6 lutidine (3 μL, 0.025 mmol, 0.25 equiv.) as additive, after 17 h, **3d** was obtained as a yellowish oil after purification by column chromatography (silica, gradient from 10% to 35% of EtOAc in cyclohexane) in 53% yield (17.0 mg).

R_f = 0.09 (4:1 cyclohexane:EtOAc).

^{1}H NMR (300 MHz, $CDCl_3$): δ 7.84 – 7.77 (m, 2H), 7.44 – 7.37 (m, 3H), 7.35 – 7.31 (m, 2H), 6.91 – 6.82 (m, 2H), 4.02 (d, *J* = 6.2 Hz, 1H), 3.85 (s, 3H), 3.43 (ddd, *J* = 10.8, 6.3, 4.2 Hz, 1H), 2.86 (ddd, *J* = 7.6, 6.3, 1.7 Hz, 2H), 2.51 (dtd, *J* = 13.8, 7.5, 4.2 Hz, 1H), 2.36 (ddt, *J* = 13.9, 11.3, 6.3 Hz, 1H).

These data are consistent with those previously reported in the literature for this compound[16]

2-(7-(4-methoxyphenyl)-7-oxo-1-phenylheptyl)malononi-trile (3e)

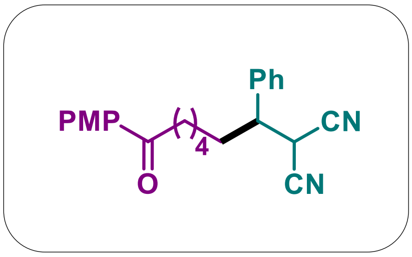

Following the general procedure D, from **1d** (0.1 mmol, 1.0 equiv.), **2a** (0.2 mmol, 2.0 equiv.) and 2,6 lutidine (3 μL, 0.025 mmol, 0.25 equiv.) as additive, after 17 h irradiation, **3e** was obtained as a yellowish oil after purification by column chromatography (silica, gradient from 11% to 35% of EtOAc in cyclohexane) in 71% yield (25.6 mg).

R_f = 0.18 (4:1 cyclohexane:EtOAc).

^{1}H NMR (300 MHz, $CDCl_3$): δ 7.91 (d, *J* = 8.9 Hz, 2H), 7.48 – 7.33 (m, 3H), 7.33 – 7.27 (m, 2H), 6.92 (d, *J* = 8.9 Hz, 2H), 3.88 (d, *J* = 6.3 Hz, 1H), 3.87 (s, 3H), 3.26 – 3.15 (m, 1H), 2.86 (t, *J* = 7.2 Hz, 2H), 2.10 – 1.96 (m, 2H), 1.84 – 1.58 (m, 2H), 1.48 – 1.18 (m, 4H).

These data are consistent with those previously reported in the literature for this compound.[6]

2-(13-(4-methoxyphenyl)-13-oxo-1-phenyltridecyl)malononitrile (3f)

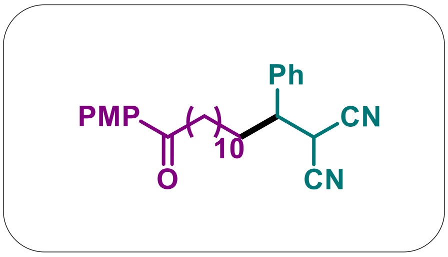

Following the general procedure D, from **1e** (0.1 mmol, 1.0 equiv.), **2a** (0.2 mmol, 2.0 equiv.) and 2,6 lutidine (3 μL, 0.025 mmol, 0.25 equiv.) as additive, after 17 h irradiation, **3f** was obtained as a pale yellowish oil after purification by column chromatography (silica, gradient from 7% to 20% of EtOAc in cyclohexane) in 55% yield (24.5 mg).

$\mathbf{R}_f$ = 0.30 (4:1 cyclohexane:EtOAc).

^{1}H NMR (300 MHz, CDCl$_3$): δ 7.94 (d, J = 8.9 Hz, 2H), 7.47 – 7.35 (m, 3H), 7.34 – 7.28 (m, 2H), 6.93 (d, J = 8.9 Hz, 2H), 3.89 (d, J = 6.3 Hz, 1H), 3.87 (s, 3H), 3.24 – 3.14 (m, 1H), 2.94 – 2.85 (m, 2H), 1.99 (q, J = 8.1 Hz, 2H), 1.71 (p, J = 7.4 Hz, 2H), 1.40 – 1.14 (m, 17H).

These data are consistent with those previously reported in the literature for this compound.[6]

2-(2-(4-(furan-2-yl)-4-oxobutoxy)-1-phenylethyl)malononitrile (3g)

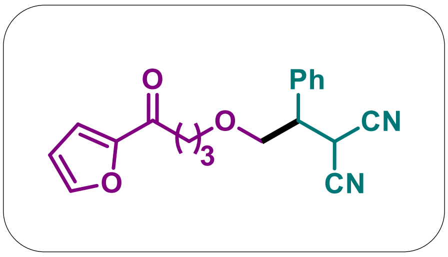

Following the general procedure D, from **1f** (0.1 mmol, 1.0 equiv.), **2a** (0.2 mmol, 2.0 equiv.) and 2,6 lutidine (3 μL, 0.025 mmol, 0.25 equiv.) as additive, after 17 h irradiation**, 3g** was obtained as a pale yellowish oil after purification by column chromatography (silica, gradient from 10% to 20% of EtOAc in cyclohexane) in 62% yield (19.9 mg).

$\mathbf{R}_f$ = 0.21 (4:1 cyclohexane:EtOAc).

^{1}H NMR (300 MHz, CDCl$_3$): δ 7.61 (dd, J = 1.8, 0.8 Hz, 1H), 7.41 – 7.32 (m, 5H), 7.22 (dd, J = 3.6, 0.8 Hz, 1H), 6.56 (dd, J = 3.6, 1.7 Hz, 1H), 4.34 (d, J = 5.9 Hz, 1H), 3.90 – 3.73 (m, 2H), 3.61 (t, J = 6.0 Hz, 2H), 3.40 (ddd, J = 8.4, 6.0, 4.7 Hz, 1H), 2.95 (t, J = 7.0 Hz, 2H), 2.12 – 2.03 (m, 2H).

These data are consistent with those previously reported in the literature for this compound.[6]

2-(2-(4-(benzofuran-2-yl)-4-oxobutoxy)-1-phenylethyl)malononitrile (3h)

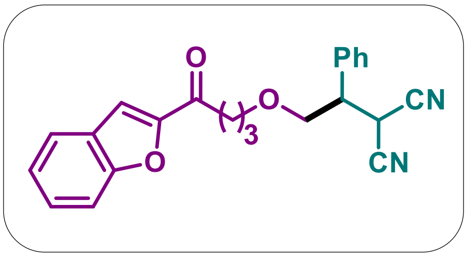

Following the general procedure D, from **1g** (0.1 mmol, 1.0 equiv.), **2a** (0.2 mmol, 2.0 equiv.) and 2,6 lutidine (3 µL, 0.025 mmol, 0.25 equiv.) as additive, after 17 h irradiation, **3h** was obtained as a yellowish oil after purification by column chromatography (silica, gradient from 7% to 20% of EtOAc in cyclohexane) in 44% yield (15.7 mg).

R_f = 0.13 (4:1 cyclohexane:EtOAc).

^{1}H NMR (300 MHz, $CDCl_3$): δ 7.76 – 7.70 (m, 1H), 7.60 (dq, *J* = 8.4, 0.9 Hz, 1H), 7.54 (d, *J* = 1.0 Hz, 1H), 7.38 - 7.30 (m, 1H), 4.37 (d, *J* = 6.0 Hz, 1H), 3.92 – 3.77 (m, 2H), 3.65 (t, *J* = 6.0 Hz, 2H), 3.39 (ddd, *J* = 8.4, 6.0, 4.7 Hz, 1H), 3.09 (t, *J* = 7.0 Hz, 2H), 2.16 – 2.11 (m, 2H).

These data are consistent with those previously reported in the literature for this compound.[6]

2-(2-(4-oxo-4-(phenanthren-9-yl)butoxy)-1-phenylethyl)malononitrile (3i)

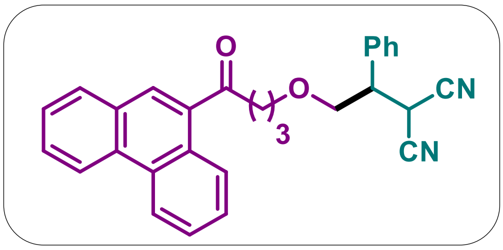

Following the general procedure D, from **1h** (0.1 mmol, 1.0 equiv.), **2a** (0.2 mmol, 2.0 equiv.) and 2,6 lutidine (3 µL, 0.025 mmol, 0.25 equiv.) as additive**,** after 17 h irradiation**, 3i** was obtained as a yellowish oil after purification by column chromatography (silica, gradient from 9% to 23% of EtOAc in cyclohexane) in 50% yield (21.6 mg).

R_f = 0.38 (4:1 cyclohexane:EtOAc).

^{1}H NMR (300 MHz, $CDCl_3$): δ 8.77 – 8.66 (m, 2H), 8.64 – 8.54 (m, 1H), 8.17 (s, 1H), 7.97 (dd, *J* = 8.0, 1.5 Hz, 1H), 7.80 – 7.64 (m, 4H), 7.39 – 7.31 (m, 5H), 4.34 (d, *J* = 5.9 Hz, 1H), 3.92 – 3.78 (m, 2H), 3.70 (t, *J* = 6.1 Hz, 2H), 3.46 – 3.36 (m, 1H), 3.27 (t, *J* = 7.1 Hz, 2H), 2.26 – 2.11 (m, 2H).

These data are consistent with those previously reported in the literature for this compound.[6]

2-(7-(4-methoxyphenyl)-3-methyl-7-oxo-1-phenylheptyl)malononitrile (3j)

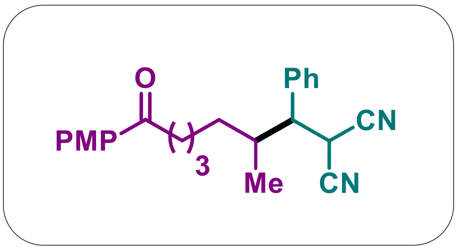

Following the general procedure D, from **1j** (0.1 mmol, 1.0 equiv.) and **2a** (0.2 mmol, 2.0 equiv.), after 17 h, **3j** was obtained as an inseparable 1:1 mixture of the two possible diastereoisomers, as a colourless oil after purification by column chromatography (silica, gradient from 7% to 55% of EtOAc in cyclohexane) in 70% yield (26.2 mg).

R*f* = 0.15 (4:1 cyclohexane:EtOAc).

^{1}H NMR (300 MHz, CDCl$_3$): δ 7.99 (d, *J* = 8.4 Hz, 2H), 7.91 (d, *J* = 8.5 Hz, 2H), 7.42 (d, *J* = 6.7 Hz, 6H), 7.39 – 7.24 (m, 4H), 6.97 (t, *J* = 8.4 Hz, 4H), 4.27 (d, *J* = 6.9 Hz, 1H), 4.22 (d, *J* = 5.5 Hz, 1H), 3.90 (s, 3H), 3.90 (s, 3H), 3.12 – 2.88 (m, 4H), 2.83 (t, *J* = 7.2 Hz, 2H), 2.27 (q, *J* = 8.4, 7.8 Hz, 6H), 1.92 – 1.72 (m, 2H), 1.71 – 1.40 (m, 6H), 1.37 - 1.25 (m, 4H), 1.16 (d, *J* = 6.6 Hz, 3H), 0.86 (d, *J* = 6.7 Hz, 3H).

^{13}C NMR (75 MHz, CDCl$_3$: δ 198.7, 198.7, 163.5, 163.4, 136.7, 136.2, 130.3 (2C), 130.3 (2C), 130.1, 129.2 (2C), 129.1 (2C), 128.8, 128.8, 128.4 (2C), 128.3 (2C), 113.8 (2C), 113.7 (2C), 112.3, 112.2, 112.1, 111.9, 55.5 (2C), 52.3, 51.4, 37.8, 37.8, 34.7 (2C), 34.2, 33.3, 27.8, 27.4, 26.0, 26.0, 24.4, 24.2, 17.4, 16.3.

HRMS (ESI): calcd for $C_{24}H_{27}N_2O_2$ $[M+H]^+$: 375.2073, found: 375.2077

2-(7-(4-methoxyphenyl)-7-oxo-1,2-diphenylheptyl)malononitrile (3k)

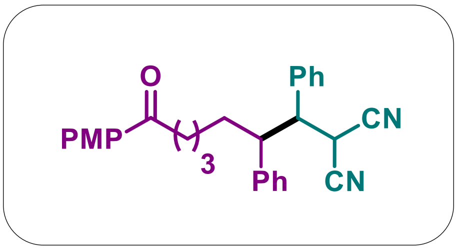

Following the general procedure D, from **1i** (0.1 mmol, 1.0 equiv.) and **2a** (0.2 mmol, 2.0 equiv.), after 17 h irradiation, **3k** was obtained as an inseparable 1:1 mixture of the two possible diastereoisomers, as a colourless oil after purification by column chromatography (silica,

gradient from 7% to 55% of EtOAc in cyclohexane) in 70% yield (30.5 mg).

R*f* = 0.15 (4:1 cyclohexane:EtOAc).

^{1}H NMR (300 MHz, $CDCl_3$): δ 7.88 (d, *J* = 8.9 Hz, 2H), 7.81 (d, *J* = 8.9 Hz, 2H), 7.56 – 7.39 (m, 7H), 7.35 (tt, *J* = 7.8, 1.4 Hz, 3H), 7.27 – 7.16 (m, 7H), 6.94 – 6.82 (m, 7H), 4.03 (dd, *J* = 8.8, 0.9 Hz, 1H), 3.86 (s, 3H), 3.85 (s, 3H), 3.59 – 3.46 (m, 2H), 3.40 – 3.25 (m, 1H), 3.28 – 3.15 (m, 2H), 2.83 (t, *J* = 7.3 Hz, 2H), 2.67 (ddd, *J* = 7.8, 6.8, 1.1 Hz, 2H), 1.84 – 1.55 (m, 5H), 1.55 – 1.39 (m, 3H), 1.39 – 1.17 (m, 3H), 1.16 – 0.98 (m, 2H).

These data are consistent with those previously reported in the literature for this compound.[6]

ethyl (3-2-cyano-4-(3-(4-methoxyphenyl)-3-oxopropoxy)-3-phenylbutanoate (3l)

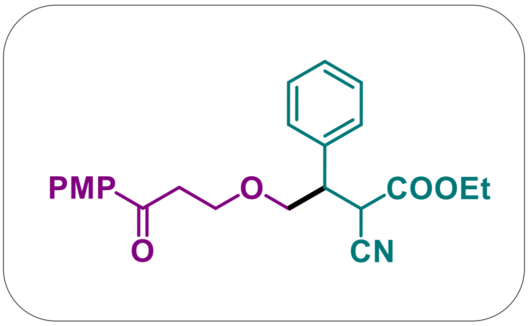

Following the general procedure D, from **1a** (0.1 mmol, 1.0 equiv.) and **2h** (0.2 mmol, 2.0 equiv.) and 2,6 lutidine (3 μL, 0.025 mmol, 0.25 equiv.) as additive**,** after 17 h irradiation**, 3l** was obtained as an inseparable 1:1 mixture of the two possible diastereoisomers as a yellowish oil after purification by column chromatography (silica, gradient from 20% to 50% of EtOAc in cyclohexane) in 61% yield (23.9 mg).

R*f* = 0.10 (4:1 cyclohexane:EtOAc).

^{1}H NMR (300 MHz, $CDCl_3$): δ 7.97 (d, *J* = 4.0 Hz, 2H), 7.94 (d, *J* = 6.0 Hz, 2H), 7.35 – 7.26 (m, 10H), 6.96 (d, *J* = 2.0 Hz, 2H), 6.93 (d, *J* = 2.0 Hz, 2H), 4.19 (d, *J* = 5.5 Hz, 1H), 4.14 – 4.03 (m, 4H), 4.03 – 3.94 (m, 2H), 3.94 – 3.88 (m, 4H), 3.88 (s, 3H), 3.87 (s, 3H), 3.87 – 3.80 (m, 2H), 3.77 (d, *J* = 4.6 Hz, 1H), 3.74 (d, *J* = 4.6 Hz, 1H), 3.70 – 3.60 (m, 2H), 3.27 – 3.11 (m, 4H), 1.15 (t, *J* = 7.2 Hz, 3H), 1.10 (t, *J* = 7.1 Hz, 3H).

^{13}C NMR (75 MHz, $CDCl_3$: δ 196.5, 196.5, 165.5, 165.4, 163.7, 163.6, 137.4, 135.9, 130.5 (2C), 130.1, 130.1 (2C), 128.9 (2C), 129.0 (2C), 128.4 (2C), 128.3

(2C), 128.1, 128.0 (2C), 115.8, 115.5, 113.8 (2C), 114.0 (2C), 71.5, 71.4, 67.0, 66.7, 62.6, 62.5, 55.5 (2C), 45.7, 45.4, 41.7, 40.8, 38.2, 38.1, 13.9 (2C).

HRMS (ESI): calcd for $C_{23}H_{26}NO_5$ $[M+H]^+$: 396.1811, found: 396.1815.

4-(3-(4-methoxyphenyl)-3-oxopropoxy)-3-phenyl-2-(phenylsulfonyl)butanenitrile (3m)

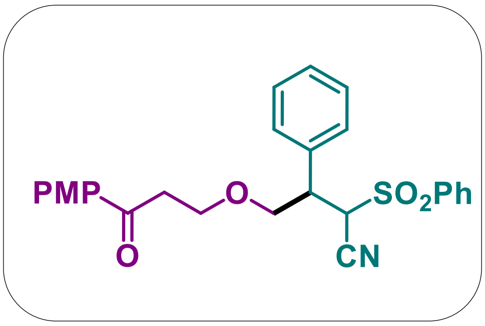

Following the general procedure D, from **1a** (0.1 mmol, 1.0 equiv.) and **2i** (0.2 mmol, 2.0 equiv.) using 2,6 lutidine (3 μL, 0.025 mmol, 0.25 equiv.) as additive**,** after 17 h irradiation**, 3m** was obtained as an inseparable 1:3 mixture of the two possible diastereoisomers as a yellowish oil after purification by column chromatography (silica, gradient from 20% to 50% of EtOAc in cyclohexane) in 51% yield (23.2 mg).

R_f = 0.15 (4:1 cyclohexane:EtOAc).

^{1}H NMR (300 MHz, $CDCl_3$): δ 7.92 – 7.80 (m, 3H), 7.77 – 7.68 (m, 2H), 7.64 – 7.43 (m, 2H), 7.43 – 7.31 (m, 2H), 7.32 – 7.23 (m, 2H), 7.22 – 7.15 (m, 6H), 6.94 – 6.80 (m, 3H), 4.74 (d, *J* = 4.4 Hz, 1H), 4.23 (d, *J* = 5.3 Hz, 1H), 3.99 – 3.92 (m, 1H), 3.92 – 3.84 (m, 2H), 3.81 (s, 3H), 3.80 (s, 3H), 3.79 – 3.75 (m, 1H), 3.74 – 3.65 (m, 2H), 3.17 – 3.10 (m, 2H), 3.10 – 3.00 (m, 2H).

^{13}C NMR (75 MHz, $CDCl_3$: δ 196.8, 196.4, 163.8, 163.6, 137.6, 137.0, 137.0, 135.0, 134.6, 134.6, 130.5, 130.5 (2C), 130.2, 130.0, 129.5, 129.4 (2C), 129.4, 129.1 (2C), 128.9 (2C), 128.9, 128.8 (2C), 128.6, 128.2, 128.1, 113.9 (2C), 113.7 (2C), 113.5, 113.2, 71.5, 71.5, 67.2, 66.4, 60.8, 58.7, 55.5, 55.5, 43.3, 43.1, 38.1, 38.0, 26.9.

HRMS (ESI): calcd for $C_{26}H_{26}NO_5S$ $[M+H]^+$: 464.1532, found: 464.1529.

2-(1-(4-chlorophenyl)-2-(3-(4-methoxyphenyl)-3-oxopropoxy)ethyl)malononitrile (3n)

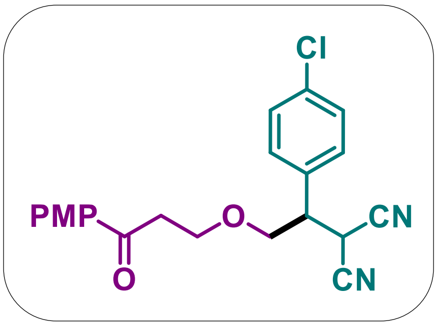

Following the general procedure D, from **1a** (0.1 mmol, 1.0 equiv.) and **2d** (0.2 mmol, 2.0 equiv.), after 17 h irradiation**, 3n** was obtained as a yellowish oil after purification by column chromatography (silica, gradient from 20% to 50% of EtOAc in cyclohexane) in 91% yield (34.7 mg).

Rf = 0.37 (2:1 cyclohexane:EtOAc).

^{1}H NMR (300 MHz, $CDCl_3$): δ 7.96 (d, *J* = 8.9 Hz, 2H), 7.37 (d, *J* = 8.6 Hz, 2H), 7.29 (d, *J* = 8.6 Hz, 2H), 6.97 (d, *J* = 8.9 Hz, 2H), 4.42 (d, *J* = 5.9 Hz, 1H), 4.05 – 3.84 (m, 7H), 3.45 (dt, *J* = 7.9, 5.5 Hz, 1H), 3.22 (td, *J* = 6.1, 2.0 Hz, 2H).

^{13}C NMR (75 MHz, $CDCl_3$): δ 196.3, 163.8, 135.3, 132.9, 130.5 (2C), 129.9, 129.6 (2C), 129.5 (2C), 113.9 (2C), 112.1, 111.6, 69.9, 66.8, 55.6, 45.7, 37.9, 26.1.

HRMS (ESI): calcd for $C_{21}H_{20}ClN_2O_3$ $[M+H]^+$: 383.1162, found: 383.1166

2-(1-(3,5-bis(trifluoromethyl)phenyl)-2-(3-(4-methoxyphenyl)-3-oxopropoxy)ethyl) malononitrile (3o)

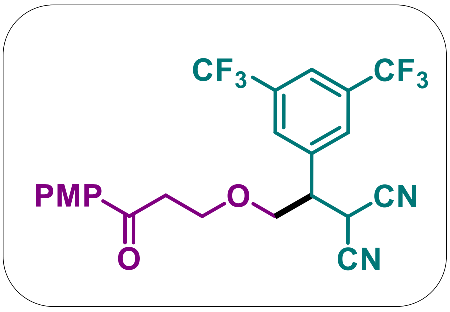

Following the general procedure D, from **1a** (0.1 mmol, 1.0 equiv.) and **2c** (0.2 mmol, 2.0 equiv.), after 17 h irradiation**, 3o** was obtained as a yellowish oil after purification by column chromatography (silica, gradient from 20% to 50% of EtOAc in cyclohexane) in 98% yield (47.4mg).

Rf = 0.41 (2:1 cyclohexane:EtOAc).

^{1}H NMR (300 MHz, $CDCl_3$): δ 8.00 (d, *J* = 8.9 Hz, 2H), 7.96 (s, 1H), 7.89 (d, *J* = 1.6 Hz, 2H), 7.00 (d, *J* = 8.9 Hz, 2H), 4.56 (d, *J* = 7.0 Hz, 1H), 4.17 – 3.94 (m, 4H), 3.92 (s, 3H), 3.71 – 3.57 (m, 1H), 3.39 – 3.15 (m, 2H).

^{13}C NMR (75 MHz, $CDCl_3$): δ 196.1, 163.9, 137.4, 132.6 (q, *J* = 33.7 Hz, 2C), 130.5 (2C), 129.8, 128.7 (d, *J* = 3.9 Hz, 2C), 123.3 (p, *J* = 3.7 Hz), 122.9 (q, *J* = 272.9 Hz, 2C). 114.0 (2C), 111.6, 111.2, 69.4, 66.9, 55.6, 46.0, 37.8, 25.8.

^{19}F NMR (283 MHz, CDCl3): δ -62.95.

HRMS (ESI): calcd for $C_{23}H_{19}F_6N_2O_3$ $[M+H]^+$: 485.1300, found: 485.1304.

2-(1-(4-cyanophenyl)-7-(4-methoxyphenyl)-7-oxoheptyl)malononitrile (3p)

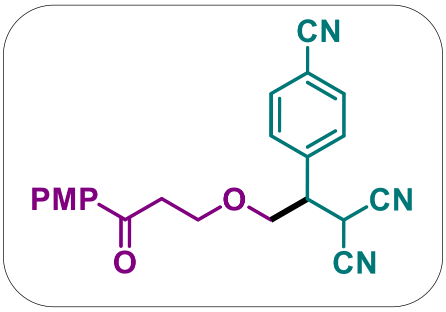

Following the general procedure D, from **1a** (0.1 mmol, 1.0 equiv.), **2b** (0.2 mmol, 2.0 equiv.), after 24h irradiation **3p** was obtained as a yellowish oil after purification by column chromatography (silica, gradient from 20% to 50% of EtOAc in cyclohexane) in 50% yield (18.5 mg).

$\mathbf{R}_f$ = 0.15 (4:1 cyclohexane:EtOAc).

^{1}H NMR (300 MHz, CDCl$_3$): δ 7.96 (d, *J* = 8.9 Hz, 2H), 7.69 (d, *J* = 8.4 Hz, 2H), 7.48 (d, *J* = 8.3 Hz, 2H), 6.97 (d, *J* = 8.9 Hz, 2H), 4.48 (d, *J* = 6.3 Hz, 1H), 4.06 – 3.86 (m, 7H), 3.53 (q, *J* = 6.3 Hz, 1H), 3.22 (t, *J* = 5.8 Hz, 2H), 3.11 (qd, *J* = 7.3, 4.7 Hz, 2H).

^{13}C NMR (75 MHz, CDCl$_3$): δ 196.2, 163.9, 139.6, 132.9 (2C), 130.5 (2C), 129.8, 129.2 (2C), 118.0, 114.0 (2C), 113.4, 111.8, 111.3, 69.5, 66.8, 55.6, 46.2, 37.8, 25.7.

HRMS (ESI): calcd for $C_{22}H_{20}N_3O_3$ $[M+H]^+$: 374.1505, found: 374.1507

2-(1-(4-fluorophenyl)-2-(3-(4-methoxyphenyl)-3-oxopropoxy)ethyl)malononitrile (3q)

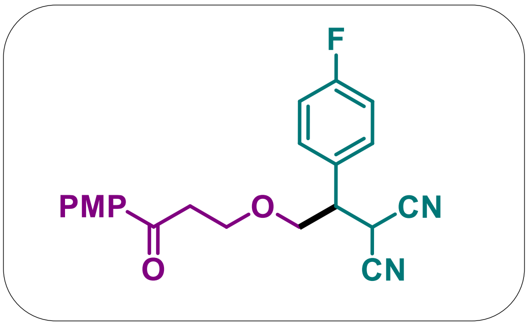

Following the general procedure D, from **1a** (0.1 mmol, 1.0 equiv.) and **2e** (0.2 mmol, 2.0 equiv.), after 24 h irradiation**,** **3q** was obtained as a yellowish oil after purification by column chromatography (silica, gradient from 20% to 50% of EtOAc in cyclohexane) in 72% yield (26.3 mg).

$\mathbf{R}_f$ = 0.32 (2:1 cyclohexane:EtOAc).

^{1}H NMR (300 MHz, CDCl$_3$): δ 8.00 (d, *J* = 9.0 Hz, 2H), 7.37 (ddt, *J* = 8.2, 5.2, 2.6 Hz, 2H), 7.19 – 7.05 (m, 2H), 7.06 – 6.95 (m, 2H), 4.46 (d, *J* = 5.8 Hz, 1H), 4.09

– 3.94 (m, 2H), 3.92 (s, 3H), 3.91 – 3.85 (m, 2H), 3.51 (dt, *J* = 8.3, 5.3 Hz, 1H), 3.33 – 3.21 (m, 2H).

^{13}C NMR (75 MHz, CDCl$_3$: δ 196.3, 163.8, 163.1 (d, *J* = 248.5 Hz), 130.5 (2C), 130.3, 130.1 (q, *J* = 8.4 Hz), 116.3 (d, *J* = 21.6 Hz), 113.9 (2C), 112.2, 111.6, 70.0, 66.7, 55.6, 45.6, 37.9, 26.3.

^{19}F NMR (283 MHz, CDCl$_3$): δ -111.99.

HRMS (ESI): calcd for $C_{21}H_{19}FN_2O_3$ [M+H]$^+$: 367.1458, found: 367.1455.

2-(1-(4-(tert-butyl)phenyl) -2 -(3-(4-methoxyphenyl) 3 oxopropoxy) ethyl) malononitrile (3r)

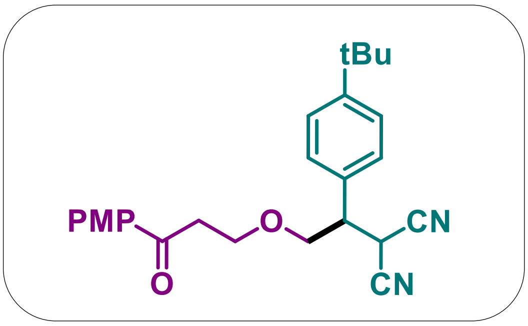

Following the general procedure D, from **1a** (0.1 mmol, 1.0 equiv.) and **2f** (0.2 mmol, 2.0 equiv.) after 17 h irradiation, **3r** was obtained as a yellowish oil after purification by column chromatography (silica, gradient from 20% to 50% of EtOAc in cyclohexane) in 92% yield (37.3 mg).

R$_f$ = 0.49 (2:1 cyclohexane:EtOAc).

^{1}H NMR (300 MHz, CDCl$_3$): δ 8.04 – 7.98 (m, 2H), 7.46 – 7.38 (m, 2H), 7.35 – 7.28 (m, 2H), 7.06 – 6.95 (m, 2H), 4.46 (d, *J* = 5.5 Hz, 1H), 4.09 – 3.84 (m, 7H), 3.49 (dt, *J* = 9.5, 4.9 Hz, 1H), 3.31 – 3.21 (m, 2H), 1.35 (s, 9H).

^{13}C NMR (75 MHz, CDCl$_3$: δ 196.4, 163.8, 152.2, 131.2, 130.5 (2C), 123.0, 127.9 (2C), 126.1 (2C), 113.9 (2C), 112.5, 111.9, 70.2, 66.7, 55.6, 45.8, 38.0, 34.6, 31.3 (3C), 26.3.

HRMS (ESI): calcd for $C_{25}H_{29}N_2O_3$ [M+H]$^+$: 405.2178, found: 405.2172

2-(2-(3-(4-methoxyphenyl)-3-oxopropoxy)-1-(m-tolyl)ethyl)malononitrile (3s)

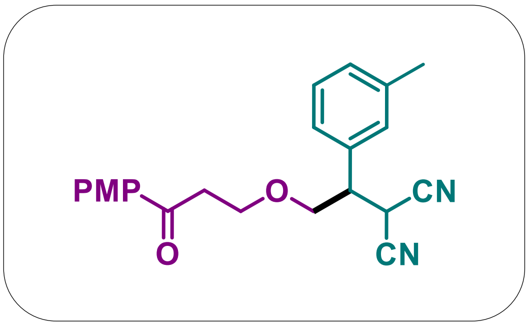

Following the general procedure D, from **1a** (0.1 mmol, 1.0 equiv.) and **2g** (0.2 mmol, 2.0 equiv.), after 24 h irradiation, **3s** was obtained as a yellowish oil after purification by column chromatography (silica, gradient from 20% to 50% of EtOAc in cyclohexane) in 27% yield (9.8 mg).

R_f = 0.41 (2:1 cyclohexane:EtOAc).

^{1}H NMR (300 MHz, $CDCl_3$): δ 8.02 – 7.93 (m, 2H), 7.30 – 7.11 (m, 5H), 6.96 (d, *J* = 8.9 Hz, 2H), 4.41 (d, *J* = 5.7 Hz, 1H), 4.06 – 3.90 (m, 1H), 3.95 – 3.90 (m, 1H), 3.88 (s, 3H), 3.88 – 3.79 (m, 2H), 3.43 (dt, *J* = 8.9, 5.1 Hz, 1H), 3.30 – 3.18 (m, 2H), 2.36 (s, 3H).

^{13}C NMR (75 MHz, $CDCl_3$: δ 196.4, 163.8, 139.0, 134.3, 130.5 (2C), 130.0, 130.0, 129.1, 128.9, 125.2, 113.9 (2C), 112.3, 111.8, 70.2, 66.7, 55.6, 46.2, 37.9, 26.2, 21.5.

HRMS (ESI): calcd for $C_{22}H_{23}N_2O_3$ $[M+H]^+$: 363.1709, found: 363.1711

8 General procedure for the photo-flow synthesis of product 3a

A dry vial equipped with a magnetic stir bar was charged with the alcohol **1a** (2.0 mmol, 1.0 equiv.), Michael acceptor **2a** (4.0 mmol, 2.0 equiv.) and 20 mL of CH_3CN (0.1 M). Degasification of the reaction mixture was performed via freeze-pump-thaw cycling (3 x 10 min under vacuum.

The flow system consist of a peristaltic pump connected to the coil reactor (10 mL), made of FEP capillary tube (1/16”OD; 0.5 mm ID) wrapped around a Pyrex beaker (80 mm diameter; 140 mm height). The coil was irradiated with two Kessil PR160L; (40 W, 390 nm) assembled on the sides of the reactor, together with a fan to maintain the temperature constant. The flow reaction was performed under light irradiation with a flow rate of 0.01 mL/min at room temperature (t_R = 16 h). The reaction mixture was concentrated under reduced pressure and purified by

flash column chromatography (silica gel) to provide the product **3a** in 66% yield. (Scheme S1).

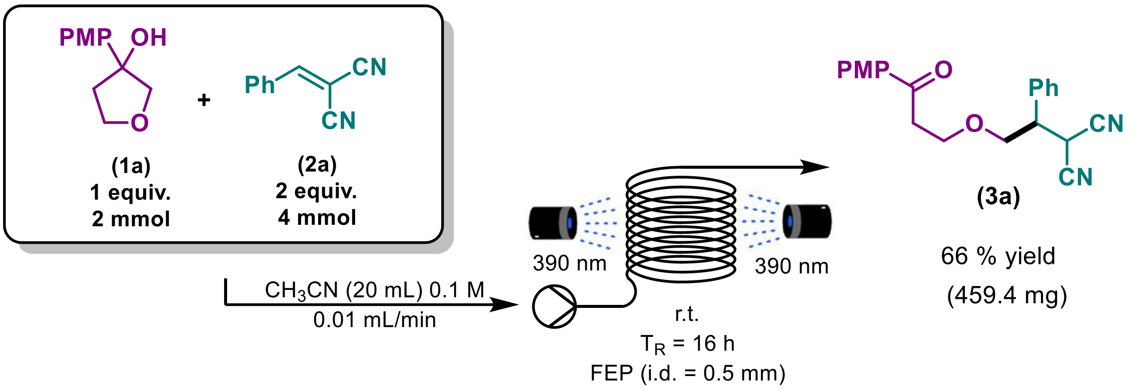

Scheme S1. Flow reaction scheme.

9 NMR Spectra

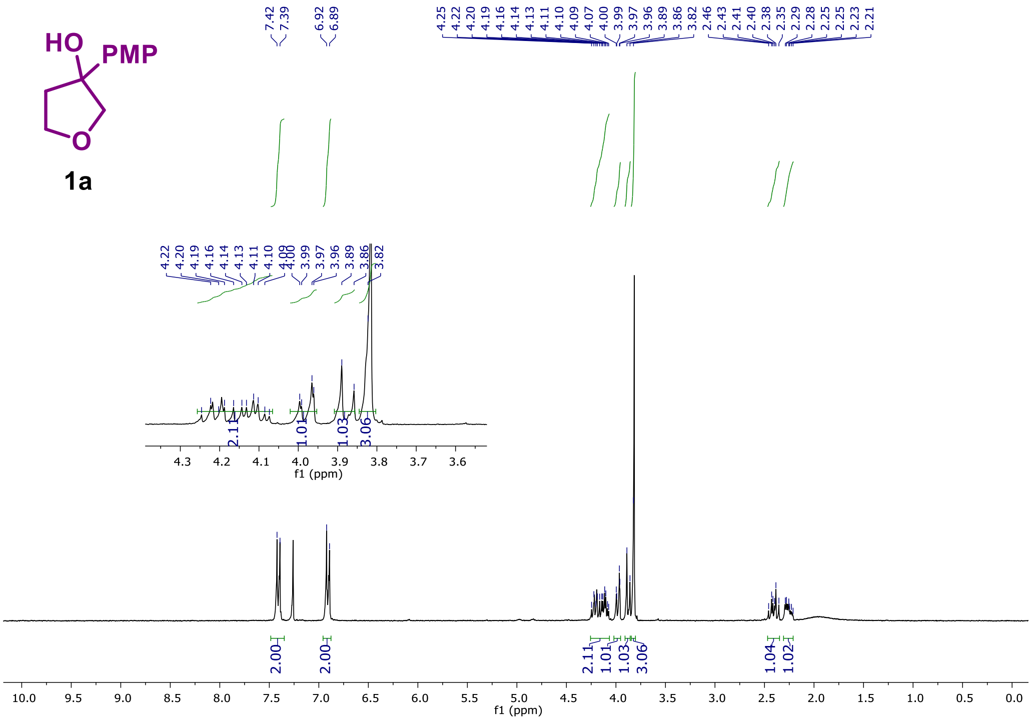

Figure S25. ^{1}H NMR spectrum (300 MHz, 298K, $CDCl_3$) of **1a.**

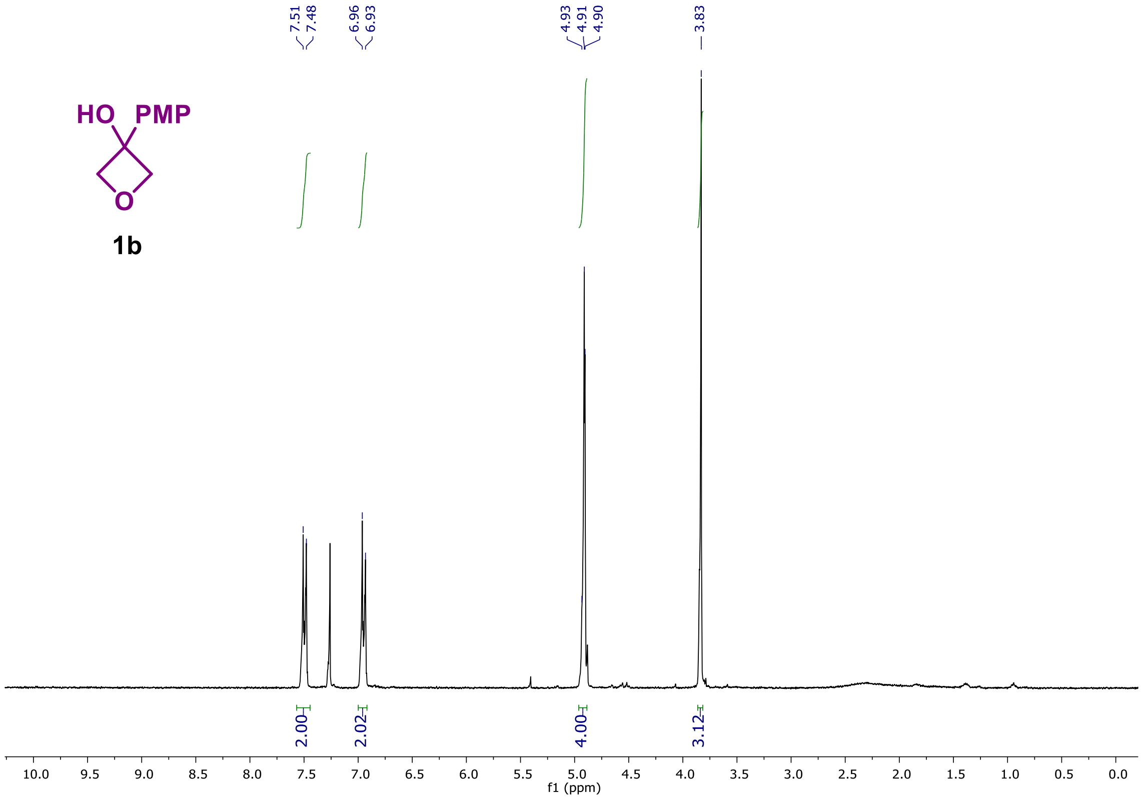

Figure S26. ^{1}H NMR spectrum (300 MHz, 298K, $CDCl_3$) of **1b.**

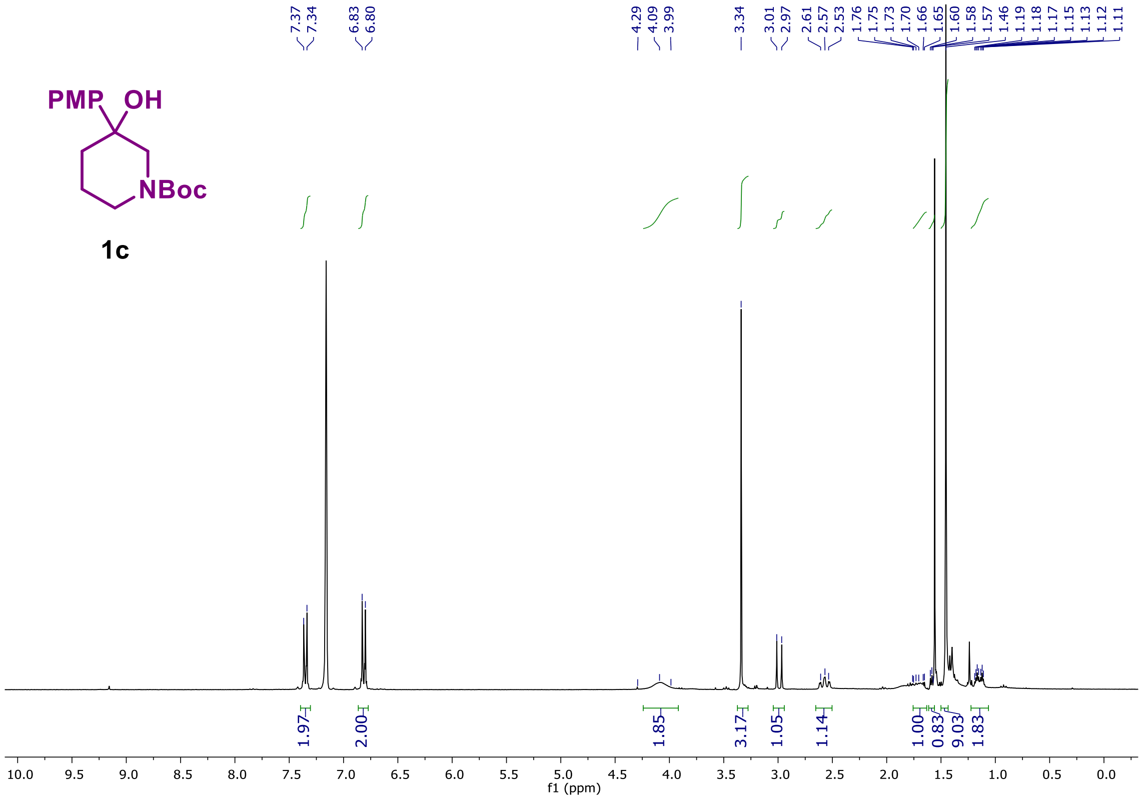

Figure S27. ^{1}H NMR spectrum (300 MHz, 298K, C_6D_6) of **1c.**

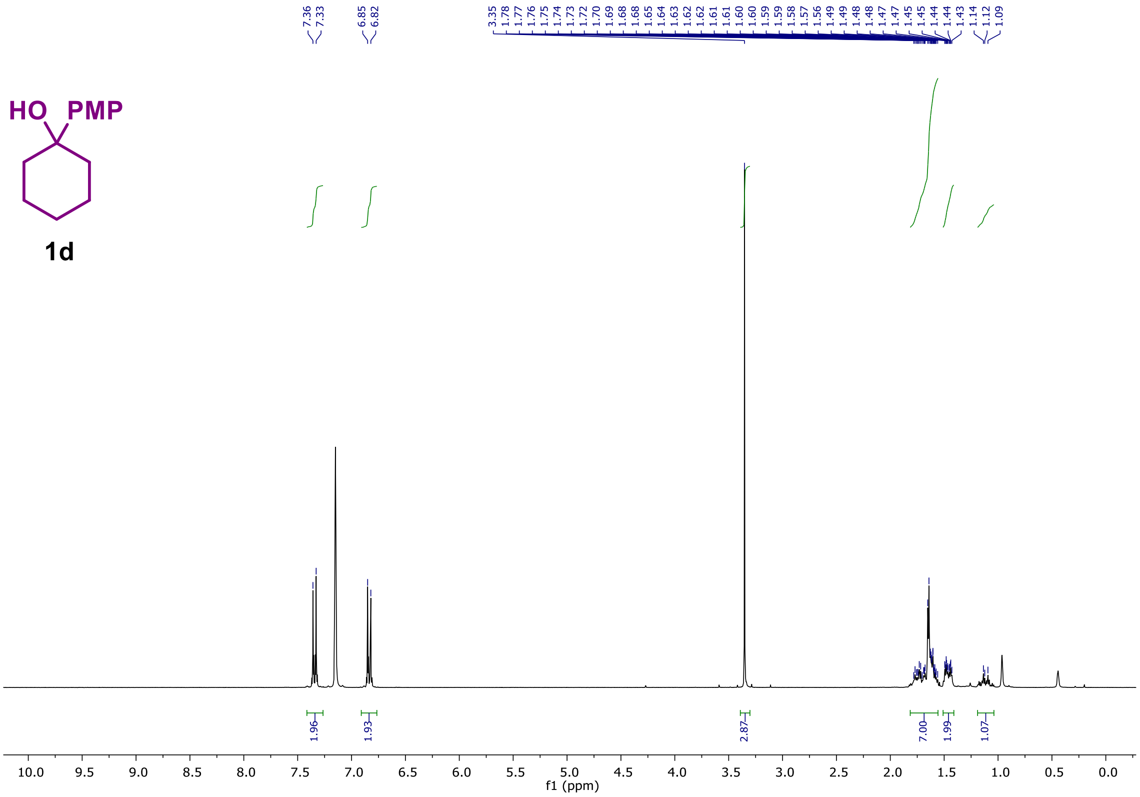

Figure S28. ^{1}H NMR spectrum (300 MHz, 298K, C_6D_6) of **1d.**

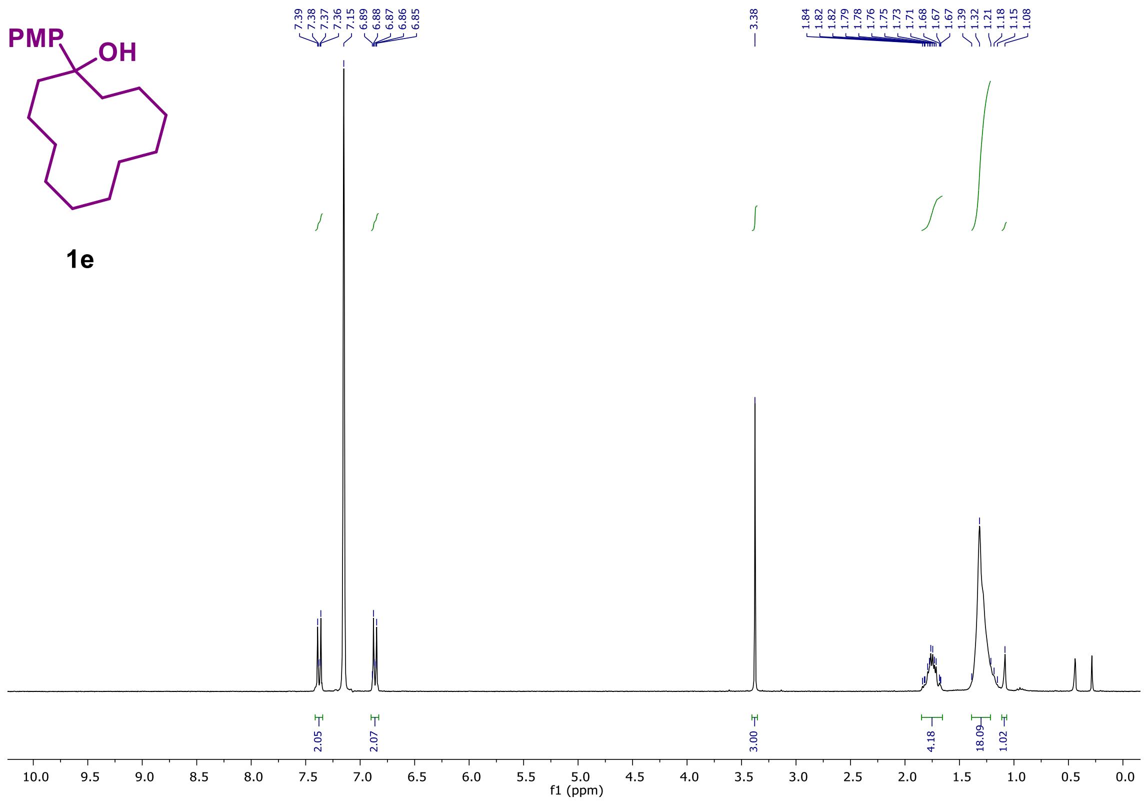

Figure S29. ^{1}H NMR spectrum (300 MHz, 298K, C_6D_6) of **1e.**

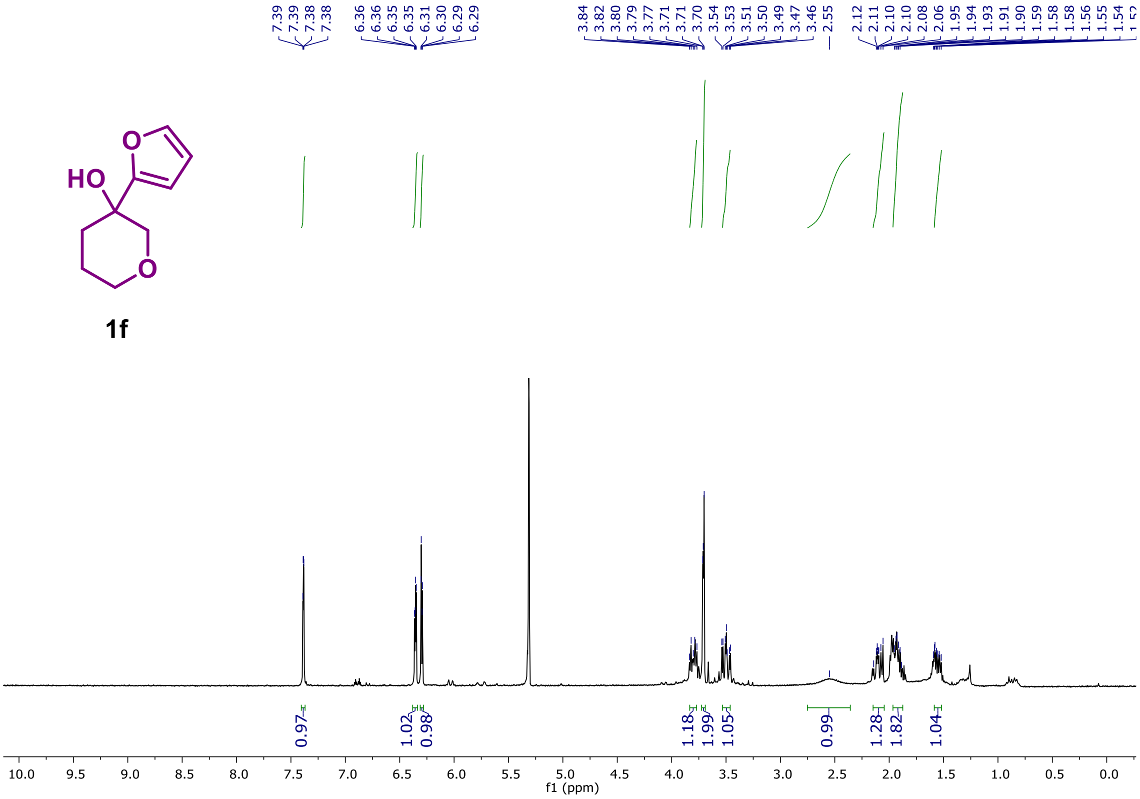

Figure S30. ^{1}H NMR spectrum (300 MHz, 298K, CD_2Cl_2) of **1f.**

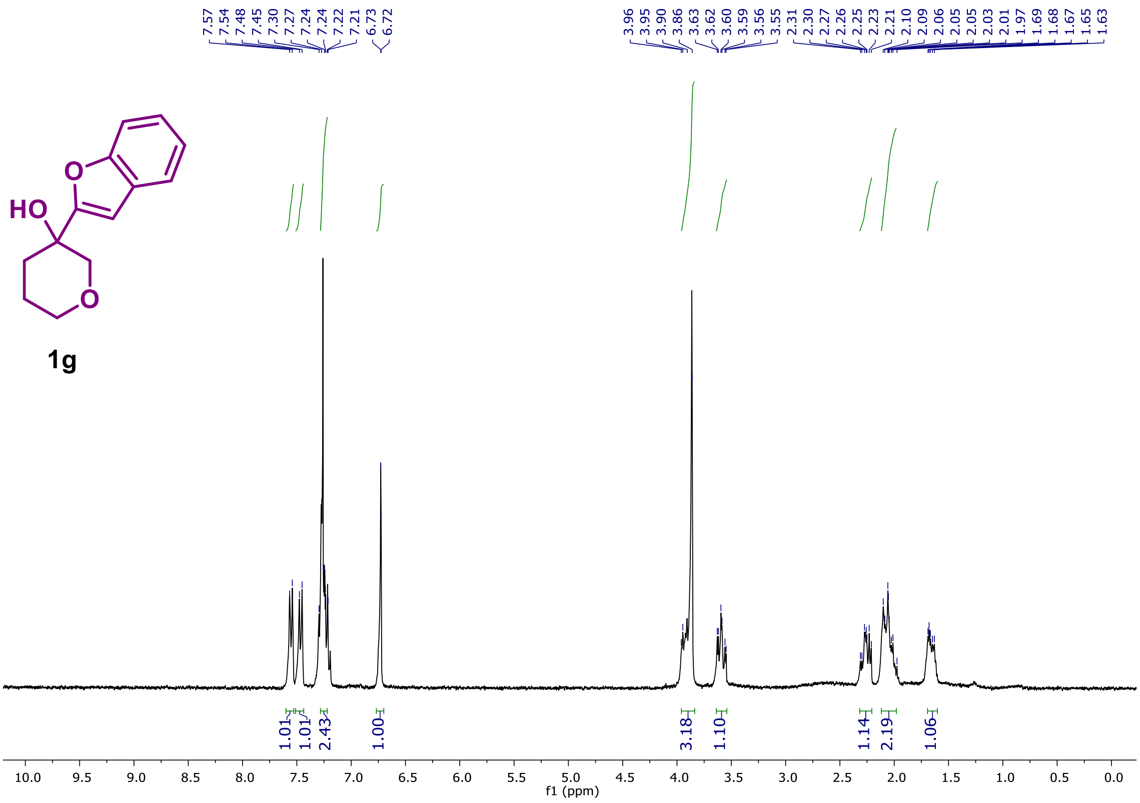

Figure S31. ^{1}H NMR spectrum (300 MHz, 298K, $CDCl_3$) of **1g.**

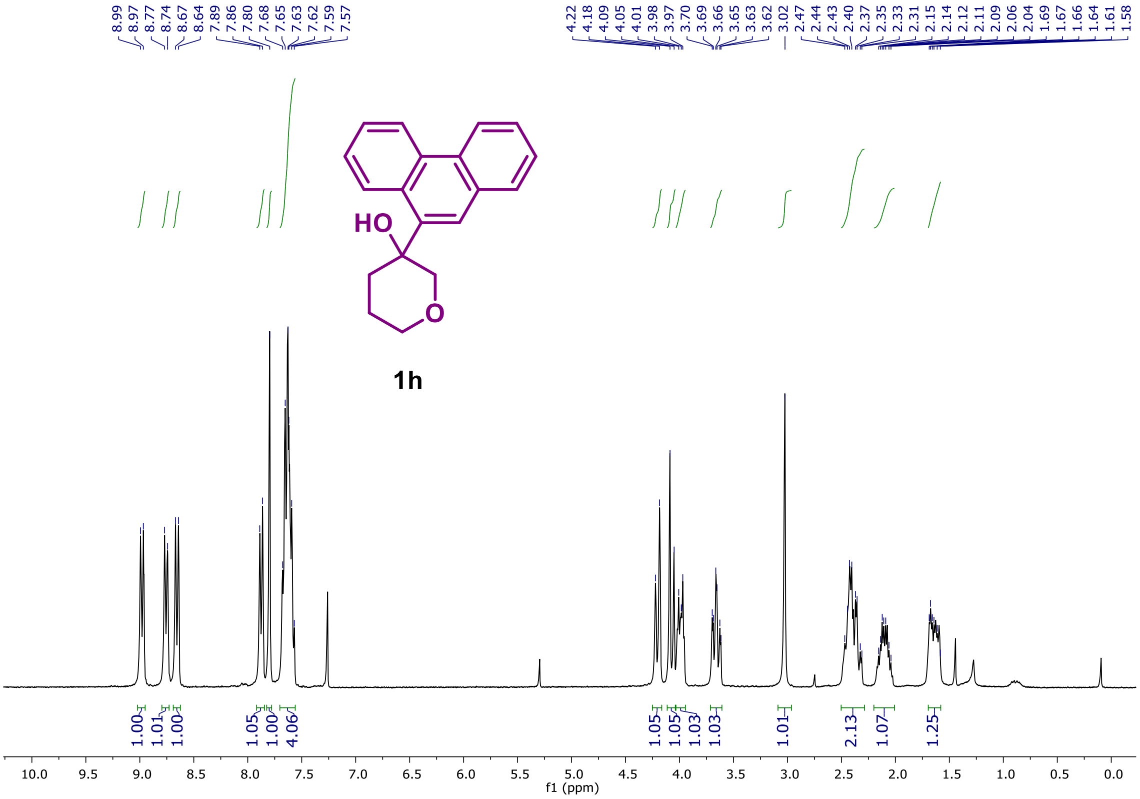

Figure S32. ^{1}H NMR spectrum (300 MHz, 298K, $CDCl_3$) of **1h.**

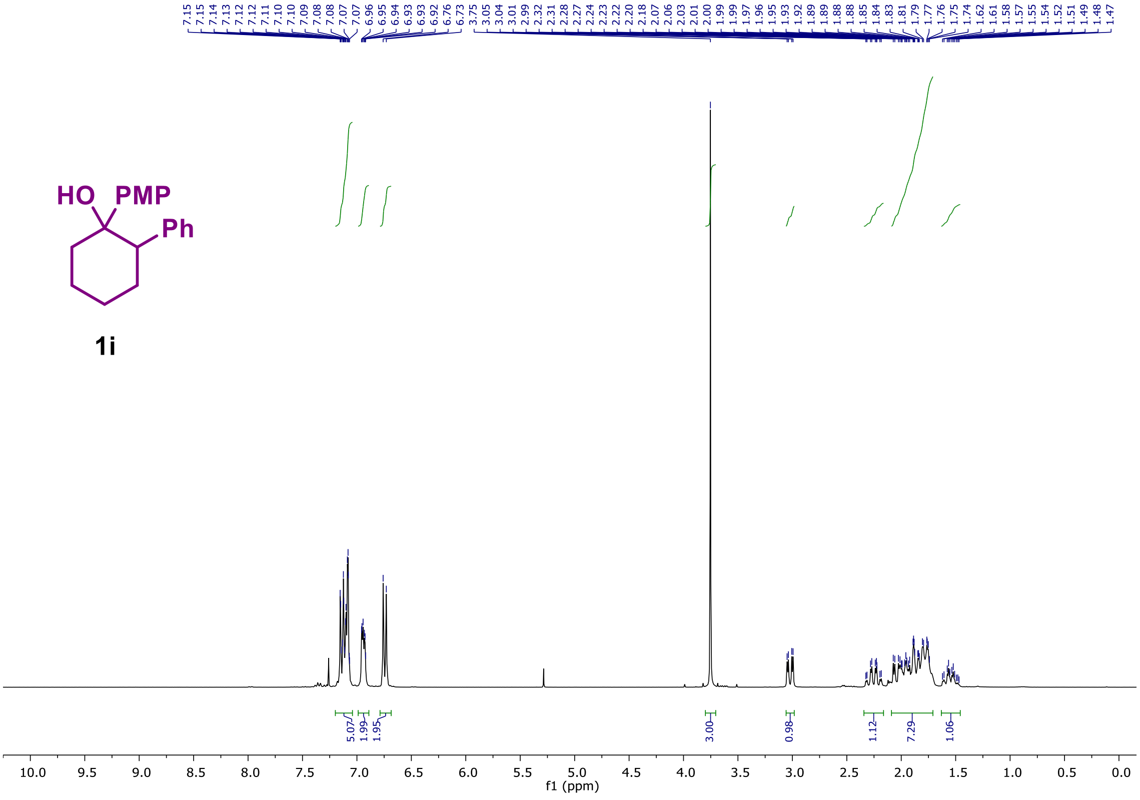

Figure S33. ^{1}H NMR spectrum (300 MHz, 298K, $CDCl_3$) of **1i.**

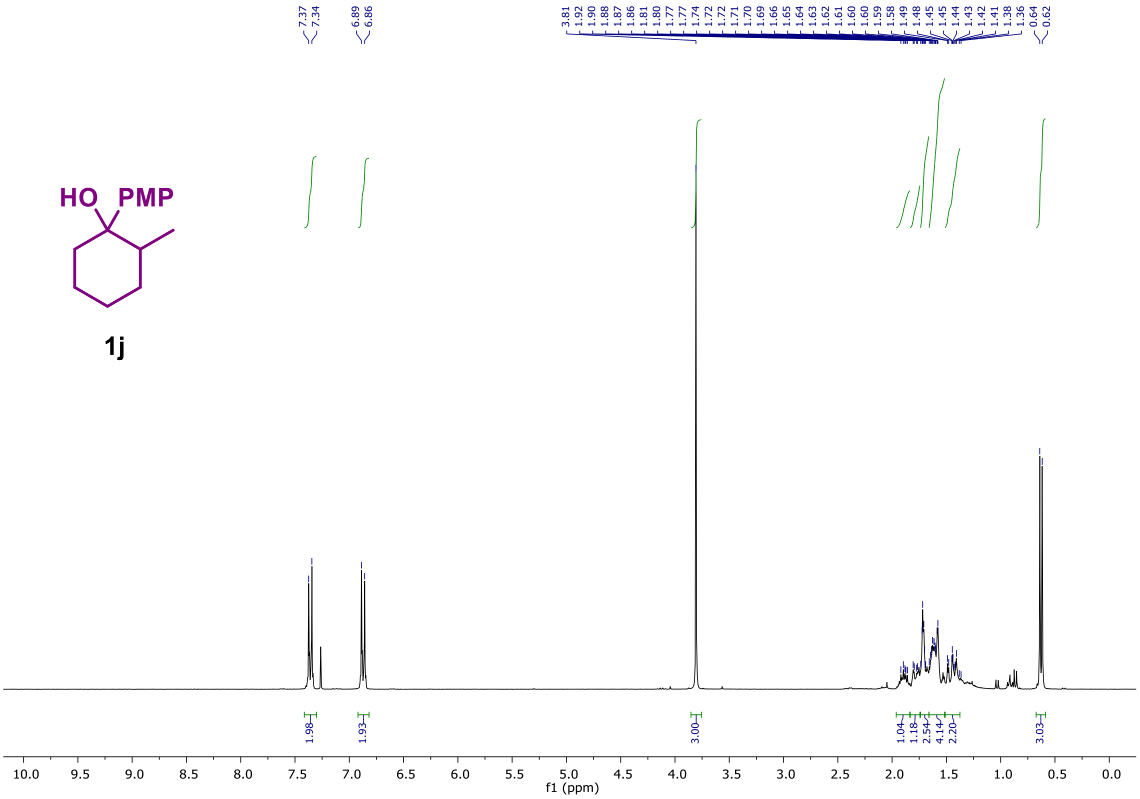

Figure S34. ^{1}H NMR spectrum (300 MHz, 298K, $CDCl_3$) of **1j.**

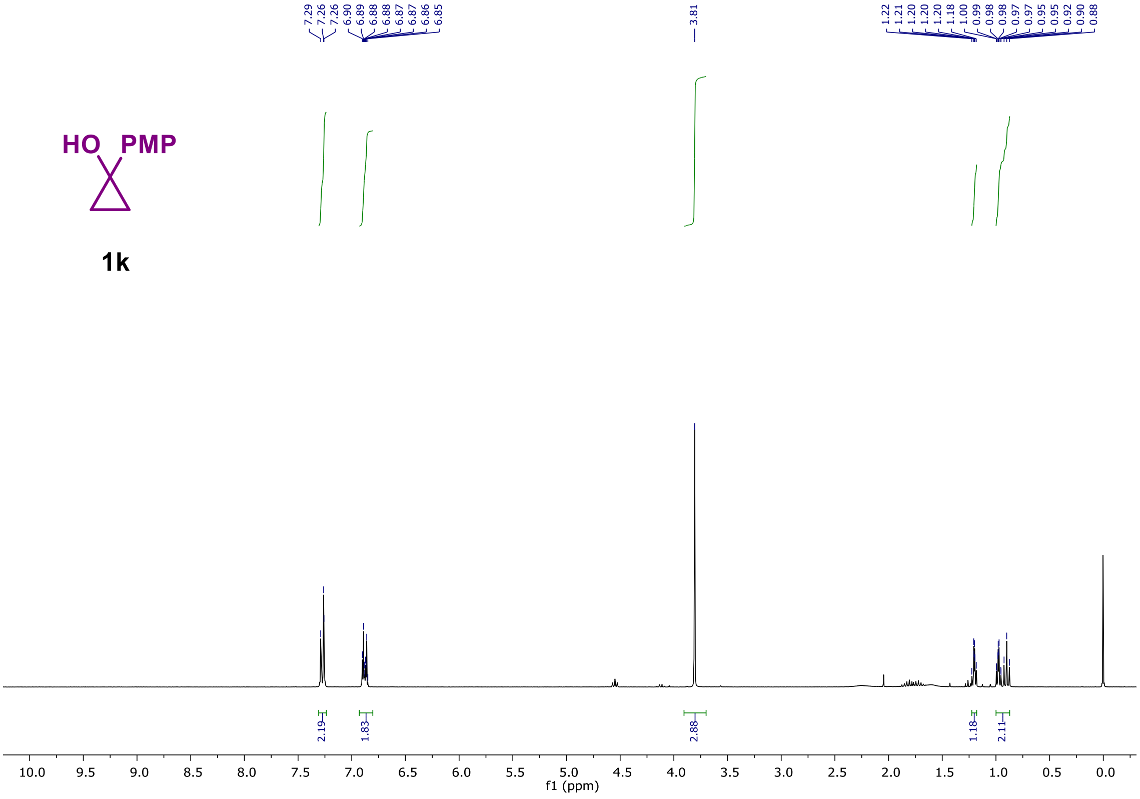

Figure S35. ^{1}H NMR spectrum (300 MHz, 298K, $CDCl_3$) of **1k.**

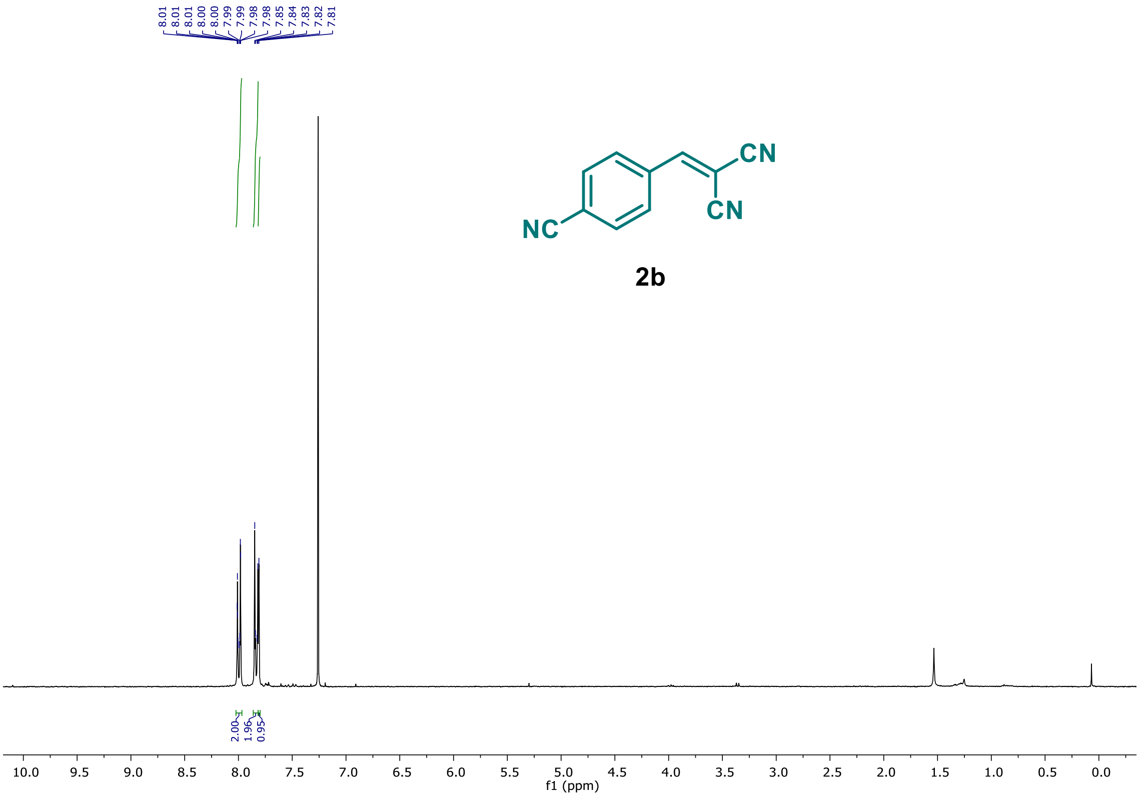

Figure S36. ^{1}H NMR spectrum (300 MHz, 298K, $CDCl_3$) of **2b.**

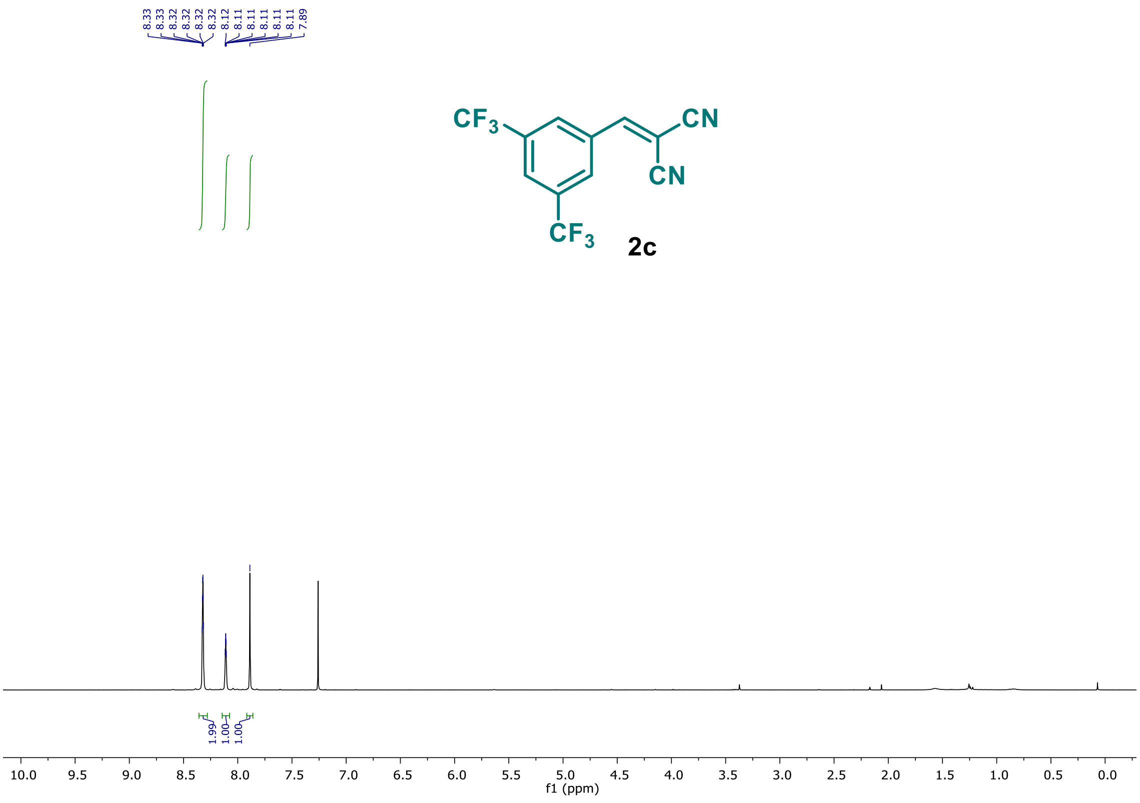

Figure S37. ^{1}H NMR spectrum (300 MHz, 298K, $CDCl_3$) of **2c.**

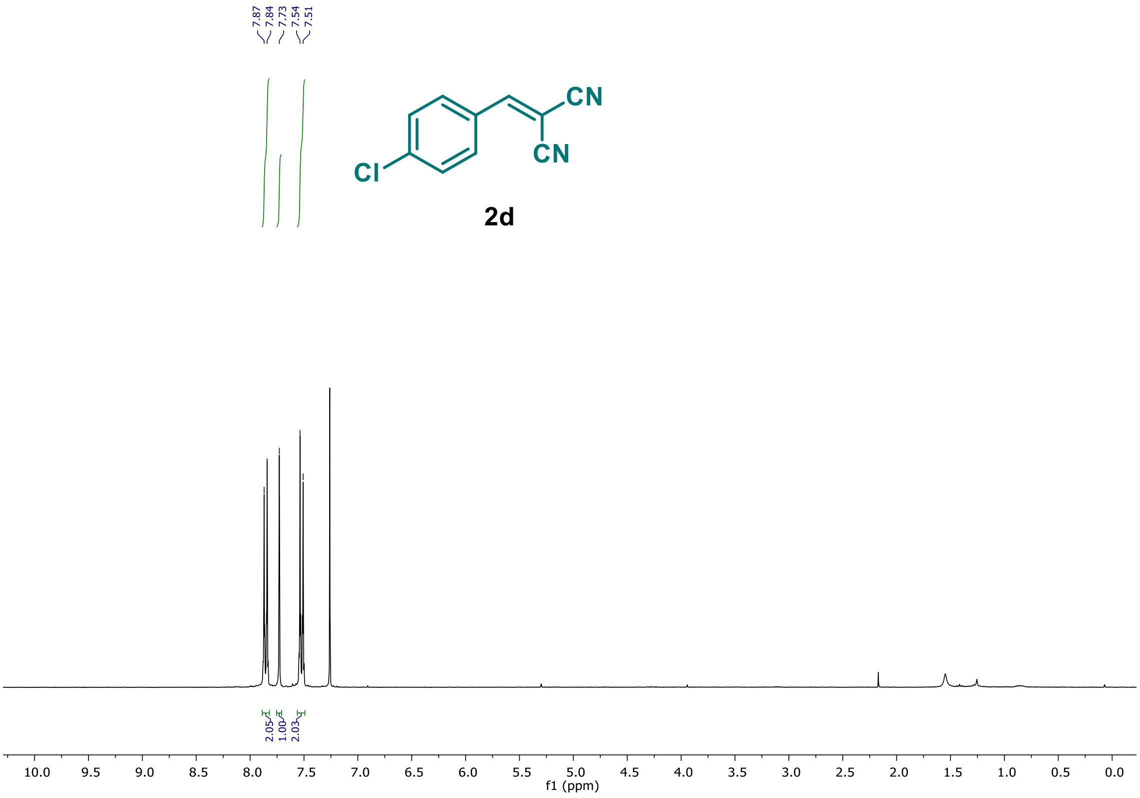

Figure S38. ^{1}H NMR spectrum (300 MHz, 298K, $CDCl_3$) of **2d.**

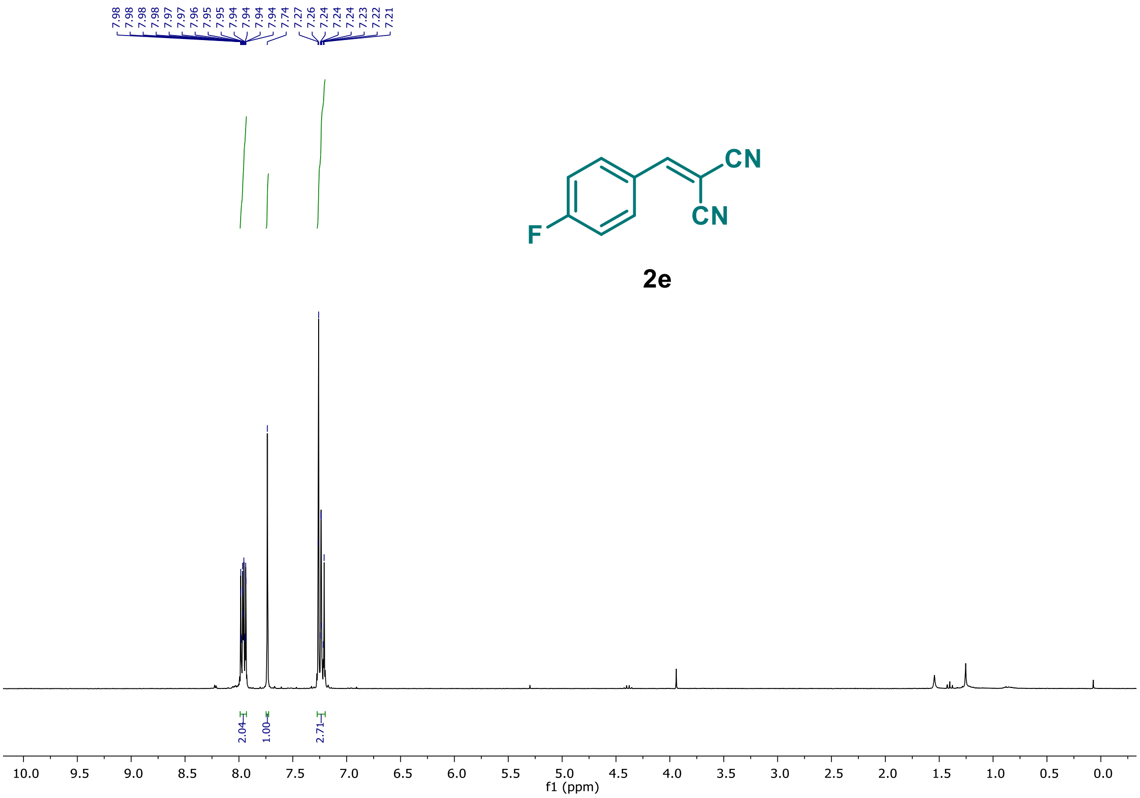

Figure S39. ^{1}H NMR spectrum (300 MHz, 298K, $CDCl_3$) of **2e.**

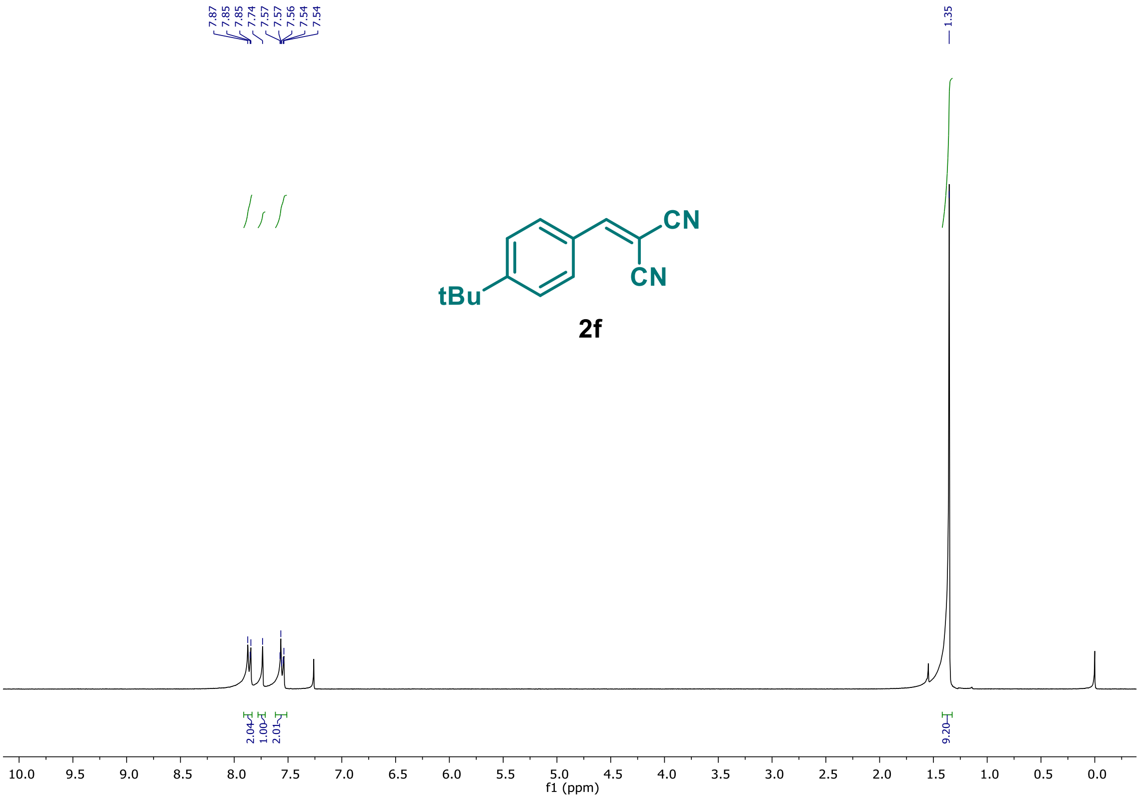

Figure S40. ^{1}H NMR spectrum (300 MHz, 298K, $CDCl_3$) of **2f.**

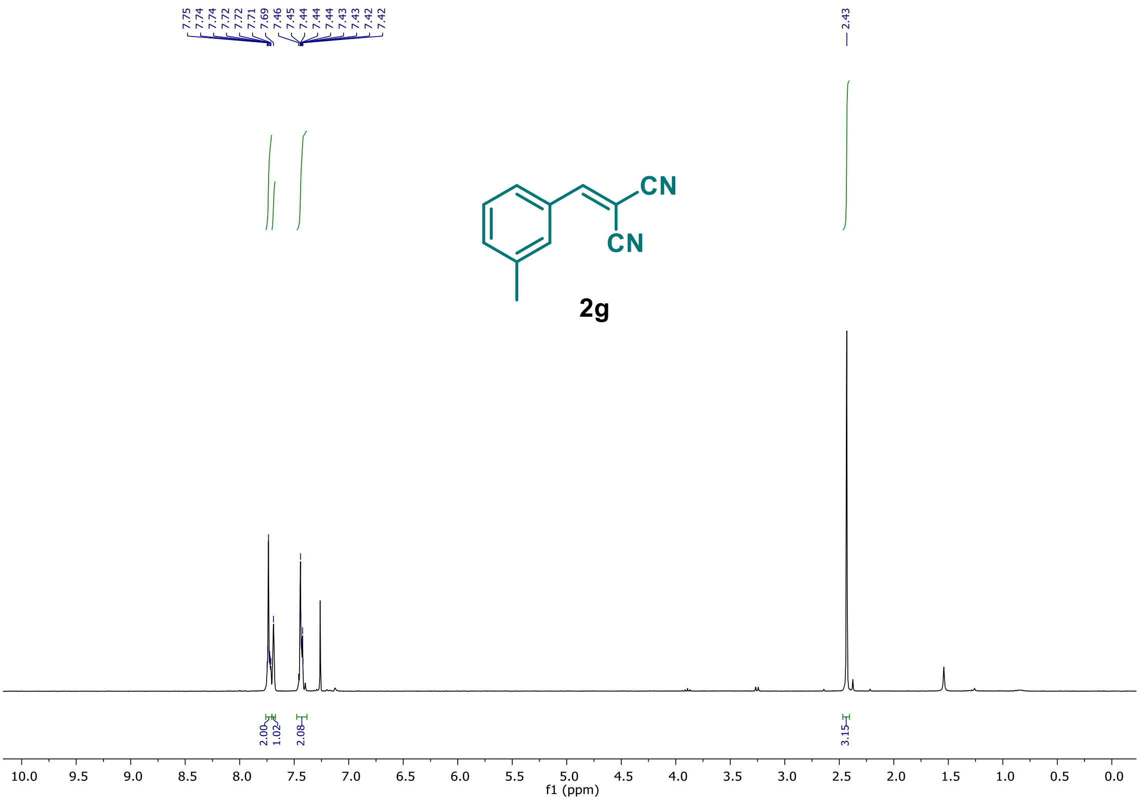

Figure S41. ^{1}H NMR spectrum (300 MHz, 298K, $CDCl_3$) of **2g.**

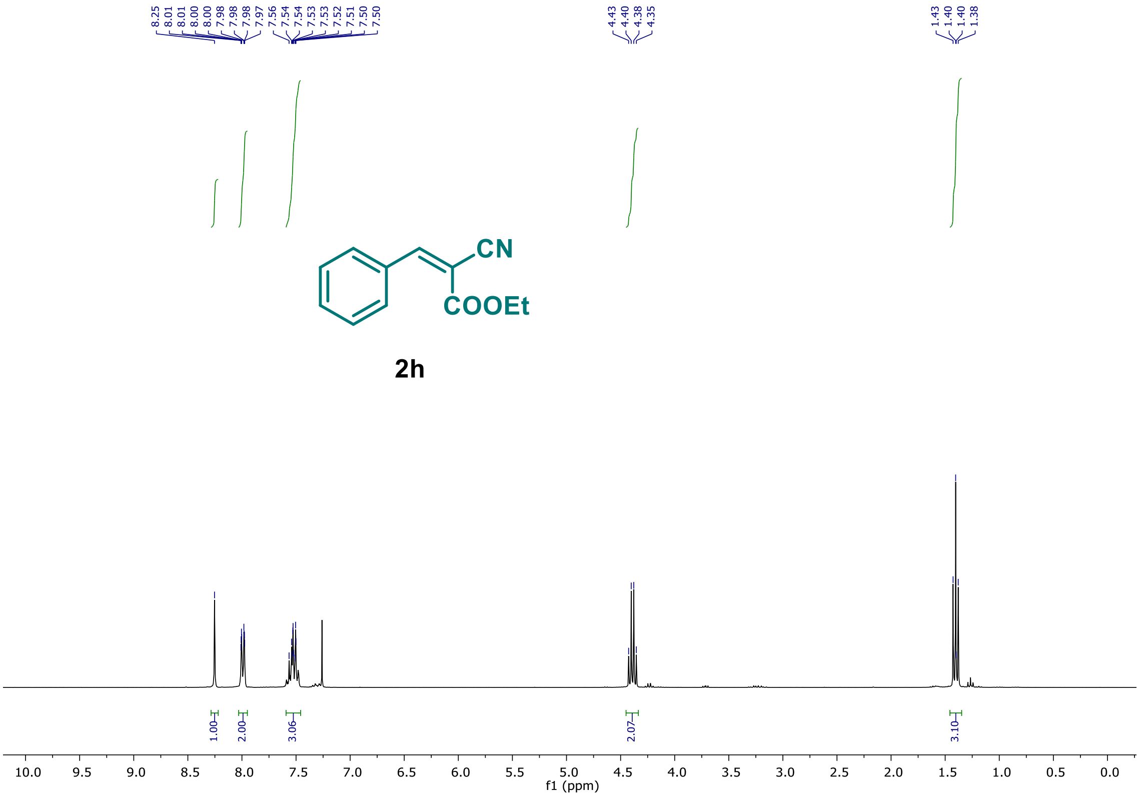

2h

Figure S42. ^{1}H NMR spectrum (300 MHz, 298K, $CDCl_3$) of **2h.**

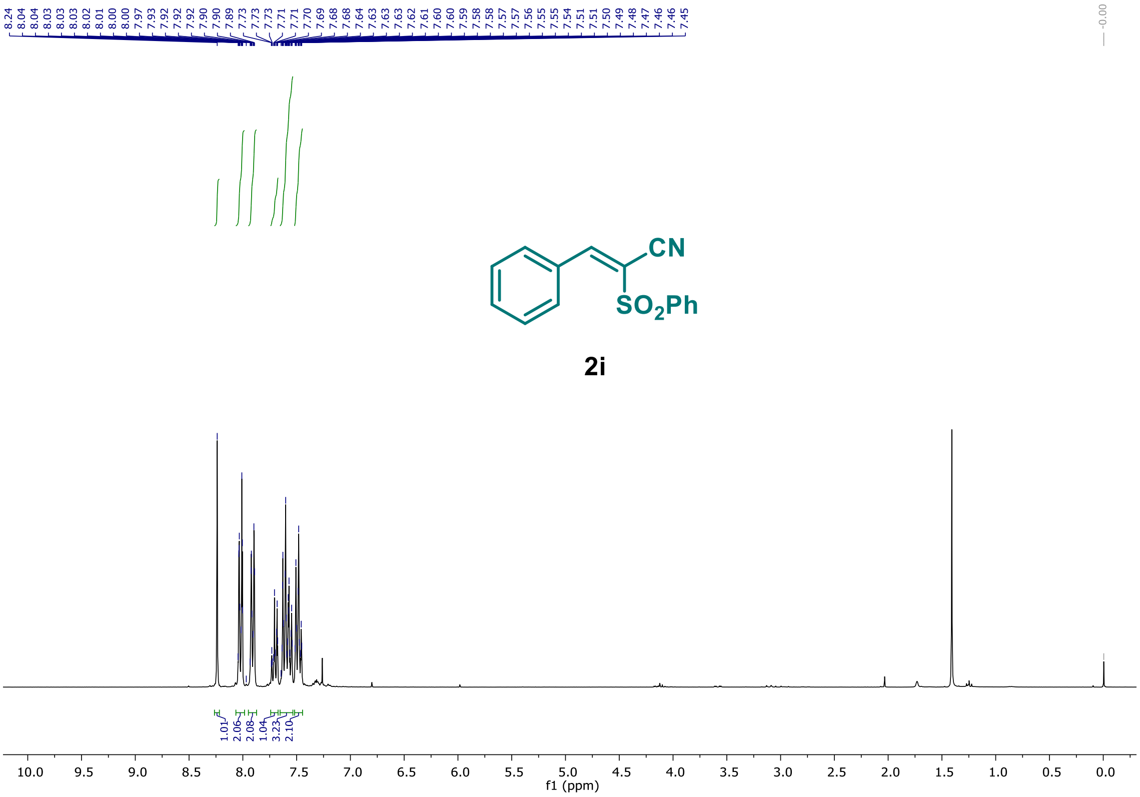

2i

Figure S43. ^{1}H NMR spectrum (300 MHz, 298K, $CDCl_3$) of **2i.**

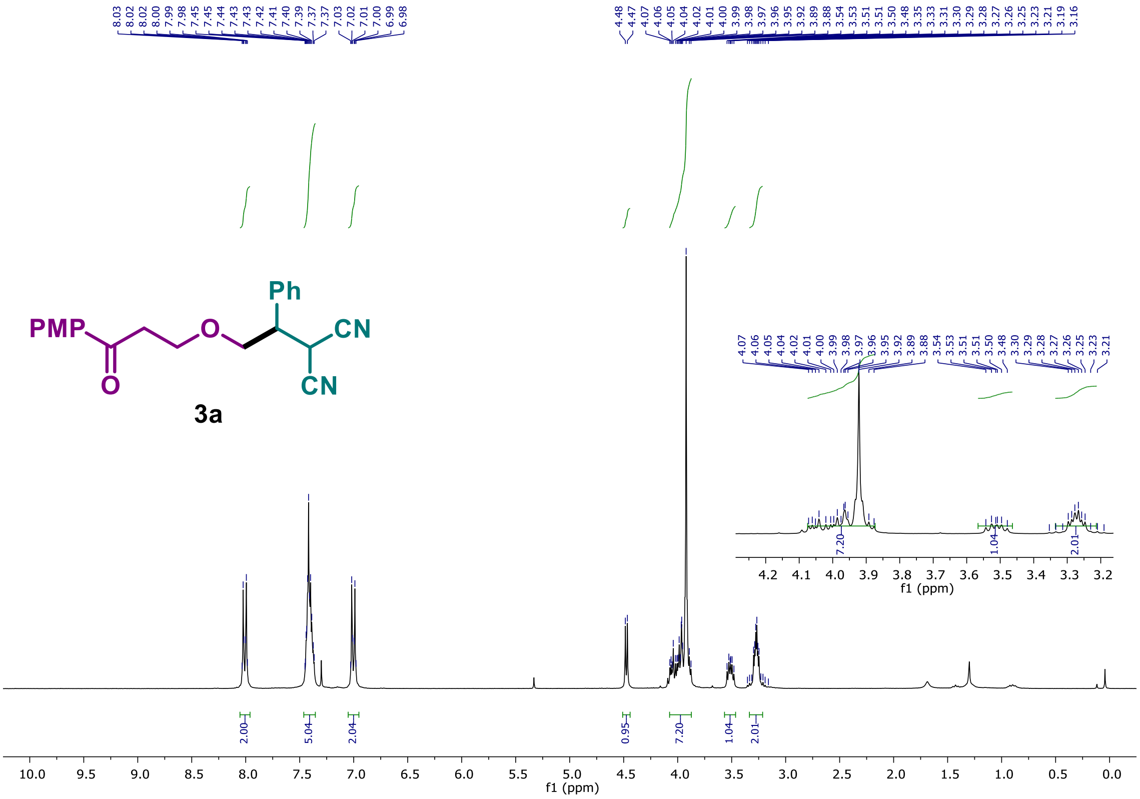

Figure S44. ^{1}H NMR spectrum (300 MHz, 298K, $CDCl_3$) of **3a.**

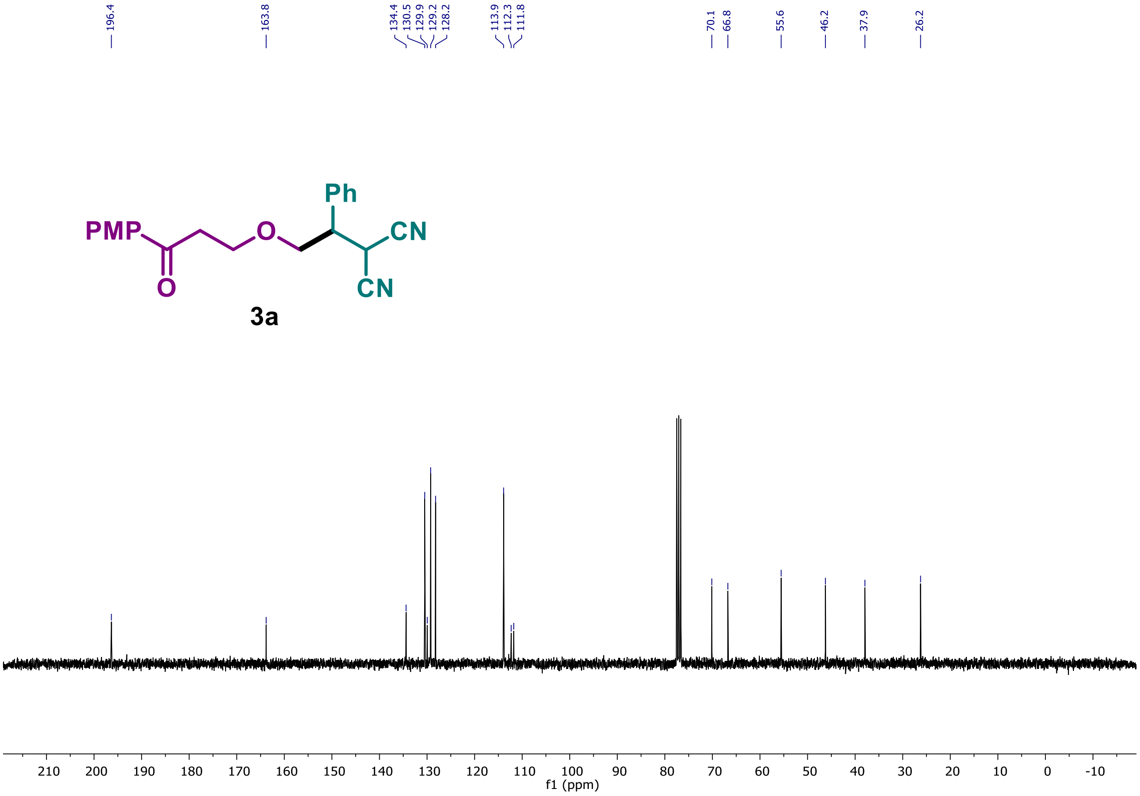

Figure S45. ^{13}C NMR spectrum (75 MHz, 298K, $CDCl_3$) of **3a.**

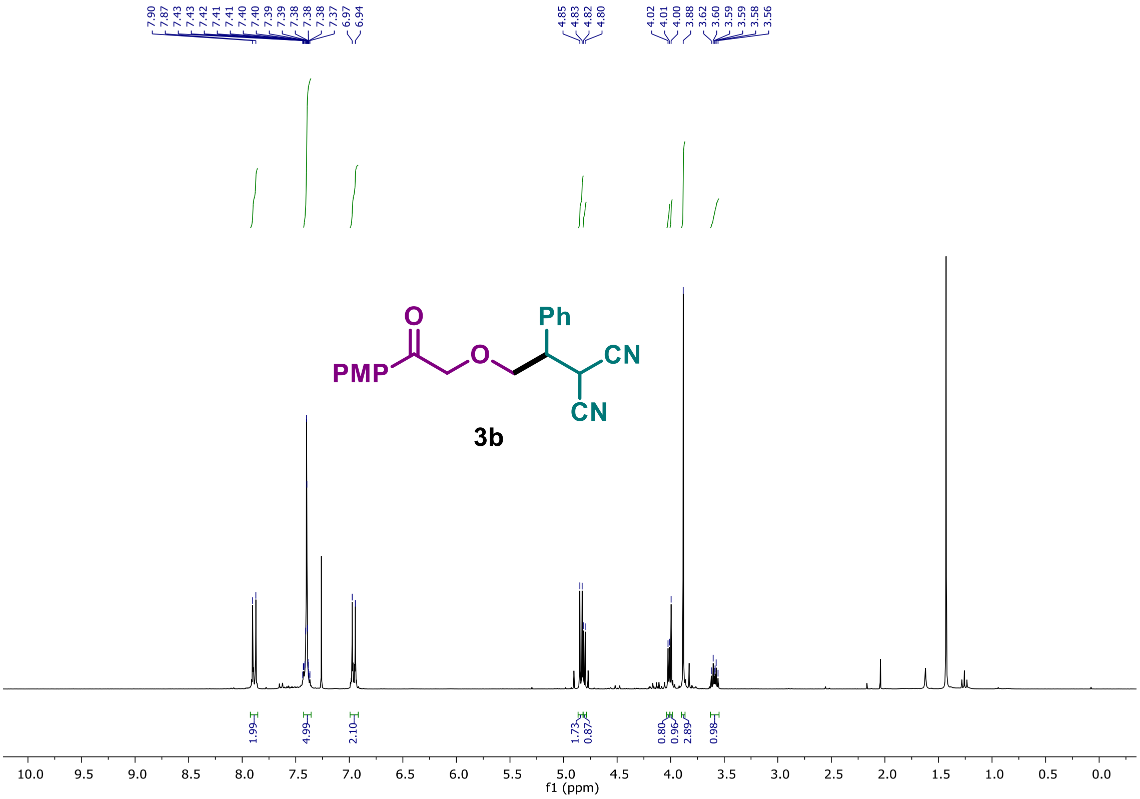

Figure S46. ^{1}H NMR spectrum (300 MHz, 298K, $CDCl_3$) of **3b.**

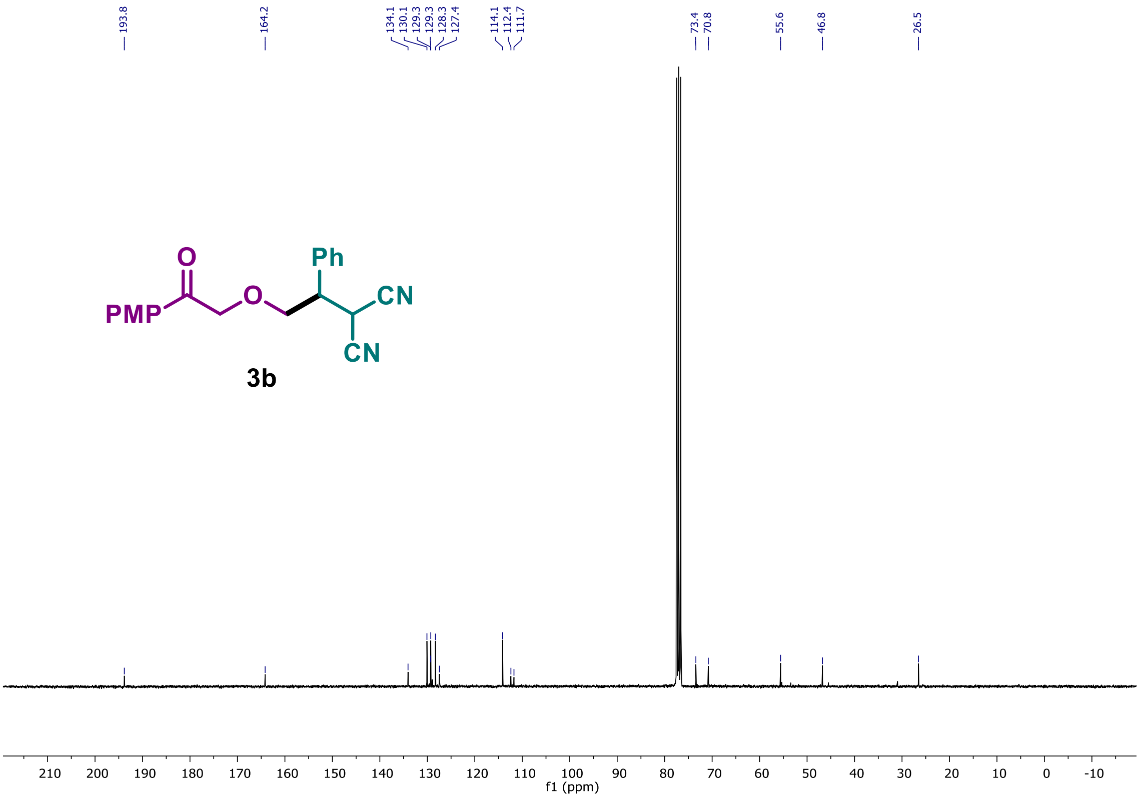

Figure S47. ^{13}C NMR spectrum (75 MHz, 298K, $CDCl_3$) of **3b.**

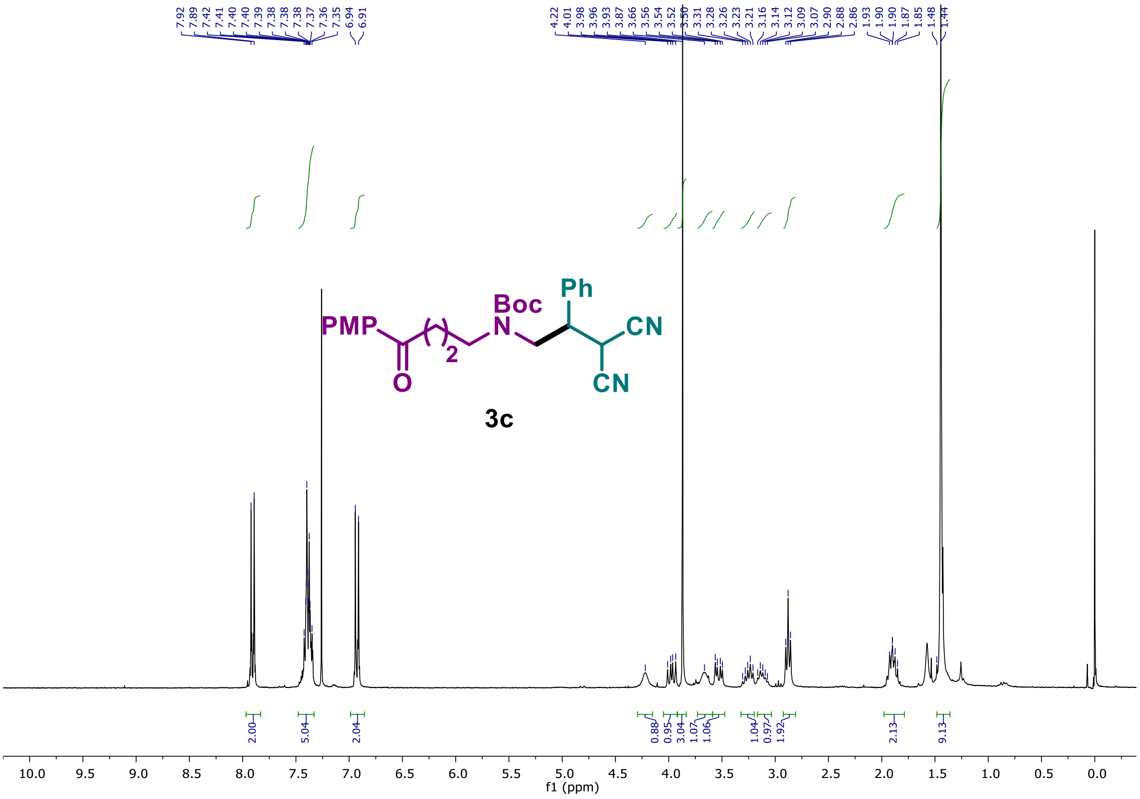

Figure S48. ^{1}H NMR spectrum (300 MHz, 298K, $CDCl_3$) of **3c.**

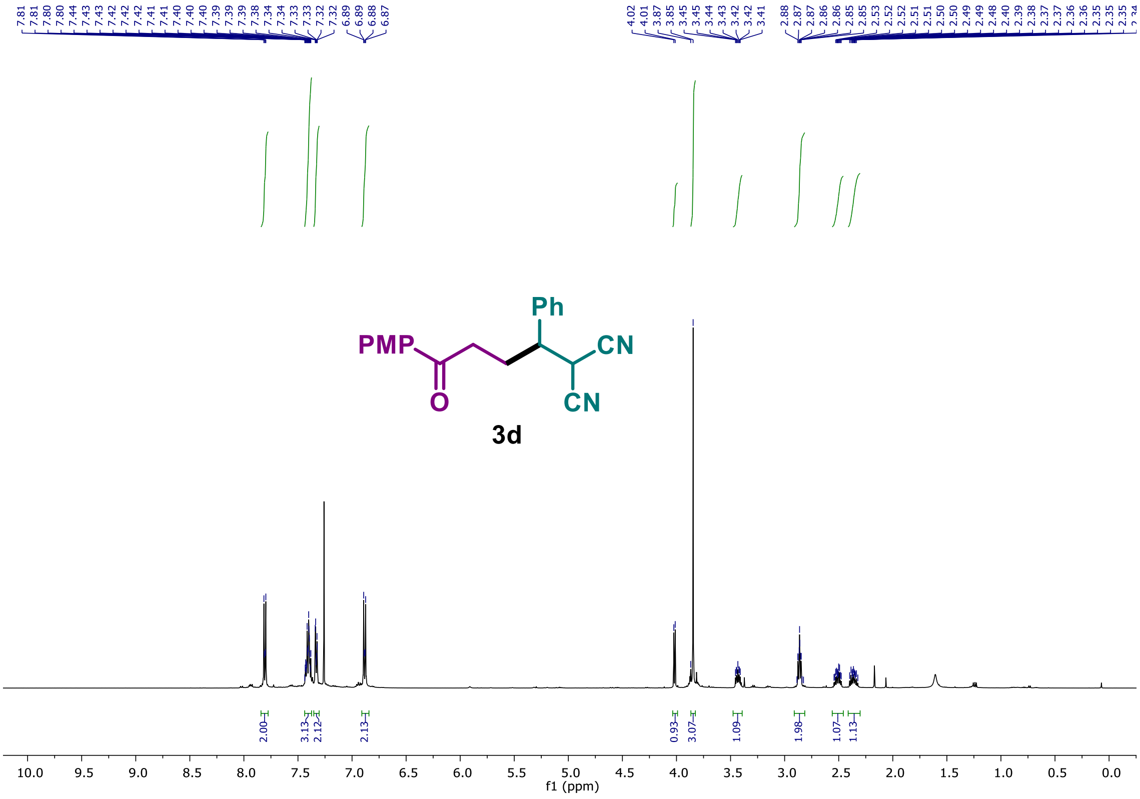

Figure S49. ^{13}C NMR spectrum (75 MHz, 298K, $CDCl_3$) of **3d..**

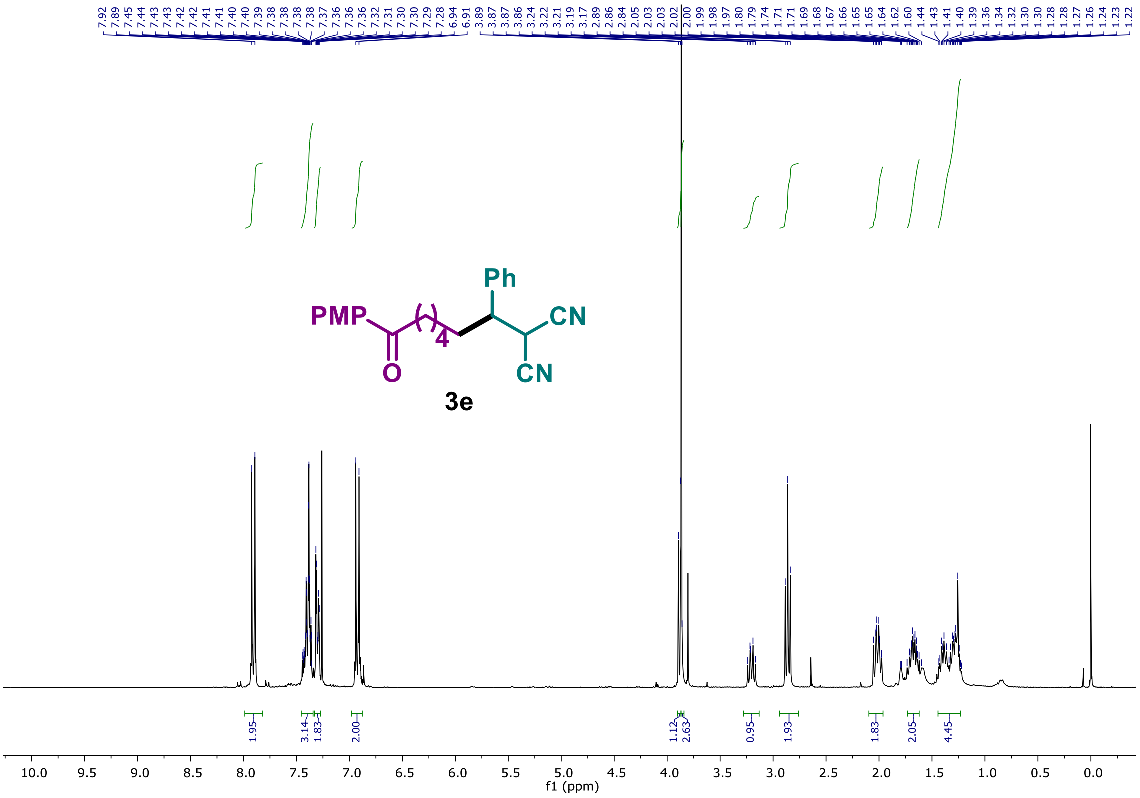

Figure S50. ^{1}H NMR spectrum (300 MHz, 298K, $CDCl_3$) of **3e.**

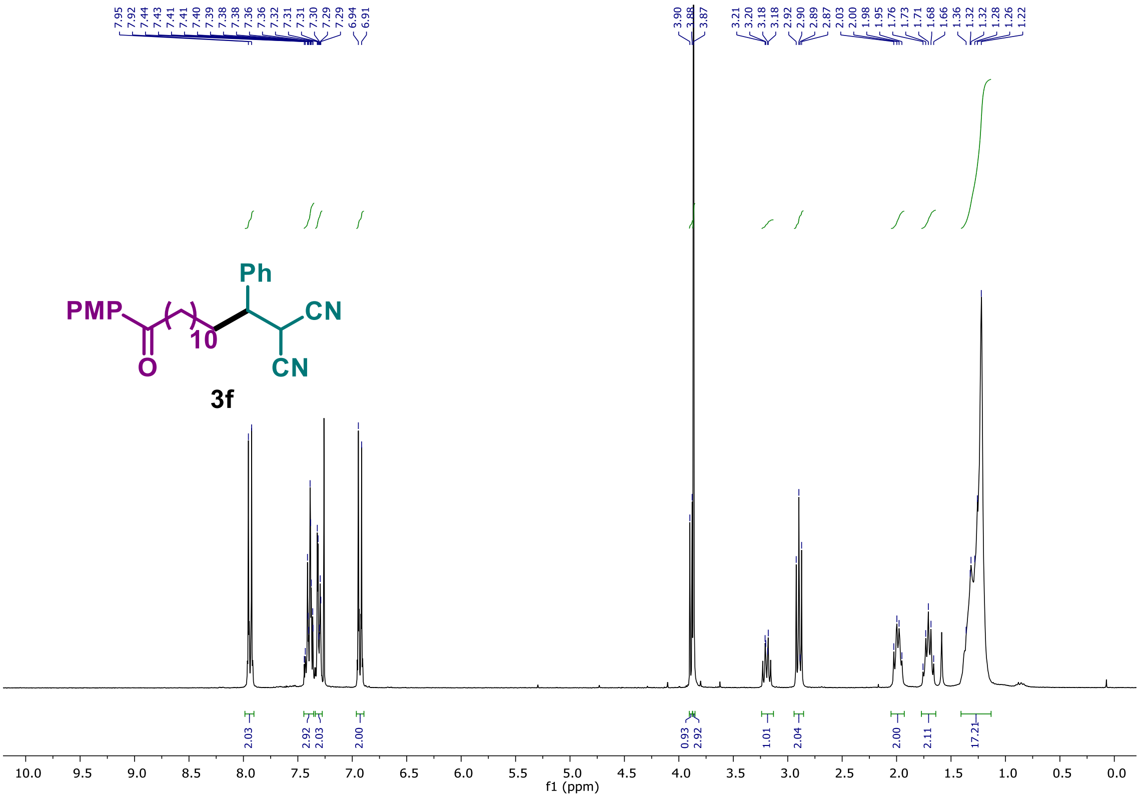

Figure S51. ^{1}H NMR spectrum (300 MHz, 298K, $CDCl_3$) of **3f.**

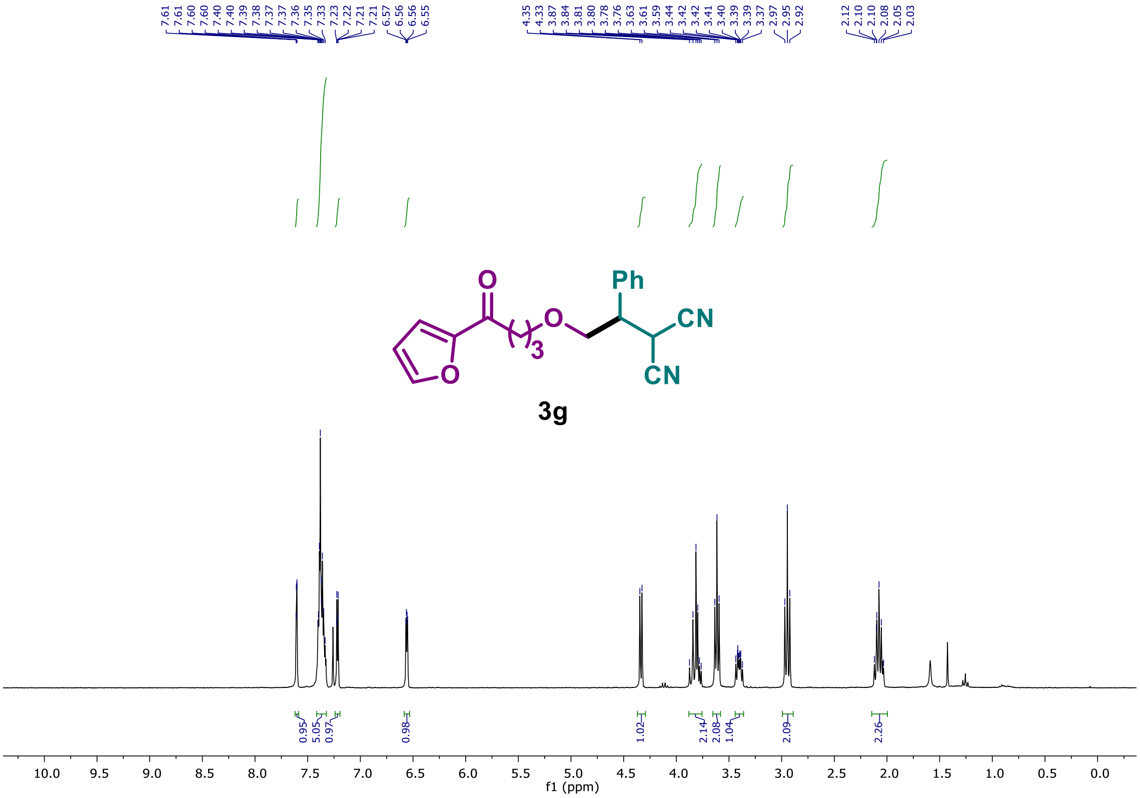

Figure S52. ^{1}H NMR spectrum (300 MHz, 298K, $CDCl_3$) of **3g.**

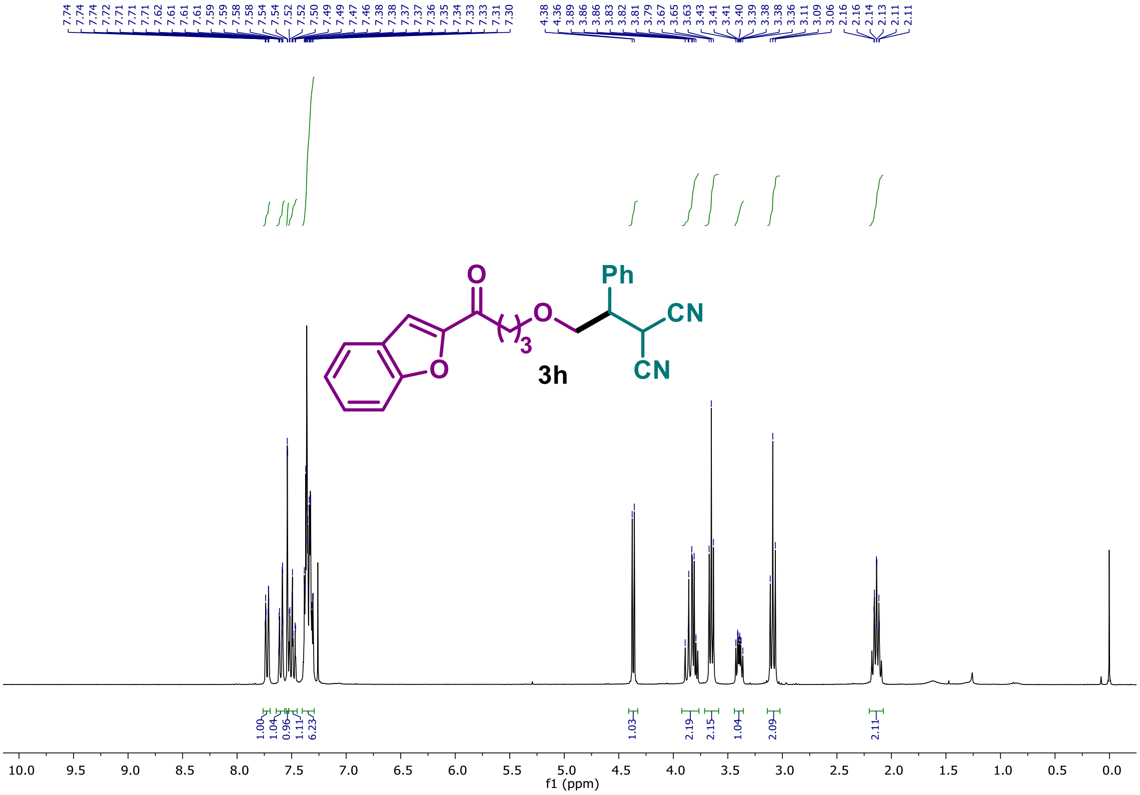

Figure S53. ^{1}H NMR spectrum (300 MHz, 298K, $CDCl_3$) of **3h.**

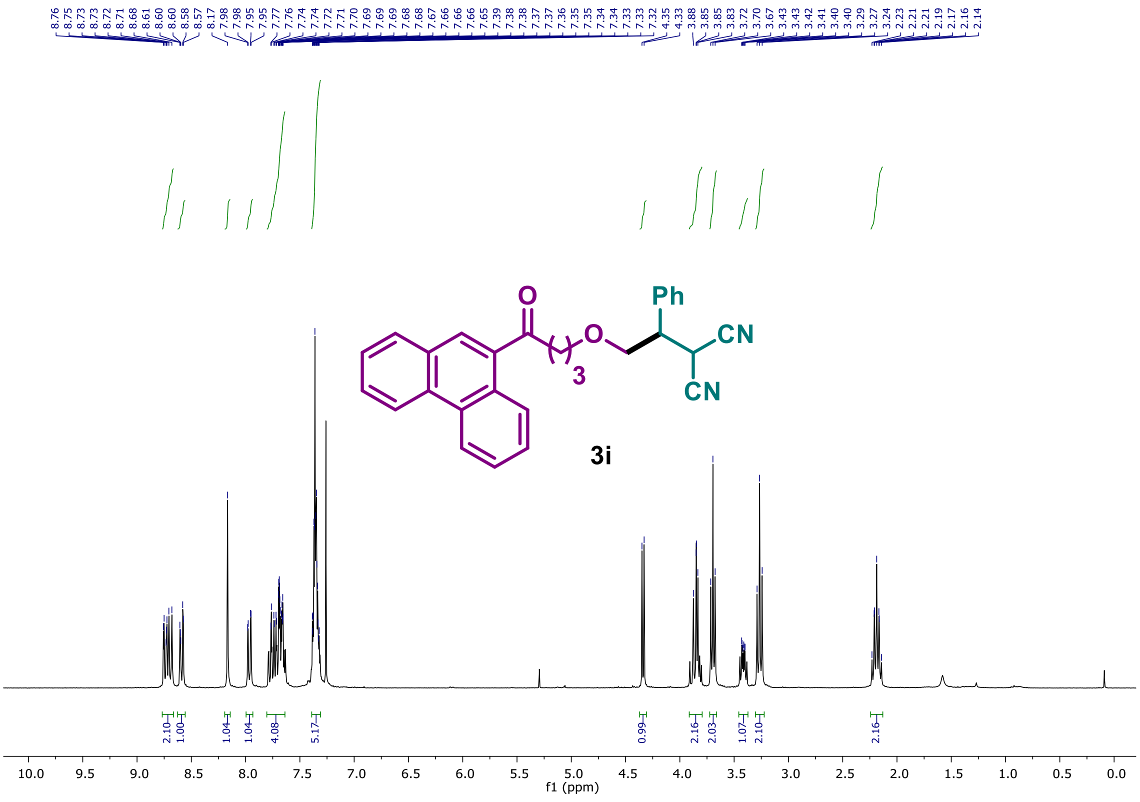

Figure S54. ^{1}H NMR spectrum (300 MHz, 298K, $CDCl_3$) of **3i.**

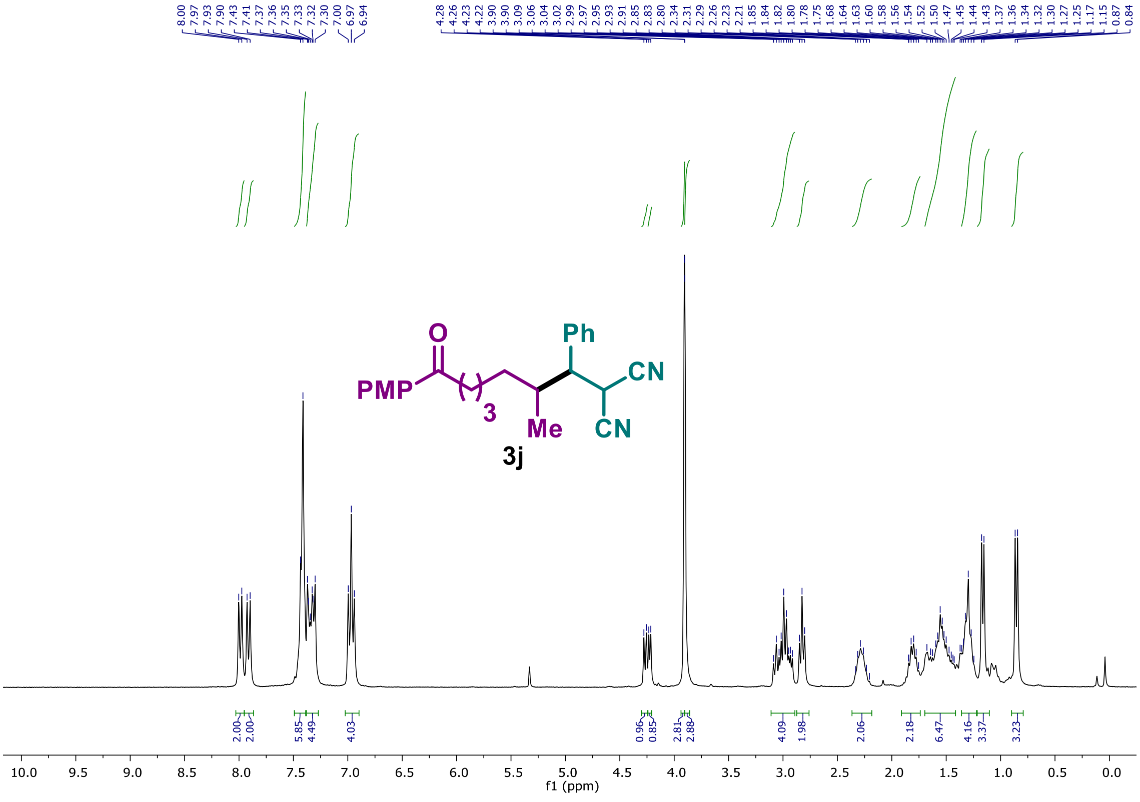

Figure S55. ^{13}C NMR spectrum (75 MHz, 298K, $CDCl_3$) of **3j.**

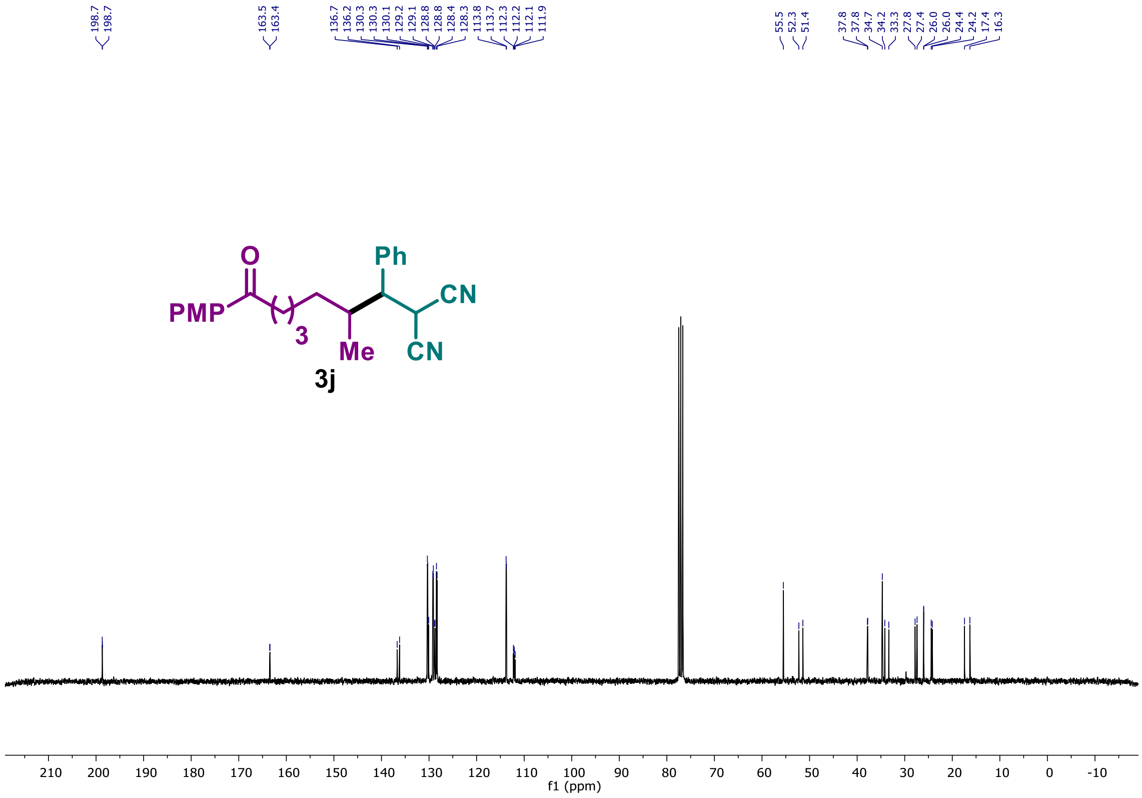

Figure S56. ^{13}C NMR spectrum (75 MHz, 298K, $CDCl_3$) of **3j.**

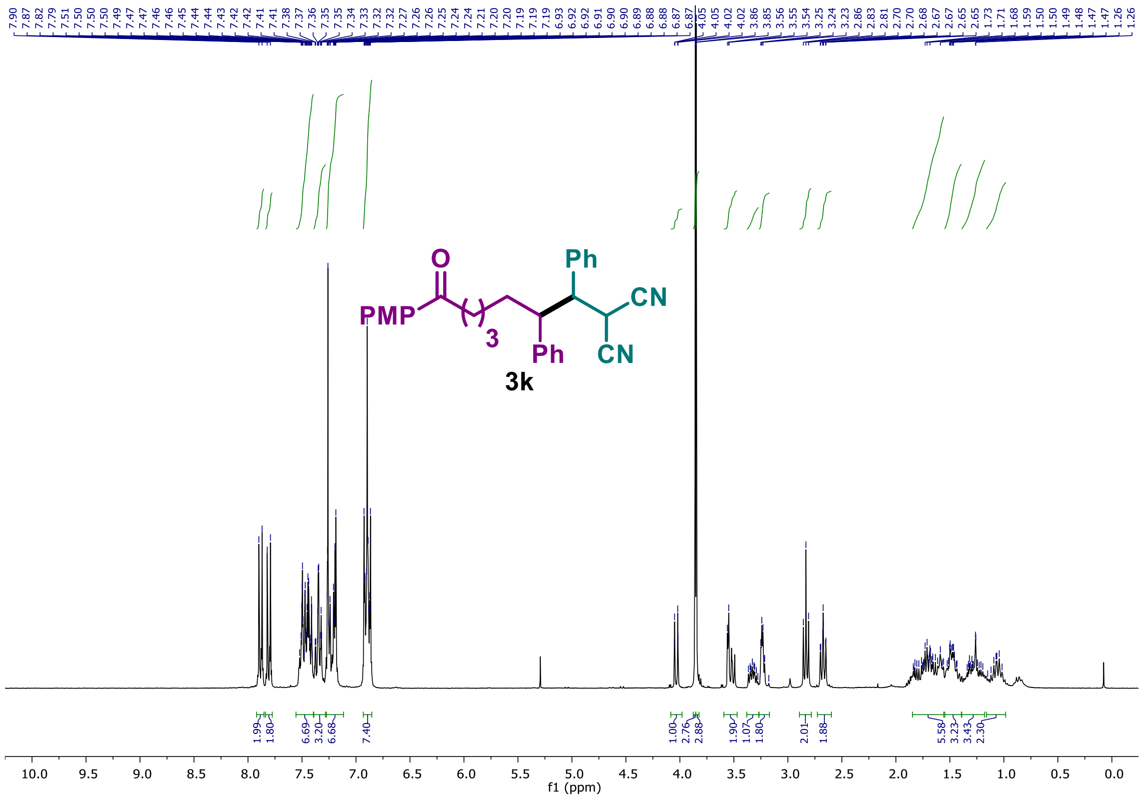

Figure S57. ^{1}H NMR spectrum (300 MHz, 298K, $CDCl_3$) of **3k.**

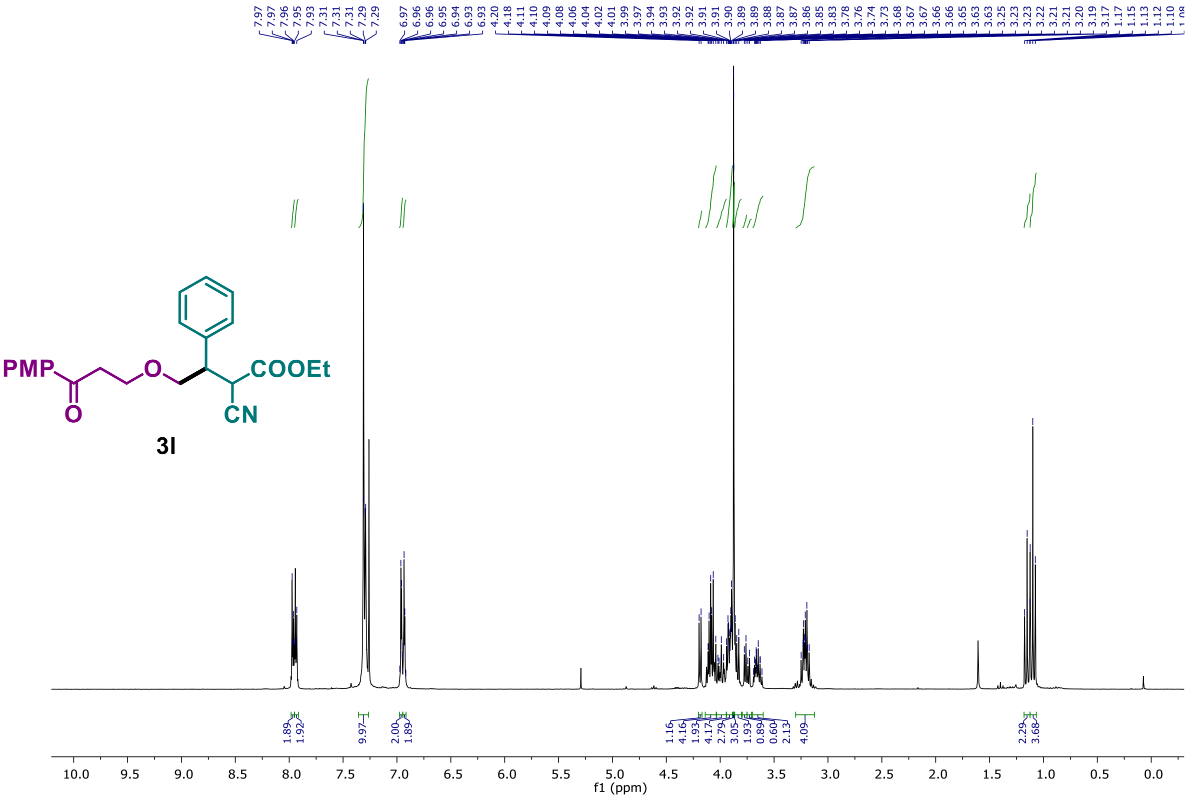

Figure S58.. ^{1}H NMR spectrum (300 MHz, 298K, $CDCl_3$) of **3l.**

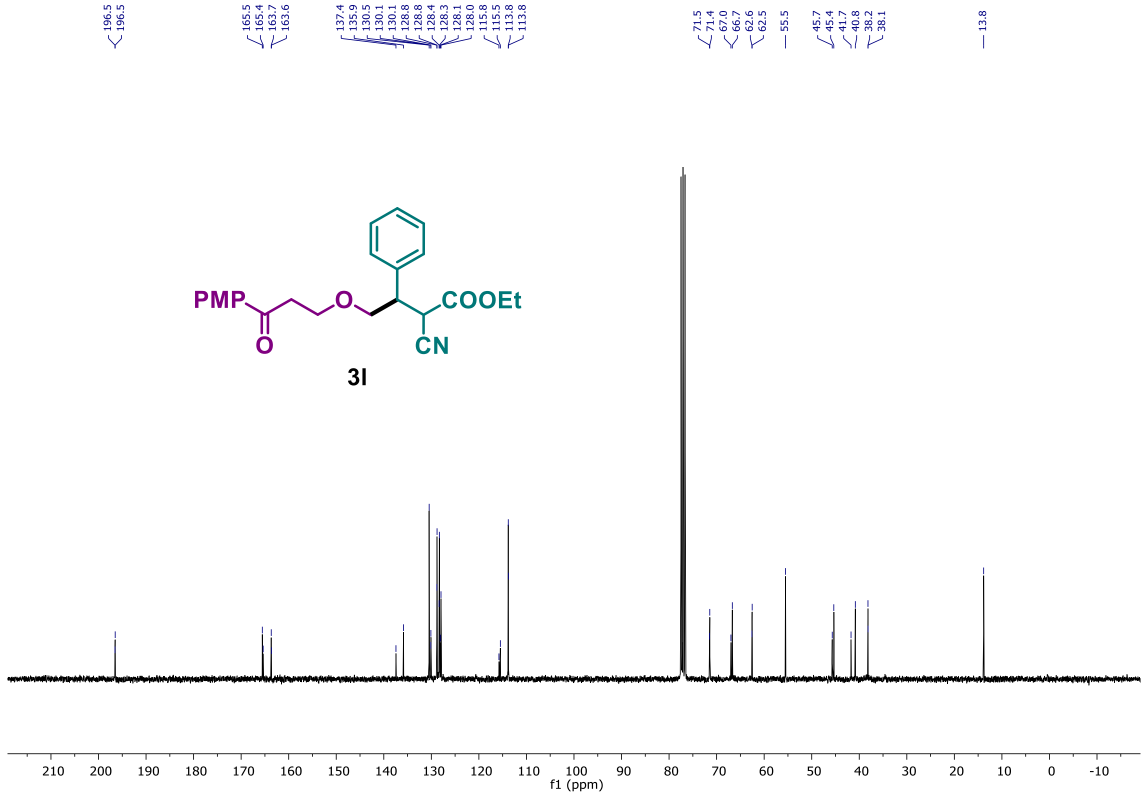

Figure S59. ^{13}C NMR spectrum (75 MHz, 298K, $CDCl_3$) of **3l.**

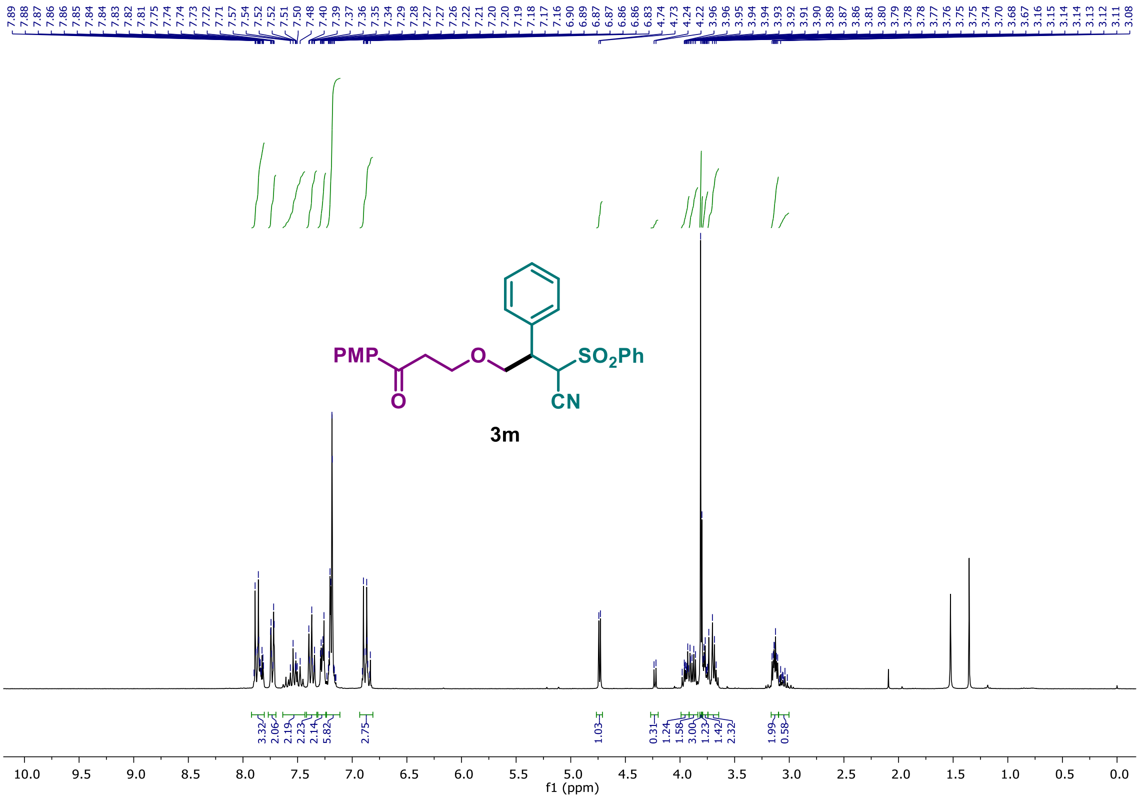

Figure S60. ^{1}H NMR spectrum (300 MHz, 298K, $CDCl_3$) of **3m.**

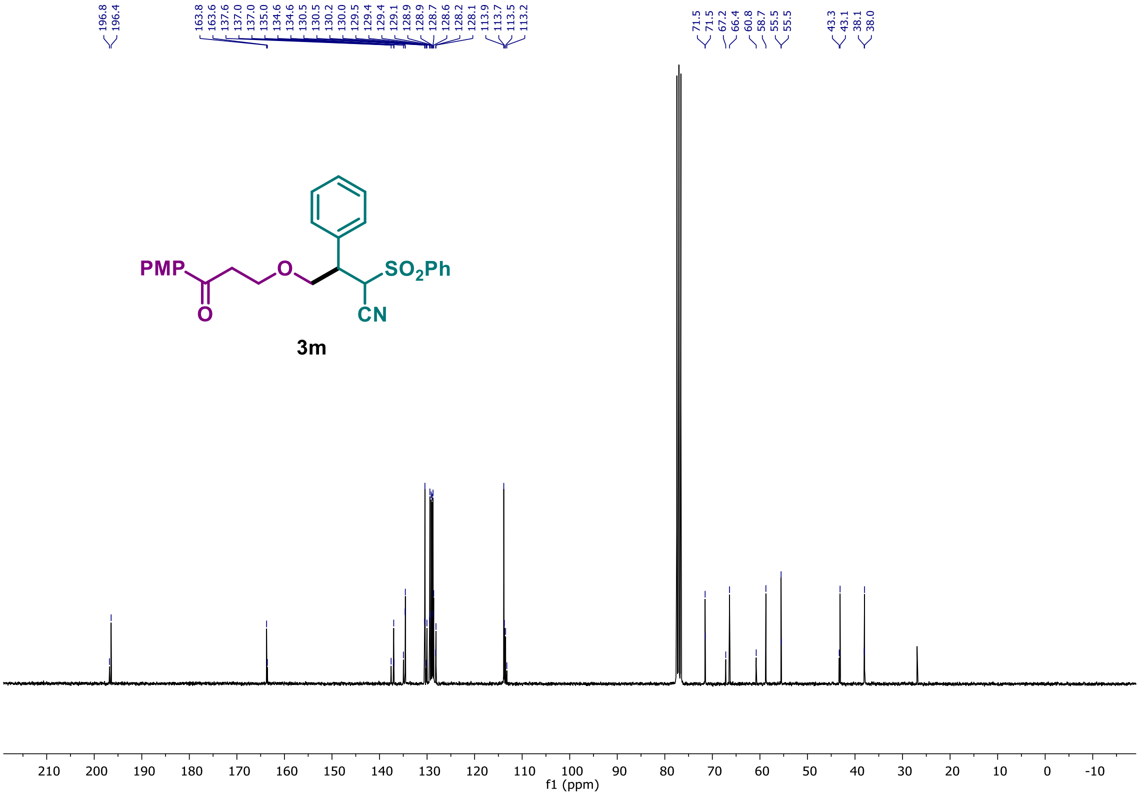

Figure S61. ^{13}C NMR spectrum (75 MHz, 298K, $CDCl_3$) of **3m.**

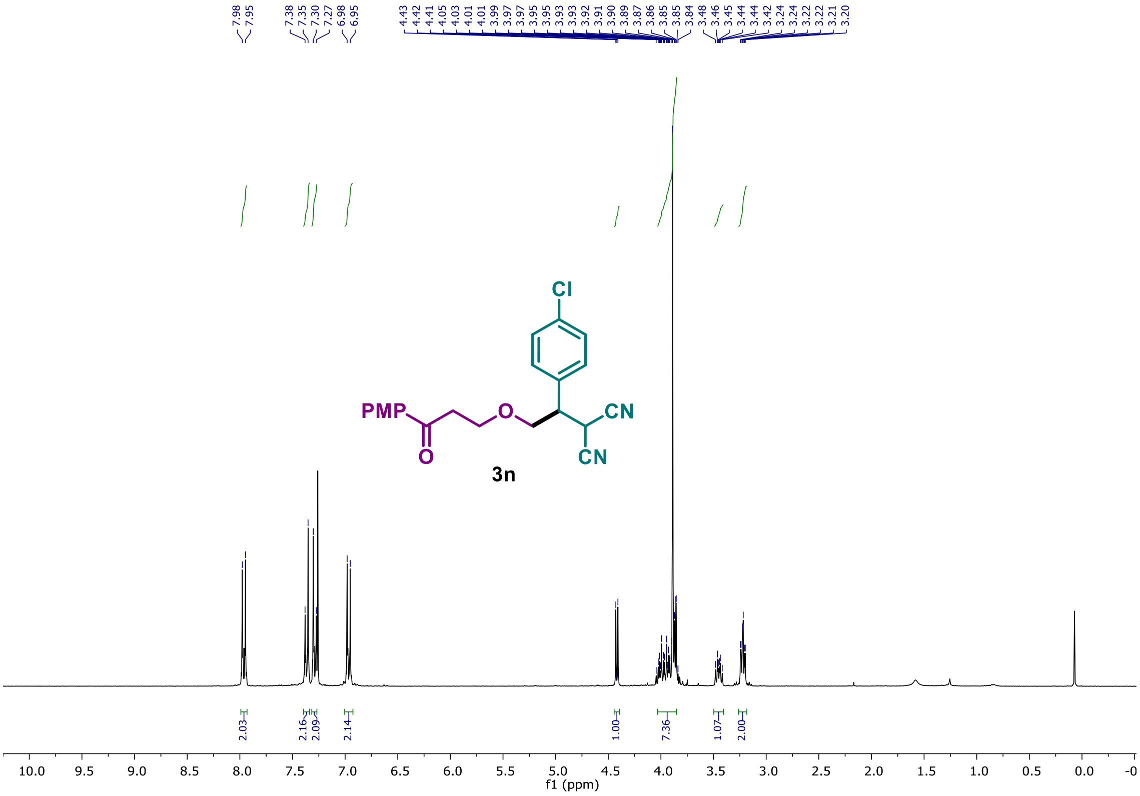

Figure S62. ^{1}H NMR spectrum (300 MHz, 298K, $CDCl_3$) of **3n.**

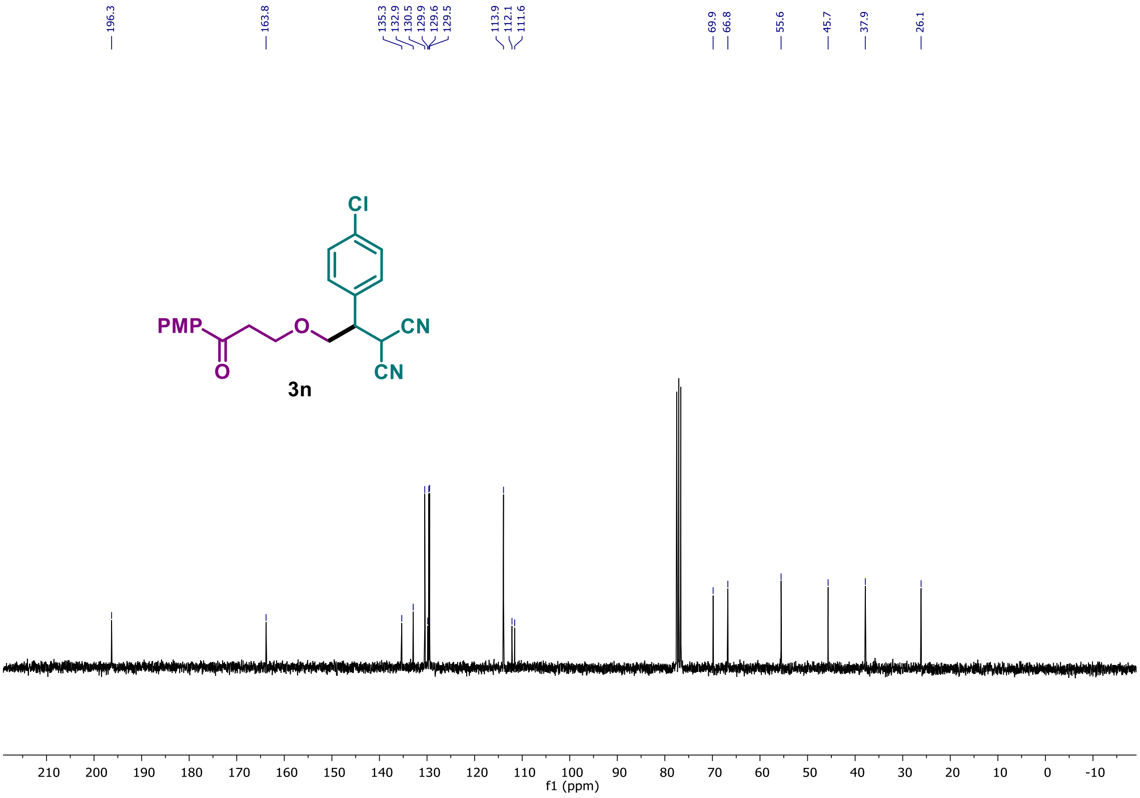

Figure S63. ^{13}C NMR spectrum (75 MHz, 298K, $CDCl_3$) of **3n.**

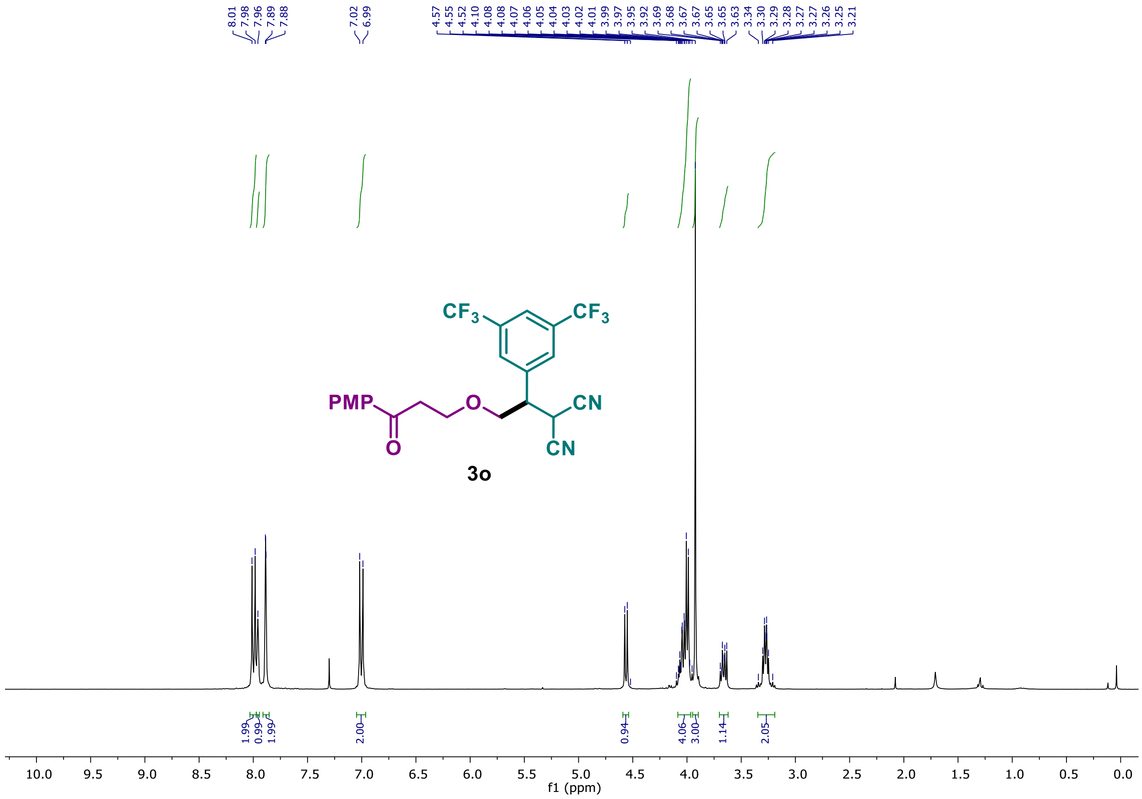

Figure S64. ^{1}H NMR spectrum (300 MHz, 298K, $CDCl_3$) of **3o.**

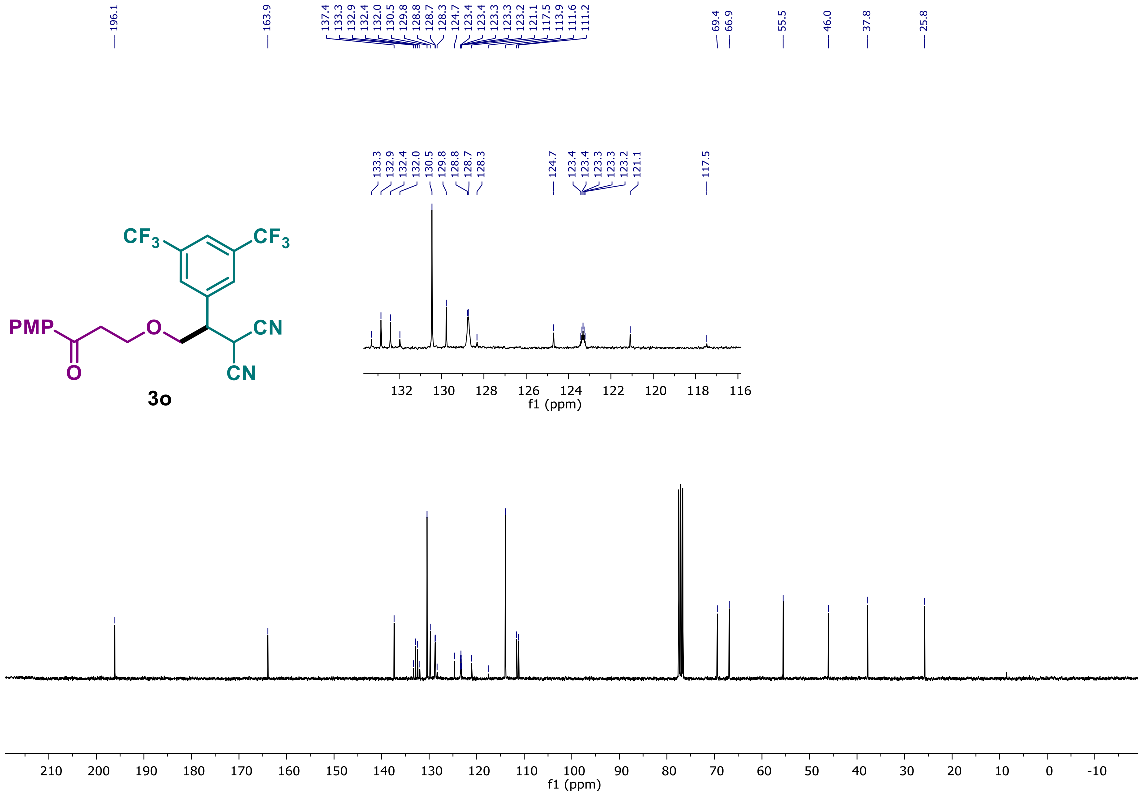

Figure S65. ^{13}C NMR spectrum (75 MHz, 298K, $CDCl_3$) of **3o.**

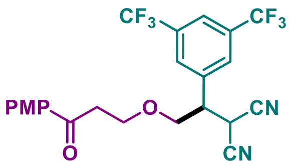

3o

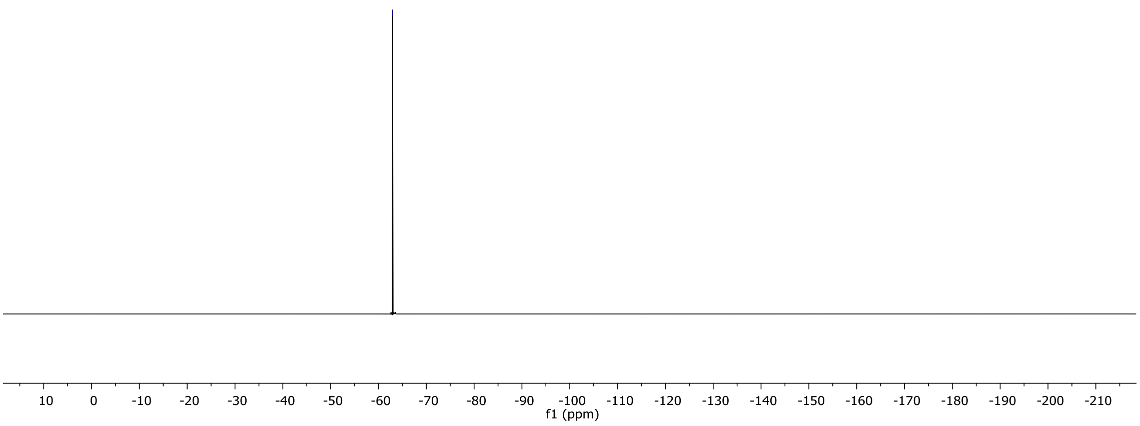

Figure S66. ^{19}F NMR spectrum (282 MHz, 298K, $CDCl_3$) of **3o**.

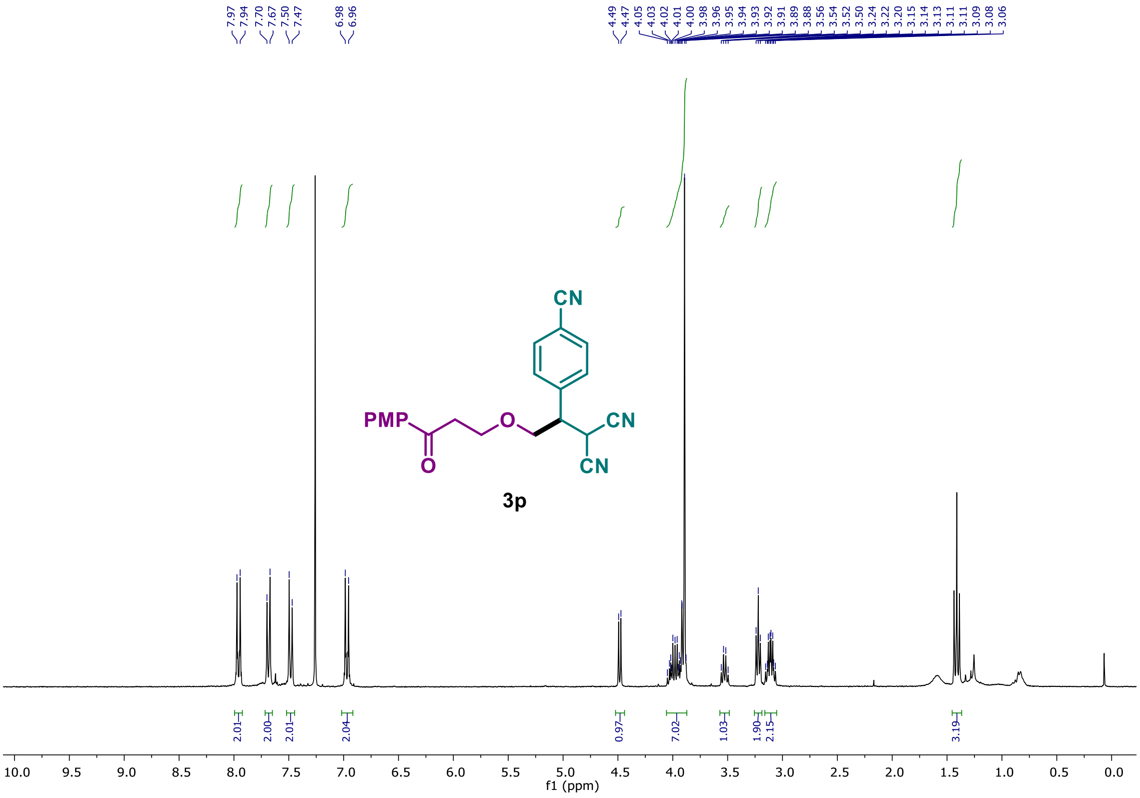

Figure S67. ^{1}H NMR spectrum (300 MHz, 298K, $CDCl_3$) of **3p.**

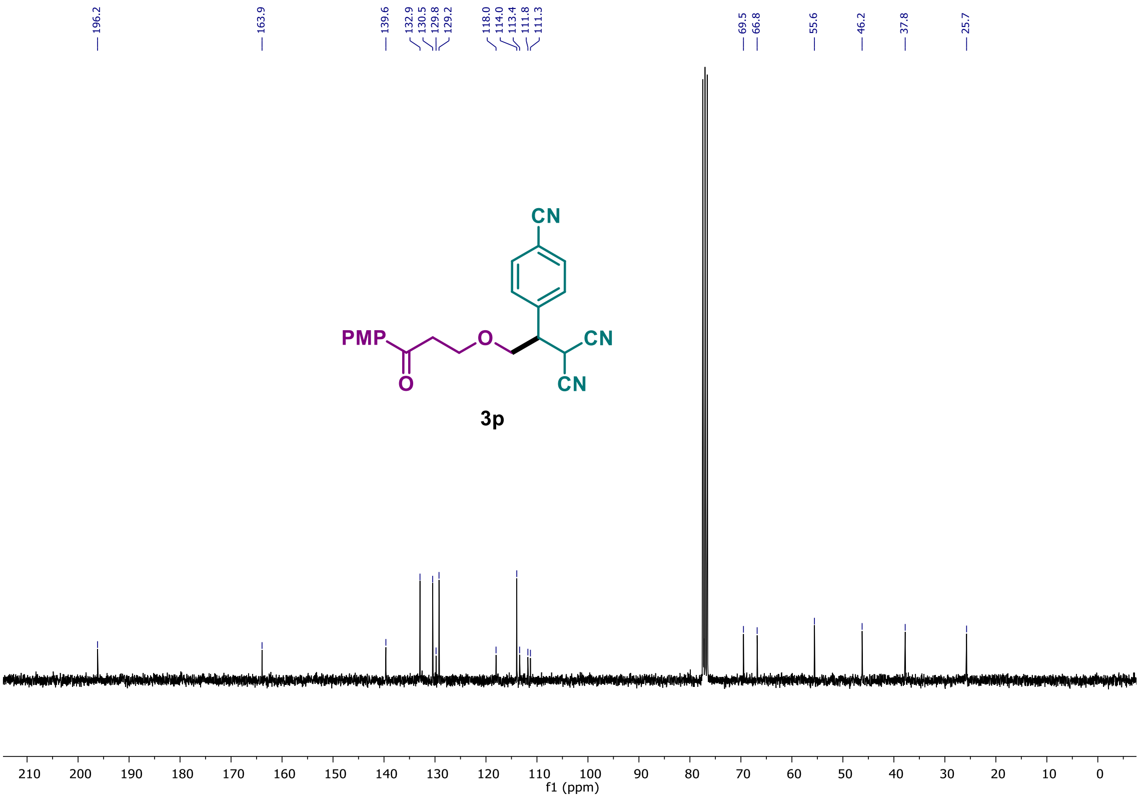

Figure S68. ^{13}C NMR spectrum (75 MHz, 298K, $CDCl_3$) of **3p.**

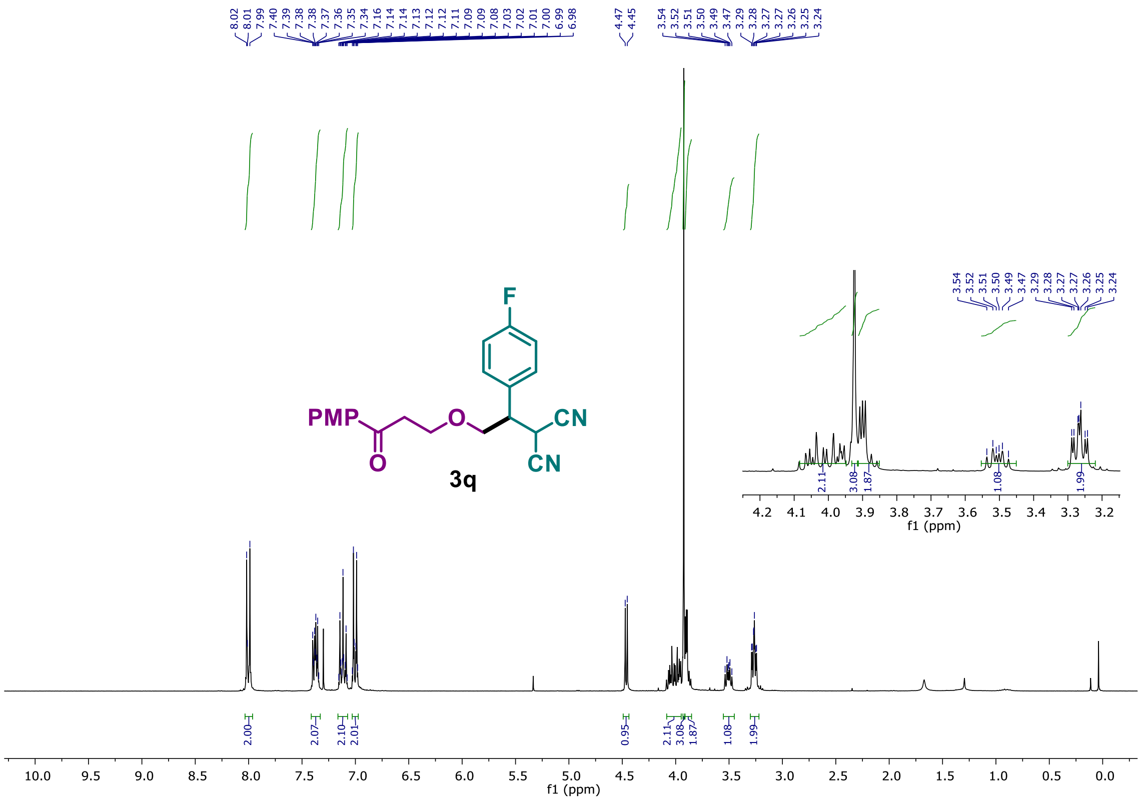

Figure S69. ^{1}H NMR spectrum (300 MHz, 298K, $CDCl_3$) of **3q.**

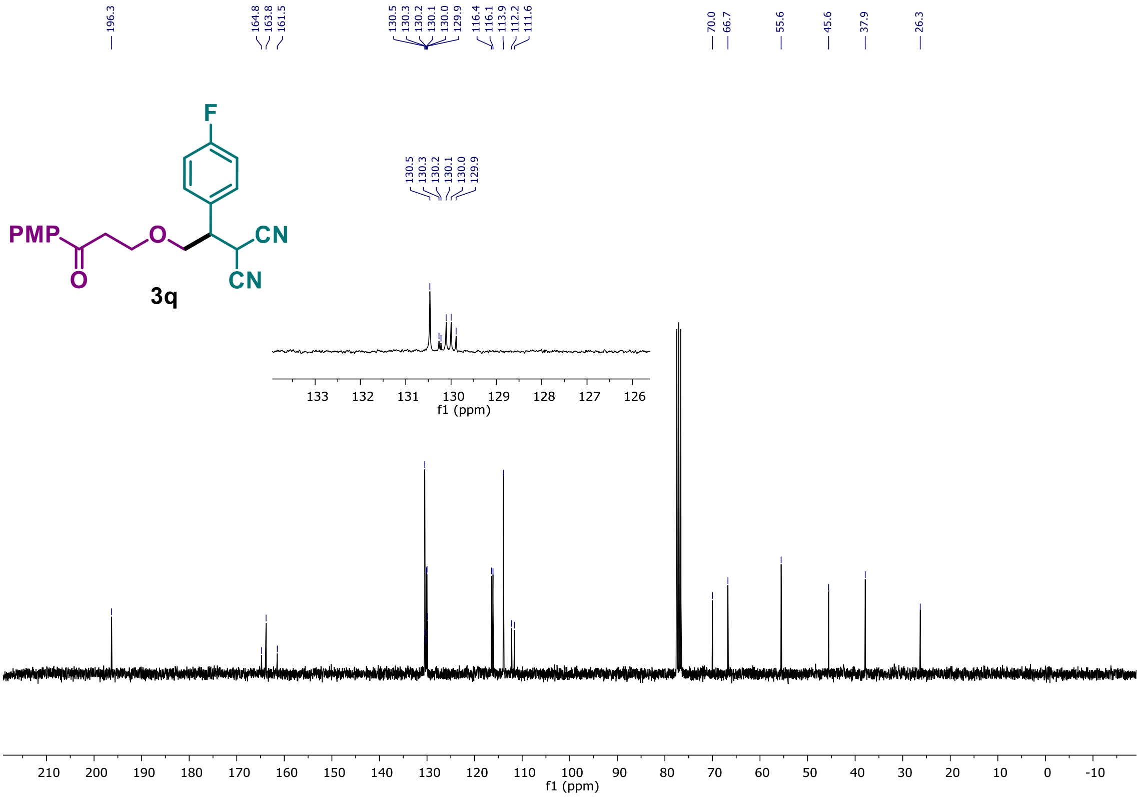

Figure S70. ^{13}C NMR spectrum (75 MHz, 298K, $CDCl_3$) of **3q**

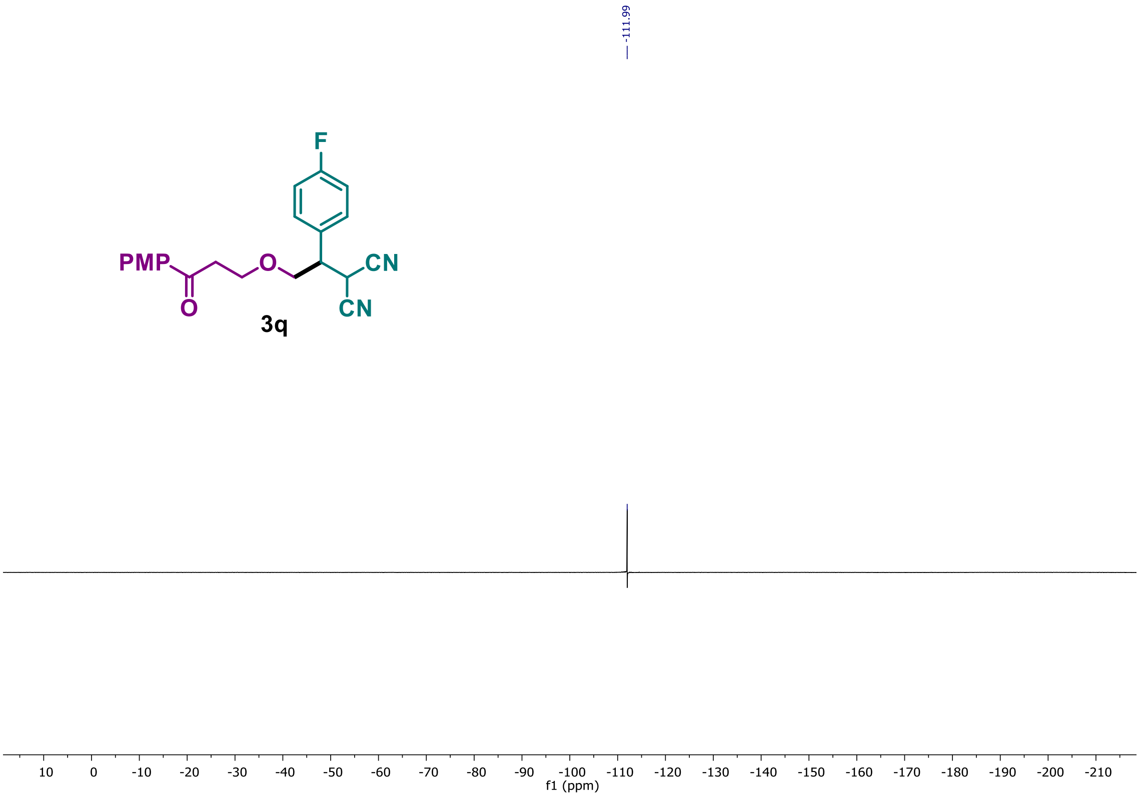

Figure S71. ^{19}F NMR spectrum (282 MHz, 298K, $CDCl_3$) of **3q**.

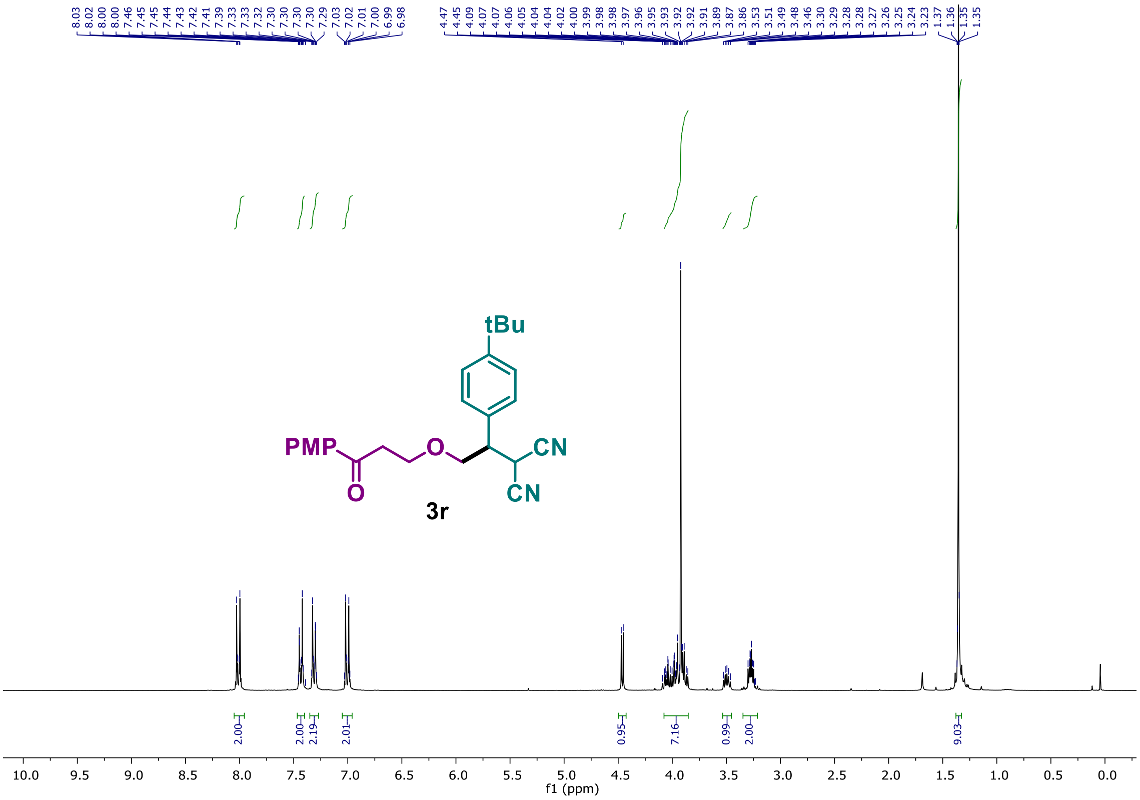

Figure S72. ^{1}H NMR spectrum (300 MHz, 298K, $CDCl_3$) of **3r.**

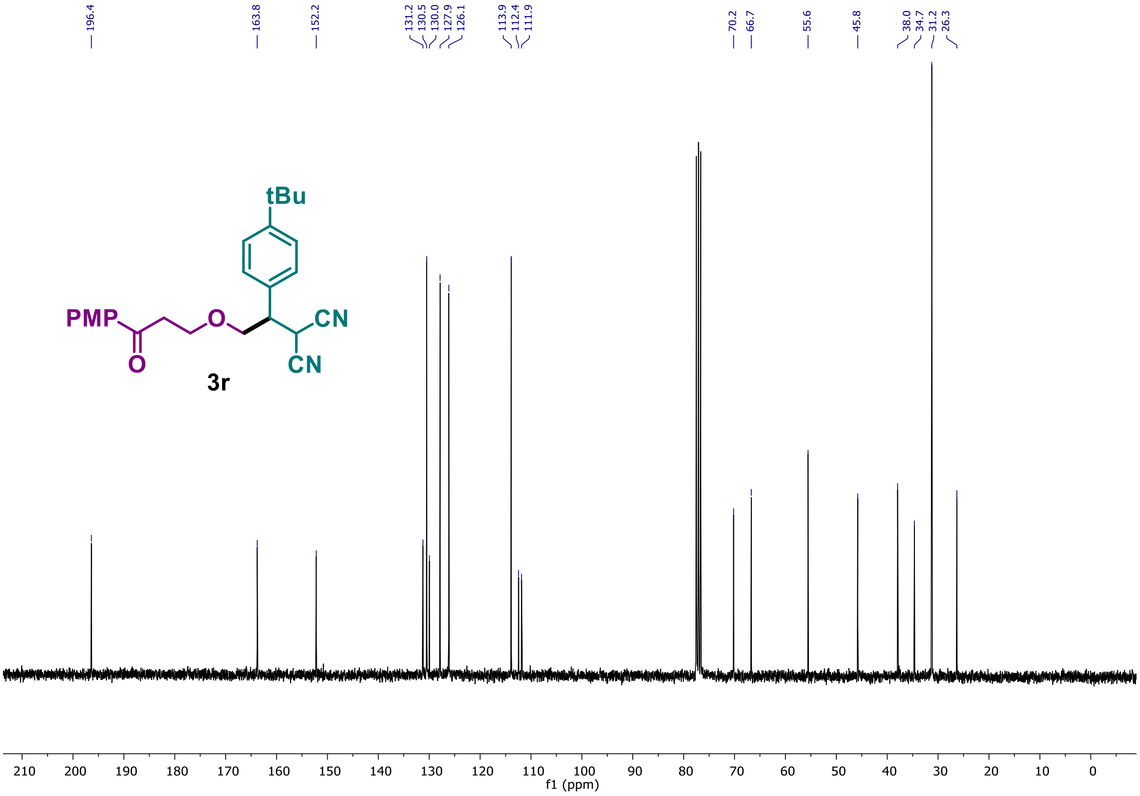

Figure S73. ^{13}C NMR spectrum (75 MHz, 298K, $CDCl_3$) of **3r.**

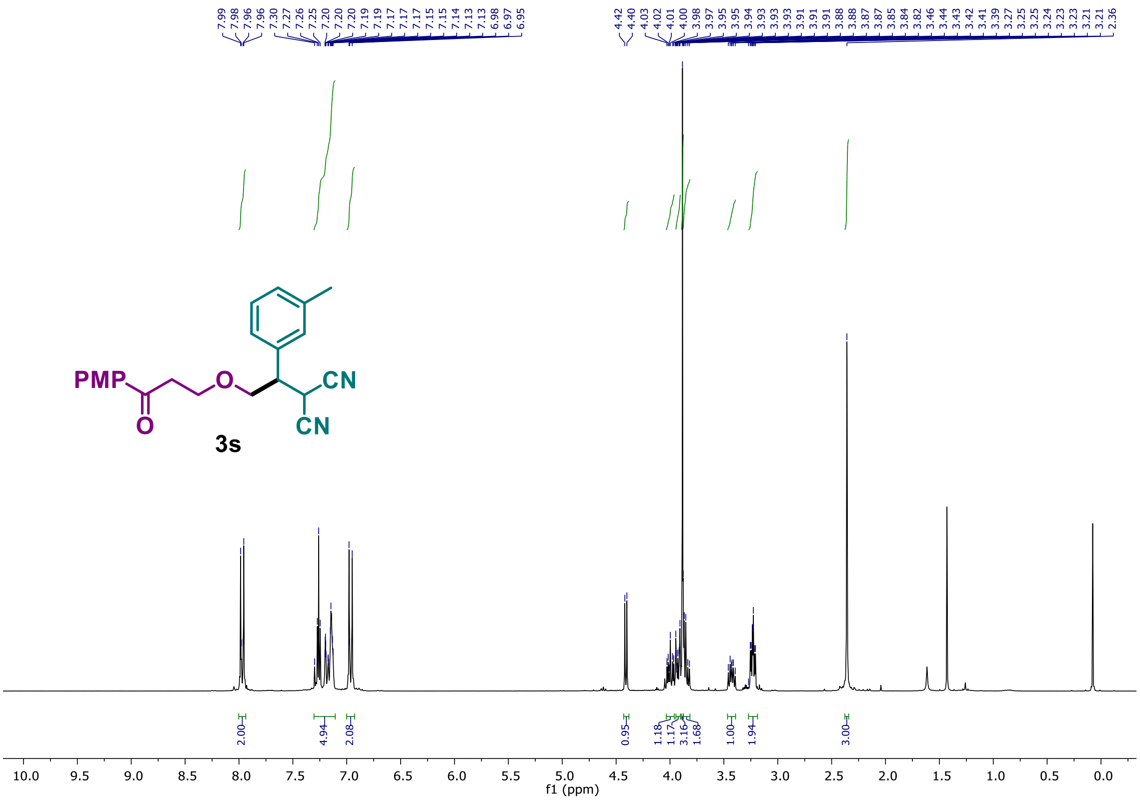

Figure S74. ^{1}H NMR spectrum (300 MHz, 298K, $CDCl_3$) of **3s.**

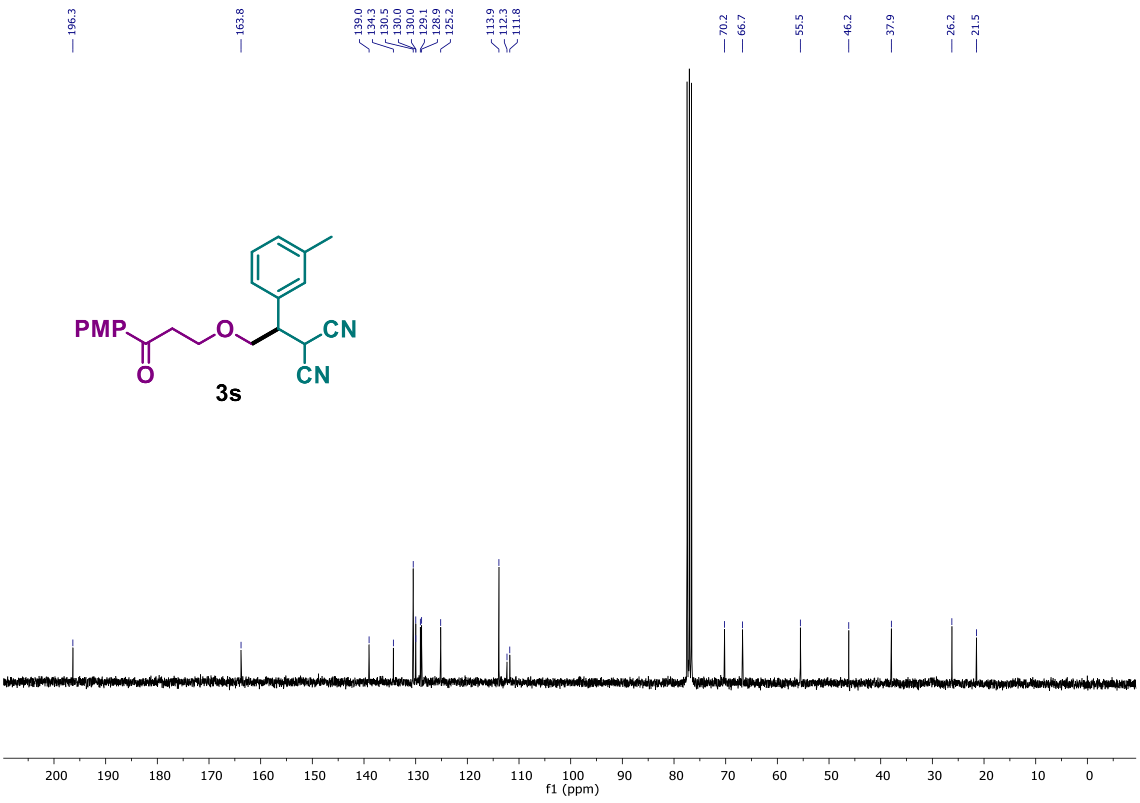

Figure S75. ^{13}C NMR spectrum (75 MHz, 298K, $CDCl_3$) of **3s.**

10 References

[1] Yayla, H. G., Wang, H., Tarantino , K. T.; Orbe, H. S., Knowles, R. R. *J. Am. Chem. Soc*. **2016**, 138, 34, 10794–10797.

[2] R. A. Croft, M. A. J. Dubois, A. J. Boddy, C. Denis, A. Lazaridou, A. S. Voisin-Chiret, R. Bureau, C. Choi, J. J. Mousseau, J. A. Bull, *Eur. J. Org. Chem*. **2019**, 31-32, 5385.

[3] Salaverri, N., Carli, B., Díaz-Tendero, S., Marzo, L. & Alemán, J. *Org. Lett*. **2022,** 24, 3123

[4] Wu, S., Li, J., He, R., Jia, K. & Chen, Y. *Org. Lett*. **2021,** 23, 9204.

[5] Hareram, M. D. et al. *Org. Lett*. **2022**, 24, 3890.

[6] Salaverri, N., Carli, B., Gratal, P. B., Marzo, L. & Alemán, J. *Adv Synth Catal*, **2022**, 364, 1689.

[7] De Abrantes, P. G., De Abrantes, P. G., Ferreira, J. M. G. D. O. & Vale, J. A., *Synthetic Communications,* **2023** 53, 135.

[8] Sultana, S., Kumar, G., Sarma, L. S., Venkatramu, V. & Gangi Reddy, N. C. *Eur J Org Chem*, **2023**, 26, e202300032.

[9] Ang, W. J.; Chng, Y. S.; Lam, Y. *RSC Adv*. **2015**, 5, 81415.

[10] 1. Tukhtaev, H. B. et al. *Org. Lett*., **2019**, 21, 1087.

[11] H. A. Benesi, J. H. Hildebrand, *J. Am. Chem. Soc*. **1949**, *71*, 2703.

[12] Y. Zhao, D. G. Truhlar, *Theor. Chem. Account.* **2008**, *120*, 215.

[13] J. Tomasi, B. Mennucci, R. Cammi, *Chem. Rev.* **2005,** *105*, 2999.

[14] J. A. Montgomery, M. J. Frisch, J. W. Ochterski, G. A. Petersson, *J. Chem. Phys.* **2000**, *112*, 6532.

[15] M. J. Frisch, G. W. Trucks, H. B. Schlegel, G. E. Scuseria, M. A. Robb, J. R. Cheeseman, G. Scalmani, V. Barone, G. A. Petersson, H. Nakatsuji, X. Li, M. Caricato, A. V. Marenich, J. Bloino, B. G. Janesko, R. Gomperts, B. Mennucci, H. P. Hratchian, J. V. Ortiz, A. F. Izmaylov, J. L. Sonnenberg, D. Williams-Young, F. Ding, F. Lipparini, F. Egidi, J. Goings, B. Peng, A. Petrone, T. Henderson, D. Ranasinghe, V. G. Zakrzewski, J. Gao, N. Rega, G. Zheng, W. Liang, M. Hada, M. Ehara, K. Toyota, R. Fukuda, J. Hasegawa, M. Ishida, T. Nakajima, Y. Honda, O. Kitao, H. Nakai, T. Vreven, K. Throssell, J. A. Montgomery, Jr., J. E. Peralta, F. Ogliaro, M. J. Bearpark, J. J. Heyd, E. N. Brothers, K. N. Kudin, V. N.

Staroverov, T. A. Keith, R. Kobayashi, J. Normand, K. Raghavachari, A. P. Rendell, J. C. Burant, S. S. Iyengar, J. Tomasi, M. Cossi, J. M. Millam, M. Klene, C. Adamo, R. Cammi, J. W. Ochterski, R. L. Martin, K. Morokuma, O. Farkas, J. B. Foresman, and D. J. Fox, Gaussian, Inc., Wallingford CT, **2016**.

[16] A. Krech, V. Yakimchyk, T. Jarg, D. Kananovich, M. Ošeka, *Adv Synth Catal, 366*, 91–100, **2024**.
